# A metabolomic signature of weight-loss and its associations with BMI-linked diseases: A post-hoc analysis of two intervention trials and a Mendelian randomisation study

**DOI:** 10.1371/journal.pmed.1005290

**Published:** 2026-11-04

**Authors:** Nicholas Sunderland, Madeleine L. Smith, Alex McConnachie, Paul Welsh, Roy Taylor, Michael E. J. Lean, Chris A. Rogers, Jane M. Blazeby, Naveed Sattar, Lavinia Paternoster, R. Thomas Lumbers, Nicholas J. Timpson, Laura J. Corbin

**Affiliations:** 1 MRC Integrative Epidemiology Unit, Bristol Medical School, University of Bristol, Bristol, United Kingdom; 2 Population Health Sciences, University of Bristol, Bristol, United Kingdom; 3 Robertson Centre for Biostatistics, School of Health and Wellbeing, University of Glasgow, Glasgow, United Kingdom; 4 School of Cardiovascular and Metabolic Health, University of Glasgow, Glasgow, United Kingdom; 5 Newcastle Magnetic Resonance Centre, Institute of Cellular Medicine, Campus for Ageing and Vitality, Newcastle University, Newcastle upon Tyne, United Kingdom; 6 Human Nutrition, School of Medicine, Dentistry and Nursing, College of Medical, Veterinary and Life Sciences, University of Glasgow, Glasgow, United Kingdom; 7 Bristol Medical School, University of Bristol, Bristol, United Kingdom; 8 Exeter Medical School, University of Exeter and Exeter NIHR Biomedical Research Centre, University of Exeter and Royal Devon University Healthcare NHS Foundation Trust, Exeter, United Kingdom; 9 NIHR Bristol Biomedical Research Centre, University Hospitals Bristol NHS Foundation Trust and University of Bristol, Bristol, United Kingdom; 10 Institute of Health Informatics, University College London, London, United Kingdom; Hong Kong Metropolitan University, HONG KONG

## Abstract

**Background:**

Obesity is a major risk factor for a broad spectrum of diseases, yet the molecular pathways underlying this risk remains poorly understood. Individual analytical approaches—interventional trials, observational data, and genetic methods—are each susceptible to distinct sources of bias. We aimed to identify a common metabolic perturbation signature of adiposity by triangulating evidence across multiple sources of body mass index (BMI) variation, and to characterise associations between this signature and contrasting adiposity-linked disease outcomes.

**Methods and findings:**

We integrated mass spectrometry and nuclear magnetic resonance metabolomic profiling from two weight-loss interventions—a structured diet programme (DiRECT trial, ISRCTN03267836) and metabolic and bariatric surgery (By-Band-Sleeve trial, ISRCTN00786323)—with mendelian randomisation (MR) estimates of the effect of lifetime BMI exposure on metabolite levels, in order to identify a consistent BMI-associated metabolite signature across sources with distinct error structures. Weight loss-associated metabolites were then instrumented using genetic variants and their associations with heart failure (HF), osteoarthritis, and kidney cancer estimated in a two-sample MR framework, with instruments grouped into clusters based on genetic similarity to aid biological interpretation. 242 metabolites were consistently altered across weight loss interventions, predominantly comprising lipids, lipoproteins, and amino acids. The majority of these showed concordant associations with lifetime BMI in MR, consistent with regulation by adiposity. Differential components of this signature were associated with disease-specific outcomes. For HF, low-density lipoprotein (LDL) measures were strongly associated with increased risk, consistent with ischaemic aetiology enrichment, whilst high-density lipoprotein (HDL) cholesterol-enrichment traits were protective, and markers of dysfunctional HDL remodelling were associated with increased risk. For osteoarthritis, lipoprotein associations were again associated with disease risk but in the opposite direction to that seen with HF; additional signals were observed in glycine metabolism pathways. For kidney cancer, the adiposity metabolite signature translated poorly into disease associations, with only 5-methyluridine and ursodeoxycholate associations identified. The main limitations of our study include the potential for residual horizontal pleiotropy with metabolite instruments despite multiple sensitivity analyses and the rank-normalised scale of metabolite measures, which limits direct physiological interpretability.

**Conclusions:**

Triangulating evidence across dietary intervention, metabolic and bariatric surgery, and MR of lifetime BMI identified a robust metabolic signature of adiposity. The association of components of this signature with obesity-related outcomes was disease-specific, supporting the notion that not all adiposity-associated metabolic perturbations are uniformly relevant across the spectrum of obesity-related diseases.

## Author summary

**Why was this study done?:**

- Obesity increases the risk of many serious diseases, including heart failure (HF), osteoarthritis, and kidney cancer, but the molecular pathways responsible differ across conditions and remain poorly understood.
- Understanding which specific circulating molecules are altered by adiposity, and which of these associate with clinical outcomes, could identify new therapeutic targets and help explain how weight loss reduces disease risk.

**What did the researchers do and find?:**

- We combined metabolic measurements from two clinical weight-loss studies, a dietary programme and metabolic and bariatric surgery, with genetic analyses of lifetime body weight to identify 242 circulating molecules (metabolites) consistently changed by weight loss, the majority of which were supported by genetic evidence of being regulated by body weight.
- Using genetic methods to test whether these metabolites are associated with disease risk, we found that different subsets of the weight-loss metabolite signature were linked to different diseases, lipoproteins were strongly linked to HF and associated with osteoarthritis but in the opposite direction, whilst the metabolite signature had limited overlap with kidney cancer risk.

**What do these findings mean?:**

- Not all metabolic changes caused by excess body weight are equally relevant across the full spectrum of obesity-related diseases, different diseases appear to be driven by distinct components of the adiposity metabolome.
- The identified metabolites represent biologically plausible candidates for further investigation as therapeutic targets or biomarkers in weight-loss intervention trials.
- Key limitations include: the use of predominantly European-ancestry data, which limits generalisability; and genetic instrument correlation across related metabolites, which prevents precise attribution of effects to individual molecules.

## Abstract 3

Nicolas Sunderland and colleagues combined metabolomic data from weight-loss trials with genetic analyses to identify metabolic features consistently linked to adiposity. The study showed that these metabolic alterations were associated with obesity-related diseases in disease-specific ways, rather than reflecting a single shared pathway across all outcomes.

## Introduction

Body mass index (BMI) is an anthropometric construct, often used as a proxy measure for adiposity and irrefutably associated with health [1]. Higher BMI is a major risk factor for a broad spectrum of conditions, from cardiovascular (CV) disease and heart failure (HF) to osteoarthritis and numerous cancers, yet the mechanisms linking adiposity to these outcomes differ markedly. For some, such as osteoarthritis, mechanical loading is a significant driver [2]; for others, such as obesity-associated cancer, hormonal dysregulation and chronic inflammation are thought to predominate [3]. Whilst the association between obesity and atherosclerotic CV disease can be largely explained by traditional CV mediators such as high blood pressure and hyperlipidaemia, the same is not true of HF and the exact routes from variation in BMI to disease remain unclear [4–6]. This mechanistic heterogeneity suggests that distinct biological components of adiposity may be driving these associations, and that a single unifying explanation is unlikely.

As a measure, BMI is a practical solution to the challenge of estimating excess adiposity at the population level, being derived from simple metrics and with simple associated guidelines [7]. This underpins why BMI remains the most frequently used form of body composition assessment [8], however conceals the challenge of better understanding the link between adiposity and health outcomes. As an example, BMI does not differentiate between fat and lean mass, it is unable to precisely capture fat distribution and makes assumptions as to the multitude of combinations of physical presentation able to deliver equal numeric values in units of kg/m^2^ [9]. Alternative indices have been extensively investigated and measures such as waist hip ratio, or imaging-derived adiposity parameters, can offer better predictive ability in certain scenarios, but ultimately are highly correlated with BMI and less scalable [8,10,11]. In this context, whilst there is clear value in the application of simple and generic measures, there is a role for using intermediate biological traits to characterise the heterogeneity and nuance lying behind important axes of health risk. Theoretically, the outward phenotype may be less important than understanding the underlying biology that BMI summarises, not least because BMI is linked to such a diverse range of diseases, suggesting that distinct components of adiposity may be driving each of these associations [12].

Efficient and cost-effective metabolomic profiling studies offer the opportunity to further examine the underlying biology behind variation in BMI and responses to it. This can be achieved through precise molecular measurement of circulating small molecules which are themselves a by-product of cellular activity. Differences in circulating metabolite profiles between individuals with obesity and those of normal weight may highlight adiposity-related metabolic pathways; however, they may also capture coincident signals related to interventions, medication use, or bystander phenomena such as prevailing health. One strategy to distinguish confounding or coincident metabolic signals from adiposity-specific effects is to examine consistent signatures across contrasting study designs, each with independent (exogenous) sources of BMI variation.

Further to the characterisation of biological events involved in, or coincident with weight change, the molecular characterisation of adiposity can also be used to open up the examination of disease liability. This has been done by using prospective studies of outcomes such as cancer to dissect molecular intermediaries between BMI and disease [13]. Additionally, natural genetic variation in these intermediaries allows for the dissection of potential causal pathways through mendelian randomisation (MR) analyses.

The aim of this study is to characterise the metabolic footprint of adiposity by leveraging separate sources of BMI variation to highlight consistent biology, and to determine which components of this signature are relevant to specific adiposity-linked diseases, using three mechanistically distinct outcomes—HF, osteoarthritis, and kidney cancer—as exemplars. We hypothesise that triangulating weight-loss metabolomics with MR-based disease analyses can identify disease-specific obesity-related metabolic pathways, with implications for targeted prevention and treatment.

## Methods

An overview of the study is presented in Fig 1.

**Fig 1:**
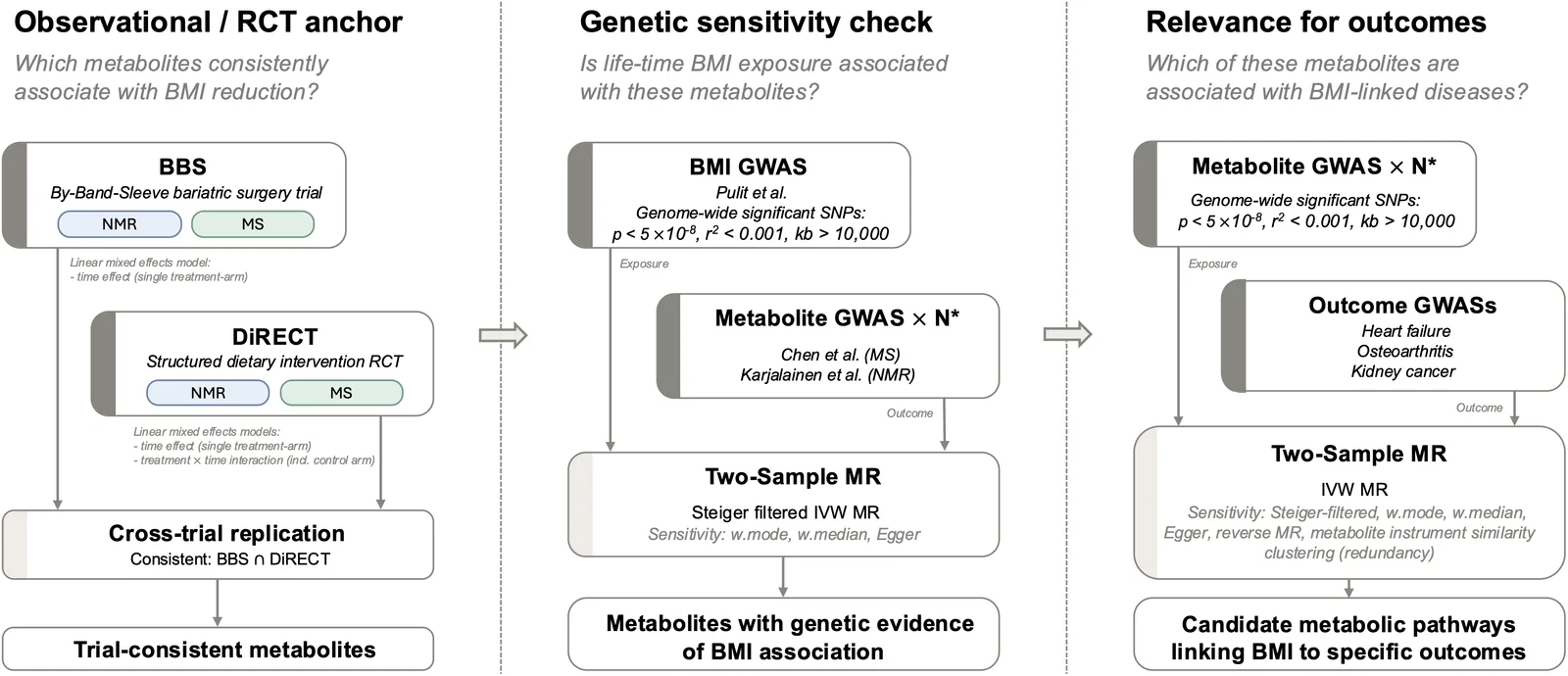
Analysis overview. Metabolite associations with two weight loss interventions (the By-Band-Sleeve metabolic and bariatric surgery trial, BBS, and the DiRECT structured dietary intervention trial) were assessed for consistent direction of effect and strength of association (left panel). To supplement the evidence for these associations being primarily driven by changes in BMI, a mendelian randomisation (MR) analysis was conducted, assessing the impact of life-long BMI exposure on metabolite levels (middle panel). Trial-consistent metabolites were then assessed as to their impact on BMI-linked disease outcomes using MR (right panel). N*, the number of trial-consistent metabolites with available GWAS data for the MR analyses was 233. NMR, nuclear magnetic resonance (Nightingale metabolite profiling platform); MS, mass spectrometry (Metabolon metabolite profiling platform); MR, mendelian randomisation; IVW, inverse-variance weighted; w.mode, weighted mode; w.median, weighted median; BMI, body mass index; SNP, single nucleotide polymorphism.

### Weight loss trial-metabolite associations

#### Study populations.

To identify metabolites associated with adiposity, we examined the effects of two distinct weight-loss interventions—dietary intervention (Diabetes Remission Clinical Trial, DiRECT) and metabolic and bariatric surgery (By-Band-Sleeve Trial, BBS)—on the circulating metabolome. The trials have been described in detail previously [14–18].

Briefly, the DiRECT trial was a 2-year open-label, cluster randomised controlled trial (RCT) conducted at 49 primary care practices in Scotland and the Tyneside region of England between 25 July 2014 and 5 August 2016 (isrctn.org registration no. ISRCTN03267836, https://www.isrctn.com/ISRCTN03267836). Individuals with type 2 diabetes mellitus (T2DM), a BMI of 27–45 kg/m^2^ and who were not receiving insulin, were randomised to either a weight management programme (intervention) or best-practice care by guidelines (control). The intervention comprised withdrawal of glucose-lowering and antihypertensive drugs, total diet replacement (3,452–3,569 kJ [825–853 kcal]/day formula diet for 3–5 months), stepped food reintroduction (2–8 weeks) and structured support for long-term weight-loss maintenance. Blood samples were available for metabolomic profiling at baseline and the endpoint was metabolic profiling at 12 months post-randomisation. Ethics approval for DiRECT was granted by the West of Scotland Research Ethics Committee (reference number: 13/WS/0314) in January 2014, with approvals by the National Health Service (NHS) health board areas in Scotland and clinical commissioning groups in Tyneside. All participants provided written informed consent.

The By-Band-Sleeve trial was a multi-site RCT designed to assess the most effective type of metabolic and bariatric surgery (Roux-en-Y gastric bypass, laparoscopic adjustable gastric band, or sleeve gastrectomy) for weight loss and quality of life (EQ-5D-5L) as well as cost-effectiveness (isrctn.org registration no. ISRCTN00786323, https://www.isrctn.com/ISRCTN00786323). Individuals with a BMI of ≥40 kg/m^2^, or ≥35 kg/m^2^ with other comorbidities such as T2DM, who were fit for general anaesthesia and surgery were recruited and randomised to one of the three types of metabolic and bariatric surgery. Two sample collection efforts were made within By-Band-Sleeve—firstly, from participants in the main RCT and secondly from participants in a nested nonrandomised (observational) sub-study. In both instances, samples were collected from patients at two time points. Ten of the 12 sites that participated in the main RCT collected samples for future research with blood samples available for metabolomic profiling at baseline and 36-months post-randomisation. Seven of the 12 sites collected samples for future research in the nonrandomised sub-study with blood samples available for metabolomic profiling at baseline and 12-months post-surgery. For the analysis presented here, this study is considered a single-arm (any surgery type) pre-post intervention study with the effect on metabolite levels derived from within-subject change over time. Ethical approval for BBS was granted by the Southwest Frenchay Research Ethics Committee (reference 11/SW/0248) and written informed consent was obtained from all participants. All samples were used, stored and disposed of in accordance with the Human Tissue Act 2004.

#### Sample collection and metabolite data acquisition.

Sample handling procedures have been described previously [15,19,20]. Participants were asked to fast overnight before blood draw. All analysts were blinded to intervention status. Samples were sent for metabolomic profiling on the untargeted mass spectrometry (MS) Metabolon Global DIscovery Panel platform (Metabolon, Durham, NC, USA) and the ^1^H-NMR (NMR) Nightingale platform (Nightingale Health, Finland).

#### Metabolite data preparation.

Data relating to participants who withdrew consent during the study were removed from the analysis. Data quality was assessed locally using the *metaboprep* R package and predefined quality control (QC) metrics, as previously described [15,20,21]. Samples with greater than 20% missingness, outlier samples with standard deviation (SD) >5 from the mean based on principal components, and samples > 5 SD from the total peak area (sum of metabolite levels for each individual at metabolites with no missingness), were identified and removed. Metabolites with >20% missingness were also removed.

QC procedures for the By-Band-Sleeve MS dataset led to a total of 1,398 samples with 952 metabolites for analysis, and for the NMR dataset 1,374 samples with 250 metabolites. The same QC procedures in the DiRECT MS dataset led to a total of 557 samples with 927 metabolites, and for the NMR dataset 553 samples with 225 metabolites (S1 and S2 Figs).

MS datasets were transformed using a rank-based inverse normal transformation (INRT, where tied ranks were split by assigning random order) within trial datasets separately. NMR datasets were transformed using Z-score normalisation within trial datasets separately, standardising each metabolite by subtracting the mean and dividing by the SD. Z-score normalisation was used as meaningful units can be preserved with NMR data, whereas the MS data is semi-quantitative.

Agreement between metabolite measurements available on both the MS and NMR platforms was assessed using Bland–Altman analysis [22]. Given the differing standardisation approaches, agreement was evaluated in terms of relative concentration difference (Z-score [NMR] versus INRT [MS]). Limits within ±0.5 SD were considered good agreement, consistent with typical technical platform variation [23].

#### Statistical analysis—weight loss intervention effects on metabolite levels.

To estimate the effect of weight loss on metabolite levels, linear mixed-effects models were fitted separately for each metabolite using the *lmer()* function from the lmerTest R package (Model 1) [24]. This approach was preferred over simple change-scores, as not all participants had measurements at both timepoints. Retaining participants with incomplete pre-post data makes full use of all available observations and increases statistical power relative to a complete-cases analysis but does rely on the assumption that missing observations are missing at random. With only two timepoints the model is broadly equivalent to a change-score analysis for participants with complete pre-post data.

Model 1 was applied to each metabolite in the metabolic and bariatric surgery cohort (a single-arm intervention study) and the intervention arm of the structured dietary intervention RCT. The normalised metabolite levels *M*_*i,t*_ for individual *i* at time point *t* were regressed on the binary time point indicator (baseline or follow up), with subject ID included as a random effect to account for repeated measures. The time point coefficient estimates the mean within-group change in metabolite following weight loss intervention. Models were adjusted for age at baseline (years), sex, site, sample storage time (months), and study-specific covariates. For metabolic and bariatric surgery, a *sub-study* term was included as a study-specific covariate, to account for the participants who were nonrandomly allocated surgery type and in whom follow-up samples were obtained at 12-months post intervention, rather than 36-months post randomisation as in the main cohort. For the structured dietary intervention, the minimisation variable *GP practice list size* (>5,700 or ≤5,700) was included as a study specific covariate.

Model 1:


$$\begin{array}{c} M_{i,\;t}\;\sim \;{timepoint}_{t}\;+\;{age}_{i}\;+\;{sex}_{i}\;+{site}_{i}\;+{storage\;time}_{i,t}\;+\;{study\;specific\;covariates}_{i}\;+\;(\text{1}|{id}_{i}) \end{array}$$

To capitalise on the RCT study design of the structured dietary intervention trial, a second model was fit in these data utilising the control arm (Model 2).

Model 2:


$$\begin{array}{c} \begin{array}{c} M_{i,\;t}\;\sim \;{timepoint}_{t}\;+{allocation}_{i}\;+\;{timepoint}_{t}\cdot {allocation}_{i}\;+\;{site}_{i}\;+\;{list\;size}_{i}\;+\\ \text{\textasciitilde{} }{age}_{i}\;+\;{sex}_{i}\;+\;{storage\;time}_{i,t}\;+\;(\text{1}|{id}_{i}) \end{array} \end{array}$$

The primary statistic of interest in Model 2 was the time point-by-allocation interaction term and its associated *P*-value, identifying metabolites that changed differentially between treatment and control arms, confirming that observed changes in Model 1 were not solely attributable to the passage of time [25].

#### Assessment of weight loss effect consistency between trials.

To distinguish metabolite changes associated with BMI reduction from intervention-specific effects, we first assessed the consistency of effect between our two primary sources of evidence—dietary intervention and metabolic and bariatric surgery. Our interpretation of this overlap is that the most parsimonious explanation of shared effects is that they are driven by the most prominent joint outcome—intentional weight loss—which is also manifest as differences in BMI. Consistent effects were defined as metabolites demonstrating the same direction of association across interventions and those that reach a *P*-value threshold of < 0.05 after Benjamini and Hochberg false discovery rate (FDR) correction (applied within studies) [26]. The estimates compared for effect direction concordance were those from the intervention-only analyses (time point coefficient from Model 1). The strength of association filter (*P*-values) was based on the same time point coefficient for the single-arm metabolic and bariatric surgery study, however, for the dietary intervention trial was based on the *P*-value from the time point:allocation interaction term. This hybrid approach incorporates information from the control arm of the dietary intervention RCT into the selection process, whilst maintaining comparable intervention estimates.

### BMI-metabolite MR

To determine whether metabolites showing consistent effects across both weight loss interventions also have genetic support for an effect of BMI on their levels, we performed two-sample MR using genetic variants strongly associated with BMI as instruments and metabolite genetic associations as outcomes. This study is reported as per the Strengthening the Reporting of Observational Studies in Epidemiology using MR (STROBE-MR) guideline (S1 Checklist) [27].

#### Genome-wide association study data sources.

The genome-wide association study (GWAS) data sources are detailed in S1 Table. A genetic instrument for BMI was derived from genome-wide association summary statistics from the Genetic Investigation of Anthropometric Traits (GIANT) consortium and UK Biobank meta-analysis of 806,834 individuals of predominantly European ancestry [28,29]. GWAS summary statistics for metabolites on the NMR and MS platforms were obtained from the studies by Karjalainen and colleagues and Chen and colleagues, respectively [30,31].

No metabolite GWAS included UK Biobank participants, so the UK Biobank component of the BMI instrument contributes no sample overlap. Several of the GIANT studies also contribute to the NMR metabolite GWAS. Assuming complete overlap within each shared cohort, at most 28,933 individuals were common to both samples, representing 4% of the BMI GWAS and 21% of the metabolite GWAS (S1 Table). Sample overlap can bias two-sample MR estimates toward the confounded observational association, although the bias is minimal when instrument strength is high. The GIANT meta-analysis was retained as the primary instrument given its greater power, however, we performed a sensitivity analysis using BMI instruments derived from UK Biobank alone (*n* = 361,194, Neale Lab) [32], which share no participants with either metabolite GWAS.

#### Selection of instruments.

Variants for inclusion in the BMI instrument were selected based on a genome-wide association study *P*-value threshold of *P* < 5 x 10^−8^. Variants were clumped to account for linkage disequilibrium using PLINK 2.0 (r^2^ < 0.001, 10,000 kb) with a reference panel consisting of 10,000 unrelated European ancestry participants from the UK Biobank [33,34]. Exposure and outcome summary statistics were harmonised using the TwoSampleMR R package (action = 2), which infers positive-strand alleles for palindromic single nucleotide polymorphisms (SNPs) using allele frequencies, excluding those where the allele frequency is between 0.42 and 0.58 or variants that could not be harmonised. Instrument strength was assessed using the F-statistic. [35]

#### MR analysis.

MR was performed using a two-sample framework and the TwoSampleMR R package [35]. The genetic instrument used in MR should satisfy three core instrumental variable assumptions: (i) relevance – each variant is robustly associated with BMI, confirmed through genome-wide significance and large F-statistic (ideally F>> 10); (ii) independence – there are no measured or unmeasured confounders of the instrument and the outcome; and (iii) exclusion restriction – the variants affect outcomes only through BMI, not via alternative biological pathways.

The primary analysis used an inverse variance weighted (IVW) estimator, representing the change in metabolite levels (in SD units) per 1 SD increase in BMI. To assess robustness to pleiotropy, sensitivity analyses were conducted using MR-Egger, weighted median, and weighted mode estimators. Additionally, estimators were run with Steiger filtering to exclude genetic variants that explain more variance in the metabolite than in BMI, as these are more likely to act primarily on the metabolite rather than through BMI, violating the exclusion restriction assumption.

### Metabolite-outcome MR

To test whether weight loss-associated metabolites influence disease risk, we performed two-sample MR using metabolite levels as exposures and three contrasting BMI-linked diseases as outcomes: HF, hip/knee osteoarthritis, and kidney cancer. Instrument selection, harmonisation, and sensitivity analyses followed the same procedure as described above, with IVW as the primary estimator or Wald ratio for only a single variant, using the TwoSampleMR R package [35]. Additionally, MR-PRESSO was applied to detect and correct for outlier variants driving heterogeneity in the IVW estimate, with the outlier-corrected estimate reported where the global heterogeneity test *P*-value was <0.05 [36]. Supporting evidence for the primary result was defined as ≥2 of 4 sensitivity estimators (weighted median, weighted mode, MR-Egger, and MR-PRESSO) showing an effect estimate in the same direction at nominal *P*-valve threshold < 0.05. MR estimates represent the change in log odds of the outcome per one standard deviation increase in genetically predicted lifetime metabolite exposure on the INRT scale. Estimates are exponentiated and reported as odds ratios (OR) per SD.

#### Genome-wide association study data sources.

Metabolite GWAS summary statistics were obtained from Karjalainen and colleagues and Chen and colleagues, as described above [30,31]. Outcome GWAS summary statistics were obtained from the HERMES consortium for all-cause HF (cases/controls: 139,533/1,568,809) [37]; Hatzikotoulas and colleagues for hip/knee osteoarthritis (213,839/1,080,482) [38]; and Purdue and colleagues for kidney cancer (25,890/743,585) [39]. Full details are provided in S1 Table.

#### Instrument similarity and clustering.

Constructing genetic instruments for circulating metabolite levels has well-described challenges due to high intercorrelation between metabolites and shared genetic aetiology [31]. Variants selected to instrument a given metabolite may also associate with other metabolites through vertical or horizontal pleiotropy, the latter of which can bias MR estimates.

To characterise instrument overlap, we calculated the Szymkiewicz–Simpson overlap coefficient, pairwise between all metabolite instruments *G*_*i*_ and *G*_*j*_:


$$\begin{array}{c} {Overlap\;coefficient}_{i,j}=\;\frac{\left|G_{i}\;\cap \;G_{j}\right|}{min\left(\left|G_{i}\right|,\left|G_{j}\right|\right)} \end{array}$$

This captures similarity regardless of instrument size, flagging metabolite pairs where either instrument has a substantial proportional of its SNPs shared with the other. Instruments were clustered using the Louvain community detection algorithm applied to a weighted similarity graph [40], where edge weights reflect the overlap coefficient. Edges were retained for clustering at a threshold of ≥ 0.25 (i.e., at least 25% of the smaller instrument’s SNPs are shared with the other instrument). Cluster membership was used to contextualise results and flag redundancy between metabolites effect estimates on disease outcomes.

#### AI tools and technologies statement.

A large language model (Claude Code v2.1.263, Anthropic) was used to assist in generating the R code that produced the circos plots (Figs 3 and 4). R code utilising the circlize R package [41] was developed against variable names and summary-level structures; no individual-level participant data were provided to, or accessible by, the model. All code was read, executed line by line and output checked against the underlying data by the lead author. The authors take full responsibility for the content of the manuscript.

## Results

### Weight loss study characteristics

The baseline characteristics for participants enrolled in DiRECT and By-Band-Sleeve, for which metabolite profiling was available, are presented in Table 1. With dietary intervention, at 12 months, mean body weight had fallen by 11.1 kg in the intervention group and by 1.4 kg in the control group (intervention associated BMI reduction of 3.7 kg/m^2^). With metabolic and bariatric surgery, over median follow up of 36 (IQR 13–37) months, mean body weight fell by 29.3 kg, a BMI reduction of 10.5 kg/m^2^.

**Table 1:** Weight loss trial characteristics.

Characteristic	Structured diet programme (DiRECT)				Metabolic and bariatric surgery (By-Band-Sleeve)^			
	Control arm		Intervention arm		Main (randomised) study		Nonrandomised study	
Timepoint	Baseline (*n* = 147)	Follow-up 1-year (*n* = 146)	Baseline (*n* = 142)	Follow-up 1-year (*n* = 122)	Baseline (*n* = 969)	Follow-up 3-years (*n* = 369)	Baseline (*n* = 44)	Follow-up 1-year (*n* = 44)
Age, years (SD)	56.0 (7.2)	56.2 (6.9)	52.8 (7.6)	53.8 (7.1)	47 (10.8)	49 (10.1)	45 (10.4)	45 (10.4)
Sex, male (%)	92 (63)	90 (62)	80 (56)	70 (57)	250 (26)	98 (27)	6 (14)	6 (14)
Weight, kg (SD)	99.2 (15.9)	97.8 (16.4)	101.1 (17)	90 (16.8)	130.6 (23.4)	102.1 (23.8)	125.2 (21.0)	93.0 (22.5)
BMI, kg/m^2^ (SD)	34.3 (4.2)	33.9 (4.5)	35.0 (4.5)	31.3 (5.0)	46.6 (6.7)	36.3 (7.3)	45.4 (5.8)	33.6 (6.5)

Abbreviations: SD, standard deviation; kg, kilograms; m, metres.

* Numbers may differ from the published trials as these data relate to the individuals with metabolomic data passing quality control checks.

^ All types of metabolic and bariatric surgery from the By-Band-Sleeve were combined into one cohort and analysed together. There was no control arm and all individuals included underwent surgery.

### Metabolites associated with weight loss intervention

The serum levels of 784 metabolites out of 1,202 (65%) were different following metabolic and bariatric surgery after FDR adjustment using the Benjamini–Hochberg procedure (FDR *P*-value < 0.05). 310 demonstrated higher levels and 474 lower levels after intervention (S2 Table). Dietary intervention was associated with alterations in 490 of 1,152 metabolites (43%) at an FDR < 0.05, with 247 demonstrating higher levels and 243 lower levels (S2 Table).

Of 1,422 metabolites passing QC across both studies, 932 (66%) were measured in both interventions and available for cross-trial comparison. Inter-study correlation of intervention effects was moderate (NMR platform: r = 0.63 (95% CI [0.55,0.71]; *p* < 0.001); MS platform: r = 0.54 (95% CI [0.49,0.59]; *p* < 0.001). Among the 33 metabolites measured on both platforms, intra-study agreement was high (metabolic and bariatric surgery: r = 0.98 (95% CI [0.94,0.99]; *p* < 0.001); dietary intervention: r = 0.89 (95% CI [0.72,0.96]; *p* < 0.001)). As rank-normalised (MS) and z-score normalised (NMR) effect sizes are not directly comparable in magnitude, Bland-Altman analysis (mean difference 0.01; 95% limits of agreement −0.29 to 0.32) should be interpreted as reflecting consistency in the relative ordering and direction of effects across platforms rather than absolute magnitude equivalence (S3–S5 Figs).

### Cross-trial consistency of metabolite associations

To identify metabolites that change with intentional weight loss regardless of intervention type, we compared effect estimate direction and strength of association across metabolic and bariatric surgery and the structured dietary intervention. Of the 932 metabolites measured in both, 645 (69%) showed consistent effect direction, and 242 (26%) demonstrated both consistent direction and strong evidence of association (FDR *P*-value < 0.05) (Fig 2 and S2 Table). Trial-consistent metabolites were predominantly from the lipid, amino acid, and lipoprotein classes (29%, 25%, and 23%, respectively). The metabolites with the largest weight-loss intervention-associated level increases included 1-oleoyl-GPC (18:1), 1-(1-enyl-palmitoyl)-2-oleoyl-GPC (P-16:0/18:1), and Cholesterol esters in large HDL. The metabolites with the greatest weight-loss intervention-associated level decreases included valine, Behenoyl dihydrosphingomyelin (d18:0/22:0), and phospholipids to total lipids ratio in chylomicrons and extremely large very low-density lipoprotein.

**Fig 2:**
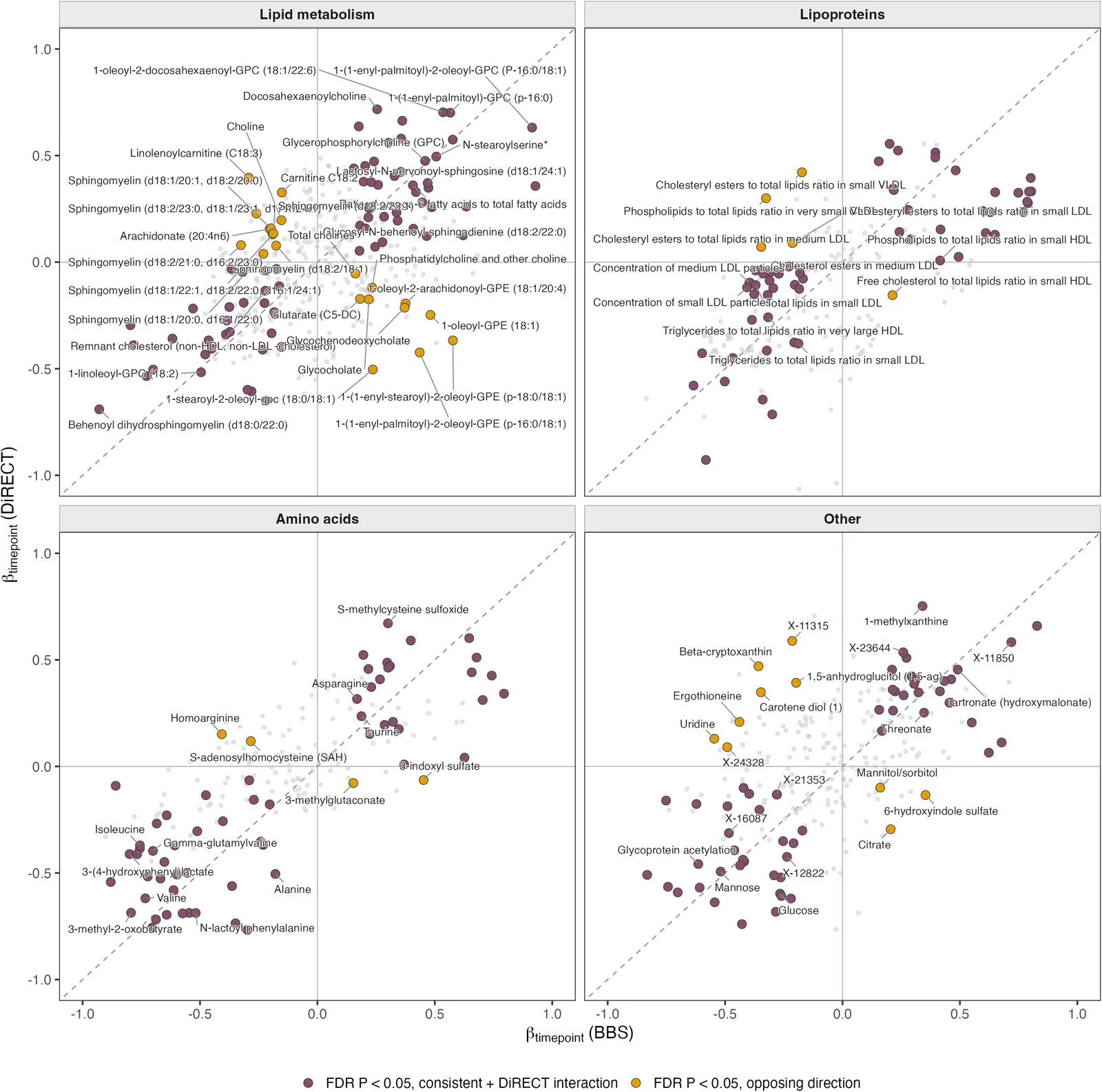
Comparison of estimates for the effect of weight loss intervention on metabolites. Scatterplot comparing estimated effects of metabolic and bariatric surgery (x-axis) and dietary intervention (y-axis) on circulating metabolite levels. Each point represents a metabolite. Nonoverlapping metabolite names are labelled. Purple circles indicate metabolites with consistent directions of effect and association strengths below the FDR-adjusted *P*-value threshold in both interventions; yellow circles indicate metabolites with opposite directions of effect and association strengths below the FDR-adjusted *P*-value threshold; grey points represent all other tested metabolites.

### BMI reduction drives trial metabolite variation: Genetic evidence

Although consistency across two mechanistically distinct weight loss interventions suggests that the observed metabolite changes are likely driven by BMI reduction, shared nonadiposity factors cannot be excluded. As a complementary strand of evidence, we performed two-sample MR using genetic variants to proxy lifelong BMI exposure (S3 Table), restricting analyses to the 242 trial-consistent metabolites.

The MR estimates represent the effect of a genetically proxied 1 SD (~ 4.8 kg/m^2^) higher lifetime BMI on metabolite levels—a positive estimate indicates higher BMI increases a metabolite, a negative estimate that higher BMI decreases a metabolite. Since the weight loss trials capture the effect of BMI reduction, agreement is indicated by discordance between trial and MR estimates. The BMI-metabolite MR estimates are presented in Fig 3 and S4 Table.

**Fig 3:**
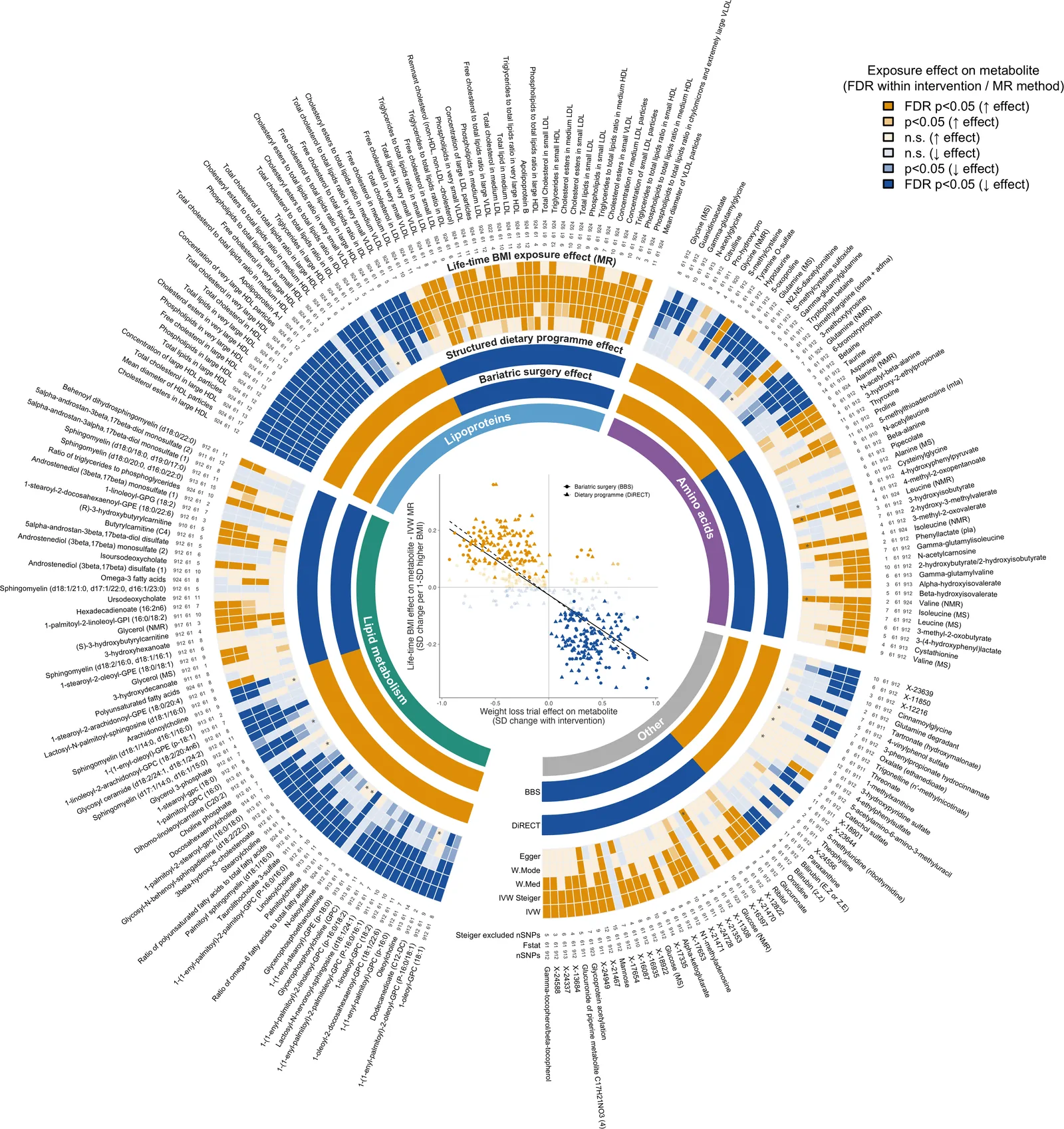
Comparison of estimates for the effect of weight loss intervention and life-time body mass index exposure on metabolites. Circos plot summarising the effects of life-time BMI exposure on circulating metabolites (weight loss trial-replicating metabolites only; see Methods). Metabolites are arranged around the circle and grouped into four pathway sectors by the innermost coloured band (teal: lipid metabolism; light blue: lipoproteins; purple: amino acids; grey: other). Annotation tracks (outer to inner) show, for each metabolite: number of SNPs in the BMI instrument (nSNPs), instrument F-statistic (Fstat), and number of SNPs excluded by Steiger filtering (Steiger excluded nSNPs). The MR heatmap ring displays results from five MR methods (IVW, IVW Steiger filtered, Weighted Median, Weighted Mode, and MR-Egger), with cell colour indicating the direction and significance of the BMI effect on the metabolite: orange shades denote a positive effect (metabolite increases with higher BMI) and blue shades denote a negative effect (metabolite decreases), with full colour saturation indicating FDR-adjusted *p* < 0.05, half saturation indicating nominal *p* < 0.05, and pale colour indicating *p* ≥ 0.05. An asterisk (*) overlaid on the MR-Egger cell denotes a significant Egger intercept (*p* < 0.05). The two innermost heatmap rings show the direction of effect from the dietary intervention (DiRECT) and metabolic and bariatric surgery (BBS) studies, respectively, where orange indicates a positive association and blue a negative association with weight loss. FDR correction is applied within each MR method. The central scatter plot comparing weight-loss intervention effects on metabolites (x-axis; SD change) against life-time BMI MR estimates (y-axis; SD change per 1-SD higher BMI, IVW). Each point represents one metabolite; circles indicate metabolic and bariatric surgery and triangles indicate the structured dietary programme. Point colour indicates the direction and significance of the BMI MR effect, as above. Regression lines are fitted separately per intervention. A negative slope indicates directional concordance between weight-loss-induced and genetically instrumented BMI effects on metabolites.

Among the 242 metabolites with consistent effects across both weight loss interventions, 233 (96%; 72 NMR, 161 MS) had available genetic data. At nominal significance (*P* < 0.05), 165 metabolites showed MR evidence of BMI association, of which 162 (98%) agreed with the weight loss trial effect direction (Fig 3). At the stricter FDR-adjusted threshold, 157 metabolites had strong MR evidence, rising to 176 after Steiger filtering—the increase suggesting that some BMI instruments were attenuating estimates toward the null. Where the IVW-MR demonstrated strong association (FDR *P*-value < 0.05), sensitivity analyses generally confirmed the direction of effect (Fig 3).

To confirm that the residual sample overlap between the primary BMI GWAS (GIANT and UK Biobank) and the NMR metabolite GWASs had not biased the 72 NMR metabolite estimates, we repeated the MR using instruments derived from UK Biobank alone, which share no participants with the NMR GWASs. The overlapping cohorts contribute only to the GIANT component of the BMI instrument, which represents a minority of the total sample. Estimates from the two instrument sets were near-identical (r = 0.996; S6 Fig), indicating the primary results are robust to this sample overlap.

The strongest BMI-increasing metabolite effect was seen for mannose, whilst the strongest BMI-decreasing effect was seen for 1-(1-enyl-palmitoyl)-2-oleoyl-GPC (P-16:0/18:1); weight loss intervention reduced and increased these metabolites, respectively, consistent with these directions of effect. Only three metabolites demonstrated strong effects opposite to the expected direction between weight loss intervention and lifetime higher BMI exposure: dimethylarginine, tyramine O-sulfate and the caffeine metabolite 5-acetylamino-6-amino-3-methyluracil. Each was positively associated with lifetime BMI by MR, yet circulating levels rose rather than fell following weight loss intervention.

### Weight loss metabolite associations with disease outcomes

Excess adiposity is associated with a broad spectrum of diseases and the benefits of intentional weight loss have been demonstrated across multiple health domains. However, the metabolic footprint of adiposity is unlikely to be uniformly relevant across disease contexts. To explore this, we performed MR analyses examining the effects of our weight-loss associated metabolites on three mechanistically distinct BMI-linked outcomes: HF, hip/knee osteoarthritis, and kidney cancer.

### Metabolite instrument characterisation and clustering

To contextualise the MR results, we first assessed the degree to which metabolite genetic instruments overlap with one another. Genome-wide instruments were available given the prespecified clumping parameters for 211 of the 242 (87%) weight-loss associated metabolites (S5 Table). Instrument sizes (number of variants) ranged from 1 to 111, with a median F-statistic of 120 (IQR 67–146). The 211 metabolites instruments yielded 22,155 unique pairs, of which 3,303 (15%) had at least 1 variant in common and 1,230 (6%) had an overlap coefficient of ≥25%.

To identify redundancy between metabolite genetic instruments, graph clustering was performed by overlap similarity coefficient (edge weight). Cluster stability across graph edge pruning thresholds demonstrated the highest stability around a value of 0.25, i.e., retaining metabolites for clustering with overlap coefficients ≥ 25% (S7 Fig). Clustering at this threshold resulted in 17 nonsingleton clusters (cluster size range 2–39) and 64 singletons (S5 Table, S8 and S9 Figs). The largest cluster (cluster 7) comprised 39 metabolite instruments from across the metabolite classes, including lipid molecules, measures of high-, intermediate-, low- and very-low-density lipoprotein (HDL, IDL, LDL, and VLDL) and amino acids; the median similarity coefficient between cluster members was 17% (IQR 0%–32%). The second and third largest clusters comprised primarily LDL (cluster 34; median similarity 38%, IQR 25%–63%) and HDL (cluster 30; median similarity 33%, IQR 25%–48%) measures, respectively (S5 Table).

### Disease-specific weight loss metabolite associations

In the primary IVW-MR analysis, 30 metabolite traits were associated with all-cause HF at an FDR-adjusted *P* value < 0.05 (Fig 4 and S6 Table). These were predominantly lipid, VLDL, and LDL lipoprotein measures associated with increased HF risk, consistent with a phenotype enriched for atherosclerotic/ischaemic aetiology. All LDL trait associations were derived from instruments that clustered together in terms of similarity (cluster 34), suggesting they reflect shared genetic signal rather than independent causal effects.

**Fig 4:**
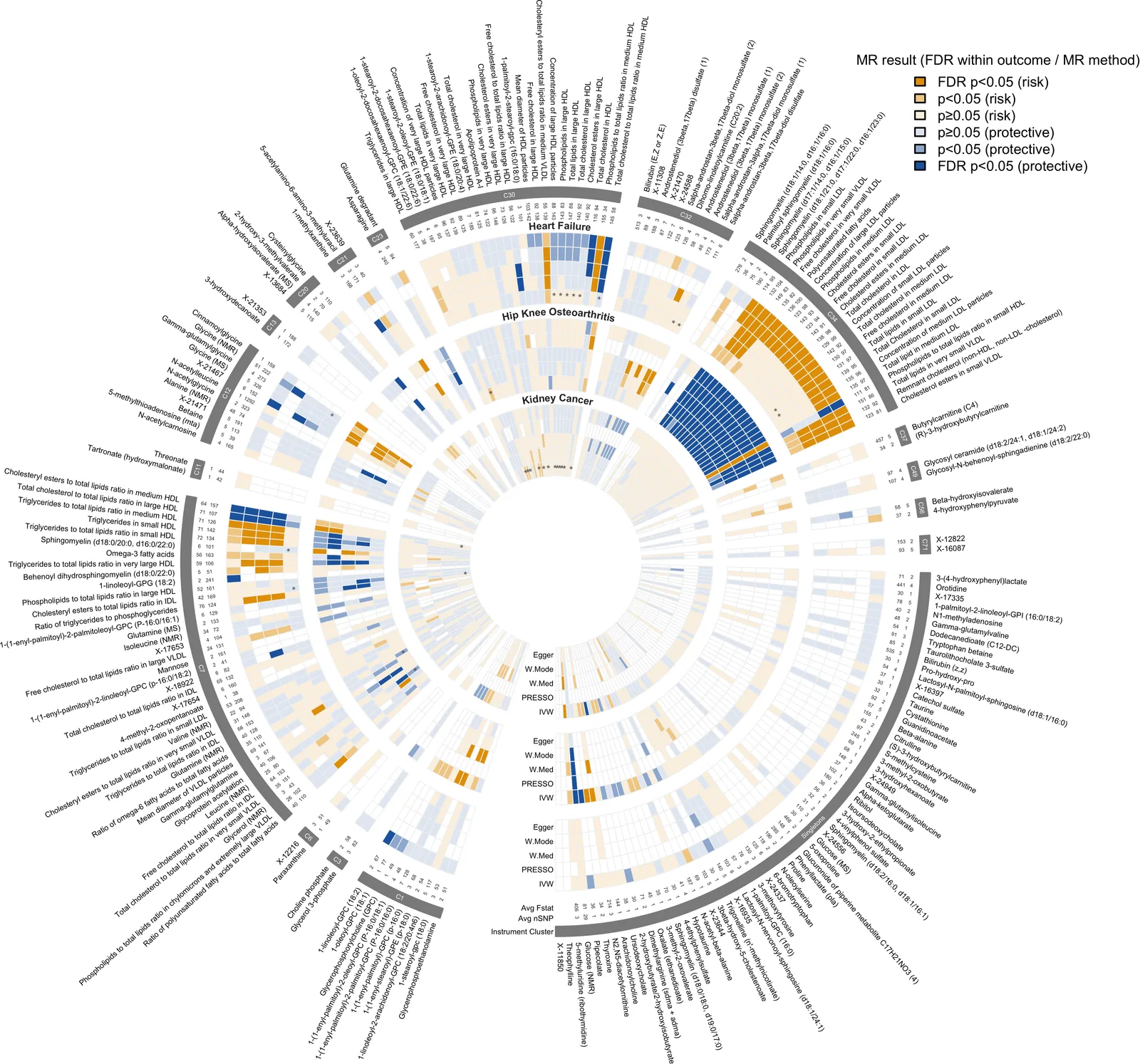
Mendelian randomisation estimates for the effect of metabolite levels on disease outcomes. Circos plot summarising mendelian randomisation results for metabolites across three outcomes: heart failure (outer), hip/knee osteoarthritis (middle), and kidney cancer (inner). Each outcome is represented by a heatmap ring with five MR methods presented per outcome: IVW, MR-PRESSO, Weighted Median, Weighted Mode, and MR-Egger. Cell colour indicates effect direction and strength of MR association: orange shades denote risk-increasing effects and blue shades denote protective effects, with full colour saturation indicating FDR-adjusted *p* < 0.05, half saturation indicating nominal *p* < 0.05, and pale colour indicating *p* ≥ 0.05. White cells indicate missing estimates. An asterisk (*) overlaid on MR-Egger cells denotes a significant Egger intercept (*p* < 0.05), indicating potential directional pleiotropy. Metabolites are grouped into sectors by genetic instrument cluster (outermost grey band; cluster labels shown within each band). The other two outer annotation tracks show the average number of SNPs (Avg nSNP) and average F-statistic (Avg Fstat) for each metabolite instrument across outcomes. FDR correction is applied within each outcome-method combination.

Several HDL traits were associated with decreased HF risk, including total cholesterol in HDL, total cholesterol to total lipids ratio in medium HDL, total cholesterol to total lipids ratio in large HDL, and cholesterol esters to total lipids ratio in medium HDL. Two medium HDL traits were associated with increased risk: phospholipids-to-total lipids ratio and triglycerides-to-total lipids ratio.

Sensitivity analyses, including MR-PRESSO, Steiger filtering and reverse MR, broadly confirmed the direction of effect from the primary IVW estimates (S10 Fig). Two metabolite traits showed evidence of bidirectional association: whilst higher total cholesterol-to-total lipids ratio in large HDL was protective for all-cause HF, genetic HF liability was also associated with a lower ratio. Conversely, phospholipids-to-total lipids ratio in medium HDL was positively associated in both directions. Although there was no absolute variant overlap between the HF instrument and these metabolite instruments (S7 Table), the weighted median and mode estimates in the reverse analyses were attenuated compared to the IVW estimates, warranting caution in interpreting these as genuinely reciprocal relationships rather than pleiotropy at shared lipid loci.

For the outcome of osteoarthritis, 34 metabolite traits demonstrated strong association in the IVW-MR analysis (Fig 4 and S6 Table). These were again predominantly VLDL and LDL lipoprotein measures from cluster 34, but the direction of effect was opposite to that observed in all-cause HF, with higher levels associated with decreased osteoarthritis risk.

Several lipid, carbohydrate and amino acid measures were also associated with osteoarthritis risk. Higher glycine, gamma-glutamylglycine, and cinnamoylglycine were associated with elevated risk—the variants used to instrument these metabolites were grouped into a distinct similarity cluster which included other glycine metabolite pathways. All sensitivity analyses for these measures supported the direction of effect (S11 Fig).

Only two metabolite traits demonstrated strong association with kidney cancer—5-methyluridine and ursodeoxycholate (Fig 4 and S6 Table). Higher 5-methyluridine was associated with increased risk (OR 1.12 per SD; 95% CI [1.06,1.19]; *p* < 0.001), instrumented by three variants, with direction of effect consistent across sensitivity analyses (S12 Fig). Higher ursodeoxycholate was associated with decreased risk, though this association was derived from a single variant, precluding formal sensitivity testing.

## Discussion

Integrating metabolomic data across dietary and surgical weight loss interventions with lifelong BMI exposure via MR, we identified a 242-metabolite signature consistently altered by intentional BMI reduction, the majority of which show genetic evidence for being driven by lifetime BMI. Differential components of this signature were associated with BMI-linked disease outcomes, implicating distinct metabolic pathways as putative mechanistic candidates in obesity-related disease risk.

Obesity is a major risk factor for the development of a broad spectrum of diseases, spanning cardiovascular conditions, mechanical complications, and numerous cancers [42]. Despite the recent advent of highly effective weight loss therapies rendering excess adiposity increasingly modifiable at both the patient and population level [43,44], the contributing molecular pathways mediating these disease risks remain poorly understood. Not all individuals are eligible for, respond to, or tolerate current weight-loss interventions, and it remains unclear whether therapeutic benefits are driven solely by adiposity reduction or by direct intervention-specific effects—a distinction with direct implications for clinical choice between an expanding repertoire of interventions [45]. In addition, identifying downstream candidate metabolic pathways may reveal novel therapeutic targets or biomarkers that could potentially be modulated independently of weight.

Individual analyses are susceptible to their own sources of biases with relation to identifying a metabolic signature of adiposity—intervention trials to trial-specific effects, observational data to reverse causation, and genetic approaches to pleiotropy. Here, we triangulated three complementary sources of evidence—dietary intervention, metabolic and bariatric surgery, and mendelian randomisation of lifetime BMI—each with distinct error structures, to identify metabolic signals robust to the limitations of any individual approach.

Around half of the measured metabolites show evidence for changes following either metabolic and bariatric surgery or dietary intervention, with lipids, lipoproteins and several amino acids the main responders. Well-established amino acid changes with metabolic and bariatric surgery were replicated, including reductions in branched-chain amino acids and increases in glycine [46]. Moderate correlation between the effects of the contrasting interventions suggests both shared and intervention-specific effects on the circulating metabolome. The majority of metabolites showing consistent effects across weight-loss interventions also showed consistent associations with BMI predicted using genetic data. This convergence provides a natural experiment in gene-environment equivalence, whereby genetic proxies and intervention-induced changes point toward the same underlying biology.

Our MR analysis of weight loss associated metabolites revealed distinct associations across three contrasting adiposity-linked diseases: HF, osteoarthritis, and kidney cancer. Lipid and lipoprotein metabolites dominated the HF associations, consistent with a case series enriched for ischaemic aetiology where nearly a third of cases had a prior history of myocardial infarction [37]. The detrimental VLDL and LDL cluster of signals were expected in this context, though the high genetic similarity across instruments for these individual lipoprotein sub-traits cautions against attributing effects to any particular measure in this cluster, reflecting instead a shared LDL/VLDL genetic architecture. Measures of HDL cholesterol enrichment were protective, whilst triglyceride and phospholipid enrichment of medium HDL, a marker of dysfunctional HDL remodelling [47], were associated with increased risk. For osteoarthritis, lipoprotein associations were broadly mirrored but in the opposing direction, with higher LDL and VLDL measures associated with decreased risk, something that has been described previously in observational and MR analyses [48]. This directional divergence across outcomes argues against a generic bias driving the lipoprotein findings and instead supports outcome-specific effects.

For kidney cancer, the metabolite signature of adiposity translated poorly into disease associations, with only two metabolites reaching FDR-adjusted significance: 5-methyluridine and ursodeoxycholate. This relative sparsity is itself informative, suggesting that the circulating metabolites most strongly perturbed by excess adiposity do not drive kidney cancer risk, are not effectively instrumented by genetics (potentially due to the lower sample size), or are not captured on the two metabolomic platforms used in this study. The two identified signals are nonetheless biologically interesting: 5-methyluridine implicates RNA modification pathways with relevance in tumourigenesis [49,50], whilst ursodeoxycholate, a bile acid with established anti-inflammatory and anti-proliferative properties [51], represents a potentially novel protective signal.

It is unlikely that all pathway perturbations associated with excess adiposity are relevant in the development of all adiposity-linked diseases. Attempting to define the pathways relevant to specific diseases is a worthwhile endeavour that may lead to condition-specific treatments outside simply inducing weight loss, though admittedly intentional weight loss brings many additional benefits. In addition, defining a reproducible metabolic signature of weight loss could have practical implications in managing and monitoring clinical trials. If a consistent metabolic signature can be defined and shown to be associated with improved clinical outcomes, it could serve as an intermediate biomarker in early-phase drug trials, helping accelerate the validation of new weight loss therapeutics.

There are several important limitations to the data presented. We bring together data from two different weight-loss interventions—the DiRECT trial enrolled individuals with obesity and type 2 diabetes whereas the By-Band-Sleeve trial enrolled a broader population of individuals with obesity. The dietary intervention trial was substantially smaller than the metabolic and bariatric surgery study with a consequent reduction in power. In our analysis, this will have resulted in some adiposity-related metabolites being filtered out due to apparent weak association. Incorporation of other weight-loss interventions, such as individuals treated with incretin-based therapies, as well as larger sample sizes, will aid in further confirming the metabolite signature of weight loss versus intervention-specific effects [45].

The Model 1 time point coefficient represents a within-group change following intervention rather than an identified intervention effect. There is no control group in the single-arm metabolic and bariatric surgery study, or the intervention-only analysis of the dietary intervention trial; this coefficient therefore cannot distinguish change caused by the intervention from secular trends, ageing over the follow-up period, or other time-varying influences. The use of a mixed-effects model does not alter this, as it determines only which observations contribute to the estimate. Only the time point-by-allocation interaction in the dietary intervention trial (Model 2) is protected against this by randomisation, and this interaction term was used for metabolite selection in that trial.

Instrumentation of individual metabolites is complicated by correlation between metabolites and by pleiotropy of their instruments. Clustering by instrument similarity revealed substantial variant sharing and metabolites within a cluster should not be interpreted as independent findings. Where several cluster members show concordant MR associations with an outcome, for example, LDL and VLDL traits in cluster 34 with all-cause HF, the most parsimonious interpretation is one signal detected multiple times through correlated measures of the same lipoprotein biology, rather than multiple distinct metabolites. However, associations were not uniformly consistent within all clusters and despite instruments sharing at least 25% of constituent variants, metabolites within the same cluster could demonstrate divergent or even opposing effects on disease outcomes depending on the nonshared component. Residual horizontal pleiotropy cannot be fully excluded despite multiple supporting sensitivity analyses, particularly for metabolites within shared instrument clusters. In this regard, our MR results should not be interpreted as evidence that any metabolite is directly causal for a disease outcome, but rather as evidence of potential pathway involvement and a basis for further investigation as to a metabolite’s specific role. A specific concern at the BMI-to-metabolite stage is that BMI-associated variants may influence metabolite levels through behavioural or lifestyle pathways not mediated by adiposity, violating the exclusion restriction assumption. Neither Steiger filtering nor convergence with the intervention data can exclude this, and triangulation across designs therefore reduces, but does not eliminate, uncertainty regarding instrument specificity. No formal mediation analysis was undertaken and these analyses do not estimate the proportion of the effect of BMI on disease risk acting through any metabolite.

Standard MR methods assume linear effects, and whilst threshold or saturation effects in metabolic pathways are biologically plausible, current nonlinear MR methods are insufficient to reliably model these potential relationships with genetic data [52]. Winner’s curse may attenuate metabolite MR estimates towards the null, producing conservative causal effect estimates; this concern is greatest for metabolites instrumented using the smaller MS GWAS (*n* = 8,299) and less for those from the larger NMR GWAS (*n* = 136,016). False discovery rate correction was applied within analytical strata rather than globally, which is less conservative; therefore, findings close to the significance threshold should be interpreted cautiously. All GWAS used in this analysis were conducted predominantly in European-ancestry populations and all adjusted for genetic principal components to mitigate within-study stratification. Residual population stratification cannot be excluded and findings may not generalise to nonEuropean populations. Replication in more ancestrally diverse cohorts will be needed to determine whether the identified metabolite signature of adiposity and its disease associations are broadly applicable.

Finally, the disease outcomes were selected to represent adiposity-linked conditions with contrasting mechanisms; however, each is itself a heterogenous entity, and metabolite associations relevant only to specific subtypes of disease may be obscured in analyses of the broader phenotype.

In conclusion, we have characterised a robust metabolic signature of adiposity across multiple independent sources of BMI variation. This integrative approach disentangles shared adiposity-related biological signals from intervention-specific and nonadiposity signals captured through BMI, thus prioritising putative mechanistic candidates for further investigation. Future work will refine this further through focus on comparisons with other weight-loss interventions and pursuing consistent signals to determine the potential to replicate the beneficial effects of weight loss for obesity-related diseases through pharmacological means.

## Supporting information

S1 Table GWAS data sources. The table presents the sources for external (pre-existing) GWAS summary statistics used in the analyses. In addition, overlapping cohorts between the Pulit and colleagues. BMI GWAS meta-analysis dataset (used to form the primary BMI genetic instrument) and the Karjalainen and colleagues NMR metabolite GWAS datasets are highlighted, alongside a worst-case sample overlap estimation.
(XLSX)

S2 Table Associations between metabolites and two weight-loss interventions: metabolic and bariatric surgery (BBS) and structured dietary intervention (DiRECT). Three models are reported, as described in the methods. Model 1 (BBS): bariatric surgery intervention effect (columns: bbs_*); a single-arm linear mixed-effects model where the estimate, standard error, and P-value relate to the time point term. The time point estimate reflects the within-group change in metabolite level following bariatric surgery. Model 1 (DiRECT): structured dietary intervention effect (columns: direct_*); the same single-arm model fitted in the DiRECT intervention arm only, where the time point estimate reflects the within-group change in metabolite level following a structured dietary intervention (calorie restriction). Model 2 (DiRECT): structured dietary intervention difference-in-differences (columns: direct_full_*); a two-arm model fitted in both DiRECT arms, including treatment allocation, time point, and their interaction. Two sets of estimates are reported: the time point main effect (direct_estimate_time, direct_se_time, direct_p_time) reflecting change over time in the control group, and the time point by allocation interaction (direct_estimate_interaction, direct_se_interaction, direct_p_interaction) reflecting the additional change in the intervention arm relative to controls—the primary statistic of interest for identifying intervention-driven metabolite changes. For all models, adjusted P-values are provided using the Benjamini–Hochberg FDR method (*_p_fdr). The total number of samples is given in *_n and the model formula in *_model. The metabolite platform is indicated in the platform column (Metabolon mass spectrometry, MS; Nightingale NMR).
(XLSX)

S3 Table Genetic instruments for body mass index (BMI). Independent genome-wide significant variants included as genetic instruments for BMI, derived from the meta-analysis of the GIANT consortium and UK Biobank GWASs (see S1 Table for source details). Variants were selected at *p* < 5x10^−8^ following LD clumping (r^2^ < 0.001, 10,000 kb window). Columns: variant rsID (rsid), chromosome (chr), base-pair position in GRCh37 coordinates (bp), effect allele (ea), other allele (oa), effect allele frequency (eaf), effect size in SD units of BMI per effect allele (beta) and its standard error (se), GWAS *p*-value (p), and sample size (n).
(XLSX)

S4 Table Mendelian randomisation estimates for the effect of life-long BMI exposure on metabolite levels. Each row presents one metabolite (label) and MR estimator (method) combination. Metabolites are annotated by their broad biochemical class (pathway_broad) and more specific biochemical subgroup (pathway_group) if known. The measurement platform is indicated (platform): either mass spectrometry (*metabolon*) or nuclear magnetic resonance spectroscopy (*nightingale*). The columns b, b_se, p, and p_fdr give the BMI-metabolite MR effect estimate (change in metabolite level per 1-SD increase in BMI), its standard error, nominal p-value, and FDR-corrected p-value, respectively. FDR correction was applied within each MR method stratum. Instrument diagnostics are provided as the number of SNPs in the harmonised instrument (nsnp), the mean F-statistic (fstat), and the number of SNPs retained after Steiger filtering (nsnp_steiger_filtered). The MR-Egger intercept test for directional pleiotropy is reported as the intercept estimate, standard error, and p-value (egger_intercept, egger_intercept_se, egger_intercept_p). Cochran’s Q statistic, degrees of freedom, and associated p-value (Q, Q_df, Q_pval) reflect instrument heterogeneity. The columns estimate_bbs and estimate_direct give the raw intervention effect estimates (change in metabolite level over the intervention period) from the By-Band-Sleeve metabolic and bariatric surgery trial and the DiRECT dietary intervention trial respectively; full details of these estimates including standard errors and *p*-values are provided in S2 Table. The column bmi_mr_agreement indicates concordance between the MR estimate direction and the weight-loss trial estimates, where a decrease in metabolite with weight loss would be expected to correspond to an increase with higher genetically predicted BMI; column values are: *agrees (fdr)*—concordant direction with MR FDR *p* < 0.05, *agrees (nominal)—*concordant direction with MR nominal *p* < 0.05, or, “*-*“—no concordance or insufficient evidence.
(XLSX)

S5 Table Metabolite genetic instrument overlap and clustering. Each row represents one metabolite. The GWAS Catalog accession identifier for the metabolite-level GWAS used to derive the genetic instrument is given in gwas_id, and the metabolite name in label. Pathway annotation is provided in pathway_group. The number of independent genome-wide significant variants comprising the genetic instrument for the metabolite is given in nsnp and the specific variant rsids as a comma-separated list in snps. Instrument overlap with other metabolites is described in overlapping_traits, which lists all metabolite instruments sharing at least one variant as a semicolon-separated list; each entry gives the GWAS accession, metabolite name, the percentage of the index metabolite’s variants shared, and the percentage of the overlapping metabolite’s variants shared—for example, GCST90199727|1-oleoyl-GPC (18:1)|100%|100% indicates that 100% of the index metabolite’s variants appear in 1-oleoyl-GPC (18:1)’s instrument and vice versa. The maximum pairwise overlap coefficient across all overlapping metabolites is given in max_overlap. Instrument cluster assignments derived from Louvain community detection are given in cluster_id. The column cluster_singleton indicates whether the metabolite was assigned to a cluster of size one (i.e., no instrument overlap with any other metabolite). The median pairwise Szymkiewicz-Simpson similarity coefficient within the assigned cluster, with IQR, is given in cluster_sim.
(XLSX)

S6 Table Metabolite-outcome mendelian randomisation results. Each row represents one metabolite (label) and MR estimator (method) combination for a given outcome (out_id). Metabolite annotations are provided as GWAS Catalog accession identifier (exp_id), pathway subgroup (pathway_group), and measurement platform (platform: *metabolon* mass spectrometry or *nightingale* NMR). Instrument cluster assignment from Louvain community detection is given in cluster_id, with cluster_singleton indicating whether the metabolite had no instrument overlap with any other metabolite. The mean instrument F-statistic is given in fstat. MR results are presented as odds ratio with 95% confidence interval (or) with nominal and FDR-corrected p-values (p, p_fdr). FDR correction was applied within each outcome-method stratum. Cochran’s Q statistic and associated *p*-value (Q, Q_pval) report instrument heterogeneity. The MR-Egger intercept estimate, standard error, and *p*-value (egger_intercept, egger_intercept_se, egger_intercept_p) provide a test for directional pleiotropy. MR-PRESSO results are provided where ≥3 instrument SNPs were available: the global heterogeneity test *p*-value (presso_global_p), number and proportion of outlier variants detected (n_outliers, prop_outliers), distortion test p-value (distortion_p), and a flag for whether an error prevented PRESSO estimation (presso_error). Steiger test results are given as a directional consistency flag (steiger_correct_dir; TRUE = direction of effect from exposure to outcome) and associated p-value (steiger_p), alongside the variance in the exposure and outcome explained by the instrument SNPs (exp_snp_r2, out_snp_r2).
(XLSX)

S7 Table Genetic instruments for outcomes used in outcome-metabolite reverse mendelian randomisation. Each row represents one independent genome-wide significant variant used as a genetic instrument for a given outcome (out_id). Columns rsid, chr, bp, ea, oa, beta, se, p, eaf, and n give the variant rsID, chromosome, base-pair position (GRCh37), effect allele, other allele, effect size estimate, standard error, *p*-value, effect allele frequency, and sample size, respectively, as reported in the source outcome GWAS. The number of weight-loss-consistent metabolite instruments that include this SNP is given in n_metab_overlap. The column max_metab_overlap gives the highest pairwise overlap for any single metabolite sharing this SNP, expressed as the number of shared variants and the percentage of the outcome and metabolite instruments they represent, respectively (format: n (x%|y%)). All metabolites whose instruments include this SNP are listed semicolon-separated in metab_overlap, with each entry being a concatenation of the metabolite name, GWAS Catalog accession, percentage of the outcome instrument, and percentage of the metabolite instrument represented by this SNP.
(XLSX)

S1 Fig By-Band-Sleeve sample and feature flow. Study flow diagram from the By-Band-Sleeve metabolic and bariatric surgery trial. The top panel depicts the single-arm trial design with all individuals undergoing bariatric surgery. The bottom panel shows the flow of individuals (N) and their samples through the quality control pipeline. Metabolites were measured on two profiling platforms—mass spectroscopy (MS) and nuclear magnetic resonance (NMR). The total number of samples available for analysis and the split between those available at baseline and the end of follow-up are shown. The number of features represents the number of individual metabolites available for analysis after quality control.
(TIFF)

S2 Fig DiRECT sample and feature flow. Study flow diagram from the DiRECT dietary intervention trial. The top panel depicts the randomised control trial design with individuals allocated to either the dietary intervention or control arms. The bottom panel shows the flow of individuals (N) and their samples through the quality control pipeline. Metabolites were measured on two profiling platforms—mass spectroscopy (MS) and nuclear magnetic resonance (NMR). The total number of samples available for analysis and the split between those available at baseline and the end of follow-up are shown. The number of features represents the number of individual metabolites available for analysis after quality control.
(TIFF)

S3 Fig Correlation of metabolite associations between weight loss interventions. The plot presents the unadjusted (time point = ’end’) estimates for the effect of a structured dietary programme (y-axis) against bariatric surgery (x-axis) on circulating metabolite levels. The top panel presents metabolites measured on the mass spectroscopy platform (MS) and the lower panel metabolites measured on the nuclear magnetic resonance platform (NMR) platform. The regression lines were fitted using Deming regression via the mcreg() function from the R package mcr. The error ratio was set to the ratio of mean squared standard errors averaged across all metabolites (mean(SE_BBS^2) / mean(SE_DiRECT^2)). Pearson correlation coefficients were tested against zero using a two-sided *t* test (n - 2 degrees of freedom), with 95% confidence intervals derived using Fisher’s z-transformation (cor.test() from the R package stats). Metabolites with the greatest orthogonal distance from the regression line are labelled.
(TIFF)

S4 Fig Interplatform common metabolite associations correlation. The scatter plot presents the effects for metabolites measured on both the mass spectroscopy platform (MS) and nuclear magnetic resonance platform (NMR) in each weight loss intervention study (blue, By-Band-Sleeve metabolic and bariatric surgery; orange, DiRECT structured dietary programme). The regression lines were fitted using Deming regression via the mcreg() function from the R package mcr. The error ratio was set to the ratio of mean squared standard errors averaged across all metabolites (mean(SE_MS^2) / mean(SE_NMR^)). Pearson correlation coefficients were tested against zero using a two-sided *t* test (n - 2 degrees of freedom), with 95% confidence intervals derived using Fisher’s z-transformation (cor.test() from the R package stats).
(TIFF)

S5 Fig Bland–Altman plot comparing intervention effect estimates between MS and NMR metabolite measurement platforms. The plot presents the results from a Bland-Altman analysis, comparing metabolite effect estimates from the NMR and MS platforms for 33 metabolites measured on both platforms. The mean difference (MS − NMR) was +0.01 (middle dashed line), with 95% limits of agreement of −0.29 to 0.32 (outer dashed lines). One metabolite (glutamine, 3% of those assessed) fell outside the limits of agreement, consistent with the expected 5% under the assumption of normally distributed differences, indicating high cross-platform agreement.
(TIFF)

S6 Fig Concordance of BMI-metabolite mendelian randomisation estimates derived using two alternative BMI instrument sets. A sensitivity analysis to assess how robust the BMI-metabolite two-sample MR associations are to the limited amount of sample overlap between the primary BMI exposure instrument and the NMR metabolite GWASs. Inverse variance weighted MR estimates for the effect of genetically proxied BMI on the 72 NMR metabolites that demonstrated consistent effects across both weight loss interventions are shown. The scatter plot compares associations derived using a BMI instrument from the GIANT and UK Biobank BMI meta-analysis (x-axis; *n* = 806,834, primary analysis) with instruments from the UK Biobank alone (y-axis; *n* = 361,194). The UK Biobank-only instrument shares no participants with the metabolite GWAS and so is free of sample overlap. Estimates are in SD units of metabolite per SD higher lifetime BMI. Error bars are 95% confidence intervals. The dashed line is the line of identity.
(TIFF)

S7 Fig Louvain cluster stability across overlap coefficient thresholds. Cluster stability (Adjusted Rand Index, ARI; black circles, left axis) and number of nonsingleton clusters (blue triangles, right axis) as a function of the Szymkiewicz–Simpson overlap coefficient threshold applied to metabolite genetic instrument SNP sets. ARI is calculated between cluster solutions at successive thresholds; values approaching 1 indicate high stability.
(TIFF)

S8 Fig Metabolite genetic instrument overlap network. Network graph of metabolite genetic instruments. Each node represents one metabolite GWAS instrument; edges connect pairs whose instruments share a Szymkiewicz–Simpson overlap coefficient ≥0.25 at the SNP level. Node colour indicates Louvain cluster membership; grey nodes are singletons (no edges above threshold).
(TIFF)

S9 Fig Composition of metabolite genetic instrument clusters. Bar chart showing the number of metabolites in each nonsingleton Louvain cluster (left panel) and the total number of singleton instruments (right panel), coloured by metabolite pathway group. Clusters are ordered by size (largest first). Annotated labels show up to five example metabolites per cluster.
(TIFF)

S10 Fig Bidirectional mendelian randomisation results: metabolites and heart failure. Circos plot summarising bidirectional mendelian randomisation results between circulating metabolites and heart failure. Metabolites are arranged around the circle and grouped into sectors by genetic instrument cluster (outermost grey band; cluster labels shown within each band, with unclustered metabolites grouped as ‘Singletons’). The outer heatmap ring (Metabolite to Heart Failure MR) displays results from six MR methods (IVW, IVW Steiger filtered, MR-PRESSO, Weighted Median, Weighted Mode, MR-Egger) for the forward direction (metabolite as exposure). Three annotation tracks between the cluster band and the forward MR ring show, for each metabolite: number of SNPs in the instrument (Fwd nSNP), instrument F-statistic (Fwd Fstat), and number of SNPs excluded by Steiger filtering (Steiger excl.). The inner heatmap ring (Heart Failure to Metabolite MR) displays equivalent results for the reverse direction (disease as exposure), with corresponding annotation tracks for instrument size, F-statistic, and Steiger-excluded SNPs. Cell colour indicates the direction and significance of the MR effect: orange shades denote risk-increasing effect and blue shades protective effect, with full colour saturation indicating FDR-adjusted *p* < 0.05, half saturation indicating nominal *p* < 0.05, and pale colour indicating *p* ≥ 0.05. White cells indicate missing estimates. An asterisk (*) overlaid on MR-Egger cells denotes a significant Egger intercept (*p* < 0.05), indicating potential directional pleiotropy. FDR correction is applied within each outcome-method combination.
(TIFF)

S11 Fig Bidirectional mendelian randomisation results: metabolites and hip-knee osteoarthritis. Circos plot summarising bidirectional mendelian randomisation results between circulating metabolites and hip-knee osteoarthritis. Metabolites are arranged around the circle and grouped into sectors by genetic instrument cluster (outermost grey band; cluster labels shown within each band, with unclustered metabolites grouped as ‘Singletons’). The outer heatmap ring (Metabolite to Hip Knee Osteoarthritis MR) displays results from six MR methods (IVW, IVW Steiger filtered, MR-PRESSO, Weighted Median, Weighted Mode, and MR-Egger) for the forward direction (metabolite as exposure). Three annotation tracks between the cluster band and the forward MR ring show, for each metabolite: number of SNPs in the instrument (Fwd nSNP), instrument F-statistic (Fwd Fstat), and number of SNPs excluded by Steiger filtering (Steiger excl.). The inner heatmap ring (Hip Knee Osteoarthritis to Metabolite MR) displays equivalent results for the reverse direction (disease as exposure), with corresponding annotation tracks for instrument size, F-statistic, and Steiger-excluded SNPs. Cell colour indicates the direction and significance of the MR effect: orange shades denote risk-increasing effect and blue shades protective effect, with full colour saturation indicating FDR-adjusted *p* < 0.05, half saturation indicating nominal *p* < 0.05, and pale colour indicating *p* ≥ 0.05. White cells indicate missing estimates. An asterisk (*) overlaid on MR-Egger cells denotes a significant Egger intercept (*p* < 0.05), indicating potential directional pleiotropy. FDR correction is applied within each outcome-method combination.
(TIFF)

S12 Fig Bidirectional mendelian randomisation results: metabolites and kidney cancer. Circos plot summarising bidirectional mendelian randomisation results between circulating metabolites and kidney cancer. Metabolites are arranged around the circle and grouped into sectors by genetic instrument cluster (outermost grey band; cluster labels shown within each band, with unclustered metabolites grouped as ‘Singletons’). The outer heatmap ring (Metabolite to Kidney Cancer MR) displays results from six MR methods (IVW, IVW Steiger filtered, MR-PRESSO, Weighted Median, Weighted Mode, MR-Egger) for the forward direction (metabolite as exposure). Three annotation tracks between the cluster band and the forward MR ring show, for each metabolite: number of SNPs in the instrument (Fwd nSNP), instrument F-statistic (Fwd Fstat), and number of SNPs excluded by Steiger filtering (Steiger excl.). The inner heatmap ring (Kidney Cancer to Metabolite MR) displays equivalent results for the reverse direction (disease as exposure), with corresponding annotation tracks for instrument size, F-statistic, and Steiger-excluded SNPs. Cell colour indicates the direction and significance of the MR effect: orange shades denote risk-increasing effect and blue shades protective effect, with full colour saturation indicating FDR-adjusted *p* < 0.05, half saturation indicating nominal *p* < 0.05, and pale colour indicating *p* ≥ 0.05. White cells indicate missing estimates. An asterisk (*) overlaid on MR-Egger cells denotes a significant Egger intercept (*p* < 0.05), indicating potential directional pleiotropy. FDR correction is applied within each outcome-method combination.
(TIFF)

S1 Checklist Reporting checklist for mendelian randomisation studies. Checklist from Skrivankova and colleagues. Strengthening the reporting of observational studies in epidemiology using mendelian randomisation (STROBE-MR): explanation and elaboration (BMJ 2021;375:n2233. https://doi.org/10.1136/bmj.n2233). This checklist is copyrighted by the Equator Network under the Creative Commons Attribution 3.0 Unported (CC BY 3.0) license.
(DOCX)

S1 File Trial registration for DiRECT, a primary care-based weight management intervention for type 2 diabetes remission. (PDF)

## Abbreviations

BMI: body mass index
DiRECT: Diabetes Remission Clinical Trial
ea: effect allele
eaf: effect allele frequency
FDR: false discovery rate
GIANT: Genetic Investigation of Anthropometric Traits
GWAS: genome-wide association study
HDL: high-density lipoprotein
HF: heart failure
IVW: inverse variance weighted
LDL: low-density lipoprotein
MR: mendelian randomisation
MS: mass spectroscopy platform
NHS: National Health Service
NMR: nuclear magnetic resonance
oa: other allele
OR: odds ratios
QC: quality control
RCT: randomised controlled trial
SD: standard deviation
SE: standard error
SNPs: single nucleotide polymorphisms.
