# Supplementary material for: A metabolomic signature of weight-loss and its associations with BMI-linked diseases: A post-hoc analysis of two intervention trials and a Mendelian randomisation study
Source: PLoS Med. 2026 Nov 4;23(11):e1005290. doi: 10.1371/journal.pmed.1005290 (PMC13766602; doi:10.1371/journal.pmed.1005290)
Supplement: Supplementary file 1 [file pmed.1005290.s021.pdf]

ISRCTN03267836 https://doi.org/10.1186/ISRCTN03267836

Diabetes Remission Clinical Trial

Submission date	Recruitment status	[X] Prospectively registered
04/12/2013	No longer recruiting	[X] Protocol
Registration date	Overall study status	[_] Statistical analysis plan
20/12/2013	Completed	[X] Results
Last Edited	Condition category	[_] Individual participant data
01/03/2024	Nutritional, Metabolic, Endocrine	

Plain English summary of protocol
Background and study aims
Substantial weight loss achieved following bariatric surgery (e.g. gastric band) can reverse type 2 diabetes (T2D) in 70-80% of patients. However, research has proved that it can also be reversed by a strict energy restricted diet with around 15 kg weight loss. The major clinical question is whether this new knowledge about T2D can now impact its management in GP surgeries. A new approach to routine weight management is clearly required. While previous guidelines have retained a 5-10% weight-loss target, the 2010 SIGN Obesity guideline has set a new weight loss /maintenance target of >15-20% for obese people with diabetes. In routine NHS diabetes care, few people achieve a weight loss of >15kg (or >15%). Although bariatric surgery is recommended for obese patients with diabetes, there is little realistic prospect of it being offered to most such patients due to limited resources. In addition, many patients will not agree to surgery. However, there is clear evidence that a combined medical programme of diet, exercise and anti-obesity drugs can generate and maintain >15 kg weight loss for many patients. The aim of the study is to find out whether it is possible to reverse diabetes and sustain this over 2 years, similar to the benefits that are achieved through sustained weight loss after bariatric surgery. Optimised weight management via a proven structured programme, Counterweight Plus, will be used in GP surgeries. The study will also find out about the mechanisms underlying reversal of diabetes and quantify quality of life and attitudes during the study.

Who can participate ?
Patients in participating GP practices who have been diagnosed with type 2 diabetes within the previous 6 years, and who fulfil all inclusion criteria, will be invited to participate in the study.

What does the study involve?
GP practices will be recruited and randomly allocated to either to deliver usual care or to deliver Counterweight Plus, which includes a total diet replacement phase (TDR) followed by structured food reintroduction and long-term weight loss maintenance. Patients recruited in each participating GP practice will receive the care to which their practice has been allocated. All participants will be followed up at 1 and 2 years.

What are the possible benefits and risks of participating?
For patients who are successful in maintaining >15 kg loss, it is expected that many will revert to a non-diabetic state, and remain so for at least 2 years. There will be multiple clinical and personal benefits from any degree of weight loss, which are expected as long as any weight loss

is maintained, for most patients and beyond the end of the study. There are no major risks from taking part in this study. The only significant risk from more intensive weight loss in the weight management programme arm is that gall-stones may become symptomatic. This is still relatively uncommon. This risk will be minimised by dietary provision which includes some fat consumption.

Where is the study run from?
The study is run from GP practices across Scotland and Tyneside, UK

When is the study starting and how long is it expected to run for?
The study will run for 5 years and recruitment will commence early in 2014

Who is funding the Study?
The study is funded by Diabetes UK

Who is the main contact?
Professor Mike Lean
Mike.Lean@glasgow.ac.uk

Contact information

Type(s)
Scientific

Contact name
Prof Mike Lean

Contact details
Professor of Human Nutrition
University of Glasgow
4th Floor Walton Building
Glasgow Royal Infirmary
84 Castle St
Glasgow
United Kingdom
G4 0SF
-
Mike.Lean@glasgow.ac.uk

Additional identifiers

Protocol serial number
GN13DI127

Study information

Scientific Title
Reversal of type 2 diabetes mellitus (T2DM) using non-surgical weight management with Low-Energy-Liquid-Diet and long-term maintenance, within routine NHS care

Acronym

DiRECT

Study objectives

Losing weight using a structured weight management programme which includes an initial period of total diet replacement, followed by carefully managed food reintroduction and then weight loss maintenance, is a viable treatment for putting T2DM into remission, and can be transferred to a larger scale as part of routine GP care, where large numbers of overweight people with T2DM are managed in the UK.

Ethics approval required

Old ethics approval format

Ethics approval(s)

West of Scotland Research Ethics Committee 3, 24/01/2014, REC ref: 13/WS/0314

Study design

Cluster randomized controlled design with GP practices as the unit of randomization

Primary study design

Interventional

Study type(s)

Treatment

Health condition(s) or problem(s) studied

Type 2 diabetes

Interventions

Practices randomised to the intervention will deliver Counterweight Plus, which includes a Total Diet Replacement phase followed by structured food reintroduction and long-term weight loss maintenance. Training for the practice nurses/dietitians in TDR delivery, maintenance diet programme and behaviour therapy will be provided by the Counterweight (CW) Specialists, using the training package developed for the Counterweight feasibility study in primary care.

Week 0-12: a commercial micronutrient-replete 825-853 kcal/d TDR (soups and shakes) will be provided to replace normal foods, with ample fluids, for 12 weeks. Participants will be seen for review weekly then every 2 weeks during this phase.
Week 12-18: stepped transition to food-based Weight Maintenance, replacing TDR with meals which contain 30% of energy from fat. During this phase participants will attend for review appointments every 2 weeks.
Weeks 18-104: participants will then be provided with an individually tailored calorie prescription to support weight stabilisation and prevent weight regain with monthly review appointments.

All subjects in the intervention arm who are physically capable will be advised about increasing daily physical activity. As an aid, patients will be recommended to obtain an inexpensive step-counter and to aim to reach and maintain their individual sustainable maximum.

Some patients find weight maintenance difficult, some relapse temporarily and gain weight rapidly. Others may tend to let things slip more gradually. If weight regains occurs in TDR randomised participants, or if diabetes is found to have returned (HbA1c risen above 6.5%) at

any time during the 18-month weight loss maintenance stage, rescue plans for weight gain prevention will be offered.

Tyneside patients will be invited to participate in studies to define the physiological mechanisms underlying the long-term reversal of T2DM. These participants will have magnetic resonance measurements of pancreas and liver fat, assessment of beta-cell function and very low density lipoprotein (VLDL) secretion rate, and indirect calorimeter measurement of whole body substrate oxidation.

A random sample of control and intervention patients will be invited to participate in semi-structured interviews focusing on experiences, barriers, facilitators and successful strategies for self-control.

Practices randomised to control will continue to deliver usual optimal diabetes and obesity management as per clinical guidelines.

Intervention Type
Behavioural

Primary outcome(s)
Reduction in weight of 15 kg (assumed equal on average to 15%) or more
Co-primary outcome: Reversal of diabetes (HbA1c <6.5%)
Both outcomes measured at 1 year

Key secondary outcome(s))
1. Quality of life measured by EQ-5D questionnaire
2. Physical activity level measured using Sensewear Monitor
3. Serum lipids
All outcomes measured at 1 year

Completion date
30/09/2018

Eligibility

Key inclusion criteria
1. Men and women aged 20-65 years, all ethnicities
2. T2DM of duration 0-6 years
3. Body Mass Index (BMI) >27 kg/m2

Participant type(s)
Patient

Healthy volunteers allowed
No

Age group
Adult

Lower age limit

20 years

Upper age limit
65 years

Sex
All

Total final enrolment
306

Key exclusion criteria
1. Current insulin use
2. HbA1c ≥12%
3. Substance abuse
4. Known cancer
5. Myocardial infarction within previous 6 months
6. Learning difficulties
7. Current treatment with anti-obesity drugs
8. Diagnosed eating disorder or purging
9. Pregnant/considering pregnancy
10. Patients who have required hospitalisation for depression or are on antipsychotic drugs

Date of first enrolment
01/01/2014

Date of final enrolment
05/08/2016

Locations

Countries of recruitment
United Kingdom

Scotland

Study participating centre
University of Glasgow
Glasgow
United Kingdom
G4 0SF

Sponsor information

Organisation

NHS Greater Glasgow and Clyde (UK)

ROR
https://ror.org/05kdz4d87

Funder(s)

Funder type
Charity

Funder Name
Diabetes UK (UK)

Alternative Name(s)
DIABETES UK LIMITED, British Diabetic Association

Funding Body Type
Private sector organisation

Funding Body Subtype
Trusts, charities, foundations (both public and private)

Location
United Kingdom

Funder Name
Cambridge Weight Plan (UK) - product and training support

Results and Publications

Individual participant data (IPD) sharing plan

IPD sharing plan summary
Not provided at time of registration

Study outputs

Output type	Details	Date created	Date added	Peer reviewed?	Patient-facing?
Results article	1-year results	10/02/2018		Yes	No
Results article	baseline results	01/03/2018		Yes	No
Results article	2-year results	01/05/2019		Yes	No

Results article		01/05/2021	01/06/2021	Yes	No
Results article	5-year results	26/02/2024	01/03/2024	Yes	No
Protocol article	protocol	16/02/2016		Yes	No
HRA research summary			28/06/2023	No	No
Participant information sheet	Participant information sheet	11/11/2025	11/11/2025	No	Yes
