# Supplementary material for: A metabolomic signature of weight-loss and its associations with BMI-linked diseases: A post-hoc analysis of two intervention trials and a Mendelian randomisation study
Source: PLoS Med. 2026 Nov 4;23(11):e1005290. doi: 10.1371/journal.pmed.1005290 (PMC13766602; doi:10.1371/journal.pmed.1005290)
Supplement: Supplementary file 2 [file pmed.1005290.s022.pdf]

PLOS Medicine reviewer responses: “*Metabolomic signature of weight loss and association with heart failure*”

New title: A weight-loss metabolomic signature shows divergent associations across BMI-linked diseases

Key

Grey text	- reviewer comments
Blue text	- author responses

Academic Editor

- This topic is interesting because it combines the assessment of possible mechanisms of weight loss interventions using different designs. The study appears straightforward and clear (although sometimes lack details). However, it is not very clear why only heart failure was considered given BMI causes multiple cardiovascular diseases.

Thank you for this comment. Our primary focus on heart failure was motivated by the strong epidemiological evidence linking obesity to HF risk across both HFpEF and HFrEF subtypes, the relative paucity of mechanistic and causal evidence for these associations and by the emerging clinical relevance of weight-loss interventions (GLP-1 receptor agonists) in HF populations. We acknowledge that BMI causally influences a broader range of conditions and that our HFpEF and HfrEF outcome GWAS datasets were underpowered compared to the all-cause HF GWAS, complicating interpretation.

Stepping back, the main message is one of consistency of metabolic association across different sources of variation in BMI, with the follow-on message that different components of this signature may act to differing extents amongst BMI-linked diseases. In light of this and the comments received, we felt it appropriate to open up analyses to examine contrasting BMI-linked conditions (all-cause HF (as before), hip/knee osteoarthritis and kidney cancer) which share BMI as a risk factor, but differ fundamentally in disease mechanism (mechanical loading and inflammatory pathways for osteoarthritis; hormonal and inflammatory pathways for kidney cancer). This expansion and the rationale for it has been added explicitly throughout the Introduction and Discussion sections.

- I also agree that the lack of adjustment for multiple comparison when assessing the relation with HF trait is problematic. From Figure 4, many signals (circle dots) would have disappeared if we apply a more stringent criteria.

Thank you. We agree that multiple testing adjustment is essential given the number of tests being performed. For the metabolite-outcome MR results, FDR correction is now applied within each outcome–method stratum (e.g. within all-cause HF × IVW, within all-cause HF × weighted median). This stratified approach avoids penalising associations in one outcome for the number of outcomes tested while still controlling the false discovery rate within each comparison. All results figures now distinguish three tiers of evidence: FDR-adjusted $p<0.05$ (full colour saturation), nominal $p<0.05$ (half saturation) and $p\geq0.05$ (pale). Primary conclusions are restricted to metabolites meeting the FDR-corrected threshold with consistent direction across ≥2 sensitivity estimators, which are now also clearly presented in the figures.

- The other issue is the metabolites themselves which are based on NMR and are highly correlated, and hence can introduce horizontal pleiotropy. This can be seen from the "protective effects" of HDL in HF trait. The use of multivariable MR could address these but will penalize the p values further.

Thank you, this is an important comment and picked up by several of the reviewers too. We now directly address the correlation structure of metabolite instruments through an analysis of metabolite genetic instrument overlap. We cluster metabolites together that share variants in order to assist in the interpretation of the effect estimates within clusters (identification of redundancy / pointers to the same underlying single).

Cluster membership is visualised in Supplementary Figure 7 (instrument overlap network graph) and Supplementary Figure 8 (cluster composition bar chart). Metabolites are grouped by instrument cluster

throughout the results figures, enabling readers to identify where apparent independent findings share genetic instruments. Whilst this clustering does not deal with the inherent pleiotropy with metabolite instruments, the MR-Egger intercept test and newly added MR-PRESSO test provide partial empirical assessment of directional pleiotropy within each instrument set.

Please ensure that the paper adheres to the PLOS Data Availability Policy (see http://journals.plos.org/plosmedicine/s/data-availability), which requires that all data underlying the study's findings be provided in a repository or as Supporting Information. For data residing with a third party, authors are required to provide instructions with contact information (web or email address) for obtaining the data. Please note that a study author cannot be the contact person for the data. PLOS journals do not allow statements supported by "data not shown" or "unpublished results." For such statements, authors must provide supporting data or cite public sources that include it.

The Data Availability statement has been updated with the following:

At the time the By-Band-Sleeve trial data were generated, participants were not asked for their permission to share data beyond the immediate project team. However, anonymised individual patient data from the By-Band-Sleeve trial will be made available upon request to the chief investigator (Jane Blazeby, J.M.Blazeby@bristol.ac.uk) for secondary research, conditional on assurance from the secondary researcher that the proposed use of the data is compliant with the Medical Research Council Policy on Data Sharing regarding scientific quality, ethical requirements and value for money and is compliant with the National Institute for Health and Care Research policy on data sharing. A minimum requirement with respect to scientific quality will be a publicly available prespecified protocol describing the purpose, methods and analysis of the secondary research (e.g. a protocol for a Cochrane systematic review), approved by a UK Research Ethics Committee or other similar, approved ethics review body. Participant identifiers will not be passed on to any third party.

The DiRECT trial data used for analysis are available on a research data repository (https://researchdata.gla.ac.uk/) with access given to researchers subject to appropriate data sharing agreements. Requests to access data should be made to the Principal Investigators of DiRECT via https://www.directclinicaltrial.org.uk/directstudyteam.html quoting project title 'What lies behind the causal impact of body mass index (BMI) level and change on human health? Added value from complementary study design and deep metabolic phenotyping'.

Currently your Data Availability statement does not adhere to our policy. When data is not legally allowed to be shared openly, please provide details on how researchers could ask for access. This needs to include contact details (email/URL), and the point of contact is not allowed to be the an author on the study. Please amend according to our policy.

See above.

We expect all researchers with submissions to PLOS in which author-generated code underpins the findings in the manuscript to make all author-generated code available without restrictions upon publication of the work. In cases where code is central to the manuscript, we may require the code to be made available as a condition of publication. Authors are responsible for ensuring that the code is reusable and well

documented. Please make any custom code available, either as part of your data deposition or as a supplementary file. Please add a sentence to your data availability statement regarding any code used in the study, e.g. "The code used in the analysis is available from Github [URL] and archived in Zenodo [DOI link]" Please review our guidelines at https://journals.plos.org/plosmedicine/s/materials-software-and-code-sharing and ensure that your code is shared in a way that follows best practice and facilitates reproducibility and reuse. Because Github depositions can be readily changed or deleted, we encourage you to make a permanent DOI'd copy (e.g. in Zenodo) and provide the URL.

We certainly can provide this and have added the following to the manuscript:

The code used to perform the analyses is available on GitHub: https://github.com/nicksunderland/bmi_metabolites_and_heart_failure and archived in Zenodo [DOI link to be confirmed after acceptance / confirmation that no further changes are required].

Thank you for providing your financial disclosure. Please also include URLs to the funders' websites.

The funders' websites have been included at the first reference of them in the Funding section of the manuscript.

Ethics statement: Please include the IRB approval number for the DiRECT study.

The Methods section has been amended with:

Ethics approval for DiRECT was granted by the West of Scotland Research Ethics Committee (reference number: 13/WS/0314)

FORMATTING - GENERAL

Abstract: Please structure your abstract using the PLOS Medicine headings (Background, Methods and Findings, Conclusions). Please combine the Methods and Findings sections into one section.

The abstract sections have been merged in the manuscript.

Abstract: Please include a statement on the main limitations of your study at the end of the 'Methods and Findings' section.

We have added to the abstract the following:

The main limitations of our study include the potential for residual horizontal pleiotropy with metabolite instruments despite multiple sensitivity analyses and the rank-normalised scale of metabolite measures, which limits direct physiological interpretability.

At this stage, we ask that you include a short, non-technical Author Summary of your research to make findings accessible to a wide audience that includes both scientists and non-scientists. The Author Summary should immediately follow the Abstract in your revised manuscript. This text is subject to editorial

change and should be distinct from the scientific abstract. Ideally each sub-heading should contain 2-3 single sentence, concise bullet points containing the most salient points from your study. In the final bullet point of 'What Do These Findings Mean?', please include the main limitations of the study in non-technical language. Please see our author guidelines for more information: https://journals.plos.org/plosmedicine/s/revising-your-manuscript#loc-author-summary.

An Author Summary has been added following the Abstract.

Please express the main results with 95% CIs as well as p values. When reporting p values please report as p<0.001 and where higher as the exact p value p=0.002, for example. Throughout, suggest reporting statistical information as follows to improve clarity for the reader "22% (95% CI [13%,28%]; p</=)". Please be sure to define all numerical values at first use.

All results reporting in the text and figures has been amended to the suggested format:

Xunits (95% CI [X,Y]; p</=Z)

Please include line numbers in the manuscript file. Use continuous line numbers (do not restart the numbering on each page).

Continuous line numbers have been added to the manuscript.

Please cite the reference numbers in square brackets. Citations should precede punctuation.

All in-text citations have been reformatted to numbered square brackets preceding punctuation throughout the manuscript.

FIGURES AND TABLES

Please define all abbreviations used in each figure/table (including those in Supporting Information files).

All figure and table legends have been updated.

Please consider avoiding the use of red and green in order to make your figure more accessible to those with color blindness.

Thank you for this suggestion. Our colour scheme uses blue (protective/negative effects) and red (risk-increasing/positive effects) rather than red and green. The three significance tiers are additionally distinguished by colour saturation (full, half and pale). Please let us know if these changes are acceptable.

SUPPLEMENTARY MATERIAL

Please note that supplementary material will be posted as supplied by the authors. Therefore, please amend it according to the relevant comments outlined here.

All supplementary Figures and Tables have been amended with a) legends defining all variables, and b) the suggested additional data-fields where requested (e.g. Q statistics and F statistics).

Please cite your Supporting Information as outlined here: https://journals.plos.org/plosmedicine/s/supporting-information

All supporting tables and figures references have been amended in line with the documentation.

REFERENCES
PLOS uses the numbered citation (citation-sequence) method and first six authors, et al.

The references have been amended to Vancouver style, with the first 6 authors.

Please ensure that journal name abbreviations match those found in the National Center for Biotechnology Information (NCBI) databases (http://www.ncbi.nlm.nih.gov/nlmcatalog/journals), and are appropriately formatted and capitalised.

The references have been amended in line with the documentation.

Where website addresses are cited, please include the complete URL and specify the date of access (e.g. [accessed: 12/06/2023]).

Websites references have been updated with the date of access.

Please also see https://journals.plos.org/plosmedicine/s/submission-guidelines#loc-references for further details on reference formatting.

The references have been amended in line with the documentation.

MENDELIAN RANDOMIZATION STUDIES

In the Introduction, please describe the exposure and the evidence for a potential causal relationship between exposure and outcome.

The potential relationship between adiposity-related metabolites and the three outcomes presented in the manuscript (heart failure, osteoarthritis and kidney cancer) is developed throughout the introduction. Whilst previous Mendelian randomization studies support a causal effect of higher BMI on heart failure, osteoarthritis and kidney cancer, the specific metabolic transducers mediating these effects remain poorly characterised – this is the gap we address.

In the Methods, please explicitly state the 3 core instrumental variable assumptions for the main analysis (relevance, independence, and exclusion restriction), as well assumptions for any additional or sensitivity analysis.

The methods section includes details of the 3 core assumptions:

The genetic instrument used in MR should satisfy three core instrumental variable assumptions: (i) relevance - each variant is robustly associated with BMI, confirmed through genome-wide significance and large F statistic (ideally F >> 10); (ii) independence - there are no measured or unmeasured confounders of the instrument and the outcome; and (iii) exclusion restriction - the variants affect outcomes only through BMI, not via alternative biological pathways.

In the Methods, please describe the MR estimator (e.g., 2-stage least squares, Wald ratio) and related statistics. Detail the included covariates and, in case of 2-sample MR, whether the same covariate set was used for adjustment in the 2 samples.

The MR estimators used are described in the Methods sections. Regarding the BMI-metabolite MR:

The primary analysis used an inverse variance weighted (IVW) estimator, representing the change in metabolite levels (in standard deviation units) per 1 SD increase in BMI. To assess robustness to pleiotropy, sensitivity analyses were conducted using MR-Egger, weighted median and weighted mode estimators. Additionally, estimators were run with Steiger filtering to exclude genetic variants that explain more variance in the metabolite than in BMI, as these are more likely to act primarily on the metabolite rather than through BMI, violating the exclusion restriction assumption.

…and regarding the metabolite-outcome MR:

Instrument selection, harmonisation, and sensitivity analyses followed the same procedure as described above, with IVW as the primary estimator or Wald ratio for only a single variant, using the TwoSampleMR R package.(32) Additionally, MR-PRESSO was applied to detect and correct for outlier variants driving heterogeneity in the IVW estimate, with the outlier-corrected estimate reported where the global heterogeneity test was significant.(33) Supporting evidence for the primary result was defined as ≥2 of 4 sensitivity estimators (weighted median, weighted mode, MR-Egger, MR-PRESSO) showing an effect estimate in the same direction at nominal P-value threshold < 0.05.

If you are presenting an instrumental variable estimate, please compare this to the conventional observational estimate.

Given the number of exposure-outcome associations presented and the lack of comprehensive observational estimates matching our outcomes, we have not presented observational estimates. Please see response to reviewer 1 (point 13).

Report the associations between genetic variant and exposure and between genetic variant and outcome, preferably on an interpretable scale.

The BMI and metabolite MR estimates represent lifelong exposure to a 1 SD higher BMI or metabolite level, respectively. This has been made explicit in the manuscript:

The primary analysis used an inverse variance weighted (IVW) estimator, representing the change in metabolite levels (in standard deviation units) per 1 SD increase in BMI.

MR estimates represent the change in log odds of the outcome per one standard deviation increase in lifetime metabolite exposure on the inverse rank normal transformed scale.

Report MR estimates of the relationship between exposure and outcome and the measures of uncertainty from the MR analysis, on an interpretable scale, such as odds ratio or relative risk per SD difference.

See above.

If relevant, please consider translating estimates of relative risk into absolute risk for a meaningful time period.

See above.

Please consider including plots to visualize results (e.g., forest plot, scatterplot of associations between genetic variants and outcome vs between genetic variants and exposure).

Given the number of exposures and outcomes considered, all MR scatter plots (>1000) have not been included. If the editorial team would like us to include these in a separate supplemental PDF we would be happy to produce this.

Reviewer 1

Major 1 - The statistical analysis section would benefit from a clearer separation between analyses of the intervention trials (dietary and surgical) and the Mendelian randomisation analyses. At present, the analytical narrative interleaves observational, interventional, and genetic analyses, which makes it difficult to follow the inferential framework and the role of each component in addressing the causal research question.

Thank you for this comment, we agree that addressing the weight loss trial evidence first, the presenting the BMI-metabolite MR as a separate line of evidence provides a more logical stepwise reading of the analyses. We have added a new overview figure (Figure 1), as well as changing the structure of the methods and results, to reflect this.

Major 2 - The manuscript implicitly treats metabolites as independent exposures, yet many metabolites—particularly within lipid and amino acid pathways—are highly correlated. The statistical analysis does not account for correlation structure among metabolites, either in instrument construction or in downstream inference. This raises concerns about redundancy of findings and inflation of apparent evidence across metabolite classes.

Thank you for this important comment. We have added a systematic analysis of genetic instrument overlap across all metabolite GWAS, as described in our response to the Academic Editor above. In brief, pairwise Szymkiewicz–Simpson overlap coefficients were computed between all instrument SNP sets and Louvain community detection was applied to identify clusters of metabolites sharing genetic architecture. This clustering is incorporated throughout all results figures, grouping metabolites by instrument cluster to make the correlation structure of instruments explicit to the reader. We note that the two dominant clusters identified were the LDL/VLDL lipoprotein cluster (cluster 34; median pairwise similarity 38%, IQR 25–63%) and an HDL cluster (cluster 30; median pairwise similarity 33%, IQR 25–48%), consistent with the known genetic architecture of lipoprotein metabolism. A further cluster of glycine and glycine-conjugated metabolites (cluster 12) was identified, consistent with the shared genetic regulation. Apparent independent findings within the same cluster are discussed cautiously throughout the Results and Discussion and we explicitly state that within-cluster results should not be interpreted as evidence of independent causal effects.

Major 3 - The definition of "BMI-metabolites" as those showing consistent effects across interventions and MR analyses is conceptually appealing but statistically circular. Because MR results are part of the selection criterion, this introduces a form of post-selection inference that may bias effect estimates and significance levels. This issue should be acknowledged and, if possible, mitigated (e.g., via split-sample or validation analyses).

Thank you. We note that the post-selection concern is mitigated in our design for two reasons. First, the BMI-metabolite MR and metabolite-outcome MR analyses use entirely distinct genetic instruments: BMI GWAS variants and metabolite GWAS variants respectively. Given this, statistical strength in stage 1 does not directly inflate instrument strength in stage 2. Second, to clarify this, we have changed the primary selection criterion to be trial consistency across the two independent weight-loss interventions. BMI-MR consistency is a secondary characterisation confirming adiposity-specificity rather than a hard filter applied before downstream MR. The metabolite–outcome MR analyses are therefore conducted on all weight-loss

metabolites with available instruments, with the BMI-MR consistency filter used to contextualise which associations have the greatest evidence across all threads and most likely to reflect adiposity biology.

Major 4 - The justification for the choice of MR as an inferential tool is conceptually sound; however, the Methods section does not sufficiently formalise the causal estimand. It is unclear whether the primary MR estimand is intended to represent a lifetime effect of metabolite levels on heart failure risk, or a shorter-term, modifiable exposure analogous to intervention-induced changes. This distinction is critical for interpretation and should be explicitly stated.

The methods section has been amended to read:

MR estimates represent the change in log odds of the outcome per one standard deviation increase in lifetime metabolite exposure.

We propose that the weight-loss trial data serve a complementary role - not to estimate intervention effects per se, but to identify metabolites plausibly downstream of adiposity, thereby anchoring the MR analysis in a biologically and clinically relevant exposure context.

Major 5 - Instrument selection for metabolite MR requires further methodological clarification. While genome-wide significant variants are mentioned, the manuscript does not fully describe the criteria for LD clumping (e.g., r^2 threshold, window size), nor whether conditional analyses were used for metabolites with dense genetic architecture. This omission limits reproducibility and makes it difficult to assess instrument validity.

The Methods now specifies all LD clumping parameters: r2<0.001, window size 10,000 kb, using a UK Biobank reference panel as the LD reference. Conditional analyses were not performed; instruments represent the lead independent SNP per LD block at genome-wide significance (p<5×10^-8). The variants used to instrument BMI, metabolites and the outcomes are provided in the supplementary tables.

The code used, which contains the parameters, will also be made available for full transparency.

Major 6 - The MR framework assumes linearity between genetically proxied metabolite levels and heart failure risk. No assessment of potential non-linear effects is reported, despite biological plausibility for threshold or saturation effects in metabolic pathways. The limitations of assuming linearity should be discussed in the Methods.

We have added a statement to the Limitations section:

Standard MR methods assume linear effects, and whilst threshold or saturation effects in metabolic pathways are biological plausible, current non-linear MR methods are unable to reliably model these potential relationships.

Major 7 - The handling of weak instruments is insufficiently addressed. Although multiple variants are used for several metabolites, there is no reporting of F-statistics, variance explained (R^2), or other diagnostics to

demonstrate instrument strength. Given the large number of metabolites analysed, weak instrument bias is a material concern and should be explicitly evaluated and reported.

Thank you, we have added F-statistics for each metabolite instrument to the supplementary tables and visualised these per metabolite as an annotation track in the metabolite MR circos plots. As a comment on metrics useful to consider performance of instruments in these analyses, all those included had F-statistic >10.

Major 8 - The manuscript acknowledges horizontal pleiotropy conceptually, but the statistical assessment of pleiotropy is not fully integrated into the main analysis. Sensitivity methods (MR-Egger, weighted median, weighted mode) are reported, but there is limited discussion of how discordant results across estimators were handled or how pleiotropy influenced inference. Pre-specifying decision rules for consistency across estimators would strengthen the analysis.

We have added to the Methods a pre-specified decision rule for interpreting sensitivity analysis consistency. Primary inference is based strictly on IVW at FDR $p<0.05$ within each outcome-method stratum. Supporting evidence is defined as ≥2 of 4 sensitivity estimators (weighted median, weighted mode, MR-Egger, MR-PRESSO) showing an effect estimate in the same direction as the primary IVW estimate. Cells where the MR-Egger intercept $p<0.05$ are flagged with an asterisk (*) in all circos visualisations, indicating potential directional pleiotropy. MR-PRESSO outlier-corrected estimates are presented alongside standard IVW for all metabolites with ≥3 instrument SNPs in the per-outcome circos plots and Supplementary Tables, allowing direct comparison.

Major 9 - The assumption of independence between genetic instruments and confounders is not empirically assessed. While this is common in two-sample MR, the availability of large GWAS summary statistics would allow for at least partial evaluation (e.g., via phenome-wide association scans or comparison of instrument associations with key confounders). The absence of such checks weakens causal claims.

This is a valid concern and an area on ongoing investigation; indeed, recent work by colleagues demonstrates widespread pleiotropy across all disease and molecular measure contexts, to varying extents (https://doi.org/10.64898/2026.02.19.26346618, https://gpmap.opengwas.io/). How this information is incorporated into MR analyses is an area of active development.

As highlighted, our methods conform to standard two sample MR methodology and we feel a formal phenome-wide assessment of metabolite instruments against potential confounders is beyond the scope of this already relatively complex combination of analyses. However, we address this concern in two ways. First, the use of Steiger filtering removes SNPs that explain more variance in the outcome than in the exposure, which would be inconsistent with the assumed causal direction and is a common source of instrument non-specificity or estimate misinterpretation. Second, the consistency of direction and magnitude across multiple sensitivity estimators – weighted median (requiring ≥50% of instrument weight from valid instruments), MR-Egger (testing for directional pleiotropy via the InSIDE assumption) and MR-PRESSO (outlier detection and correction) – each with different assumptions about the nature of pleiotropy, provides convergent evidence against the associations being entirely driven by pleiotropic pathways through confounders.

Major 10 - There is no explicit discussion of winner's curse or regression dilution bias in the GWAS used for instrument derivation. Given that some metabolite GWAS may have modest sample sizes relative to outcome GWAS, this could bias MR estimates towards the null or exaggerate associations.

We acknowledge that winner's curse in the metabolite could attenuate MR effect estimates towards the null when these variants are used as instruments. GWAS sample sizes for all metabolite instruments are provided in Supplementary Table 1. For metabolites with smaller discovery GWAS, attenuation towards the null is the expected direction of bias and would therefore lead to conservative rather than inflated MR estimates. We have added to the Limitations section:

Winner's curse may attenuate metabolite MR estimates towards the null, producing conservative causal effect estimates; this concern is greatest for metabolites instrumented using the smaller MS GWAS (n=8,299) and less for those from the larger NMR GWAS (n=136,016).

Major 11 - The manuscript does not address potential population stratification beyond reliance on original GWAS adjustments. Given the integration of multiple data sources, residual stratification could bias MR estimates and should be discussed explicitly in the Methods or limitations.

All GWAS used in this analysis adjusted for genetic principal components to control for population stratification within each study. We have added explicit acknowledgement of this in the Limitations section:

All GWAS used in this analysis were conducted predominantly in European-ancestry populations and all adjusted for genetic principal components to mitigate within-study stratification. Residual population stratification cannot be excluded and findings may not generalise to non-European populations. Replication in more ancestrally diverse cohorts will be needed to determine whether the identified metabolite signature of adiposity and its disease associations are broadly applicable.

Major 12 - Multiple testing is addressed using false discovery rate correction; however, the rationale for the chosen FDR threshold and its application across distinct analytical stages (intervention-metabolite associations, BMI-metabolite MR, metabolite-heart failure MR) is unclear. It should be clarified whether correction was applied globally or within analytical strata, and how this affects the interpretation of "consistent" metabolites.

Thank you for this comment, we have clarified the adjustments throughout the Methods and in the circos plot figure legends.

FDR correction is applied separately within each analytical stratum:

- within each weight loss trial
- within each method for the BMI-metabolite MR, and,
- within each metabolite-outcome MR method (e.g., within heart failure x IVW)

This stratified approach is more lenient than a global correction applied across all tests simultaneously and avoids penalising associations in one outcome for the number of outcomes tested.

Major 13 - The comparison between MR estimates and observational associations is potentially informative but methodologically underdeveloped. The manuscript does not clarify whether observational models were adjusted for the same covariate sets across datasets, nor how differences in scale and residual confounding were handled. Without this clarity, the comparison risks being descriptive rather than inferential.

We agree that direct inference from the comparison of MR and observational estimates was under-developed and sparse in places due to low overlap in the measured metabolites between this study and the observational HF subtype associations. In addition, there were indeed differences in covariate adjustment, outcome ascertainment and exposure measurement.

The addition of three distinct BMI-linked outcomes to this new version of the manuscript makes a full comparison of the MR results with observational estimates an unwieldy undertaking. We also do not have direct access to these types of data for the outcomes assessed. As such, we have removed all observational cohort comparisons.

Major 14 - The two-sample MR framework assumes no sample overlap between exposure and outcome GWAS. While the manuscript states that there is no overlap, no justification or supporting information is provided. Given the increasing reuse of large biobank datasets, this assumption should be explicitly justified or supported with references.

The metabolite GWAS used as exposure instruments do not include UK Biobank participants. We have added to Supplementary Table 1 quantifying the estimated proportion of UK Biobank cases in each GWAS source.

Major 15 - The Methods section does not describe any formal assessment of reverse causation (e.g., bidirectional MR). Given the biological plausibility that heart failure or preclinical disease processes could influence circulating metabolites, this represents an important omission.

Thank you for this important comment. We have implemented bidirectional MR analyses for each outcome, testing whether heart failure (and each contrast outcome) causally influences circulating metabolite levels. Genetic instruments for each outcome are described in Supplementary Table 7. Results from both forward (metabolite -> outcome) and reverse (outcome -> metabolite) MR are presented side-by-side in the per-outcome circos plots, with separate annotation tracks for instrument size (Rev nSNP), F-statistic (Rev Fstat), and Steiger-filtered SNP count for the reverse direction (Supplementary Figures 9-11).

Major 16 - The analysis combines heart failure subtypes (e.g., ischaemic vs non-ischaemic, preserved vs reduced ejection fraction) in some stages while stratifying in others. The rationale for when stratification is applied versus when pooled analyses are used is not clearly justified, which complicates interpretation of causal specificity.

The primary MR analyses use broadly defined all-cause heart failure given the largest available sample size. The previously presented subtype-specific analyses (HFpEF, HFrEF) were presented as secondary analyses but had substantially lower power. Given the move to examining three contrasting BMI-linked outcomes, these HF subtype analyses have been removed. The three outcomes, all-cause HF, osteoarthritis and kidney cancer, have more comparable sample sizes (Supplementary Table 1).

Major 17 - The manuscript does not clearly state whether harmonisation procedures accounted for strand ambiguity, palindromic SNPs, or allele frequency thresholds. Given the large number of instruments and outcomes, explicit documentation of harmonisation steps is essential for reproducibility.

Harmonisation was performed using the TwoSampleMR harmonise_data() function. Palindromic SNPs with minor allele frequency >0.42 were excluded; remaining palindromic SNPs were oriented using effect allele frequency matching. Non-palindromic SNPs were harmonised by effect allele alignment to the outcome GWAS. These steps have been added explicitly to the Methods.

Major 18 - The use of inverse-variance weighted (IVW) MR as the primary estimator assumes no directional pleiotropy. However, given the extensive overlap of genetic variants across metabolite instruments (as shown in supplementary figures), this assumption is likely violated. The rationale for retaining IVW as the primary analysis, rather than a pleiotropy-robust estimator, should be more strongly justified.

We retain IVW as the primary estimator because it is the most efficient estimator under the assumption of balanced or absent directional pleiotropy, is most widely used in the field facilitating comparison with prior work and provides the most complete metabolome coverage as some metabolites have too few instruments for reliable estimation with pleiotropy-robust methods such as weighted median, MR-Egger. The concern regarding pleiotropy is addressed through a pre-specified multi-estimator sensitivity framework: 1) MR-Egger intercept testing, flagged in all figures where intercept $p<0.05$; 2) MR-PRESSO outlier correction and global test for all metabolites with ≥3 SNPs; 3) Steiger filtering to remove directionally inconsistent SNPs; and 4) a pre-specified requirement for directional consistency across ≥2 of 4 sensitivity estimators for primary conclusions.

Major 19 - The manuscript does not report heterogeneity statistics (e.g., Cochran's Q) consistently alongside MR estimates, despite mentioning their calculation. Without systematic reporting, it is difficult to assess the reliability of pooled MR estimates across instruments.

Cochran's Q heterogeneity statistics and associated p-values are now reported systematically in Supplementary Tables 4 & 6.

Major 20 - The assumption of gene-environment equivalence is central to the paper's conceptual framework, yet the statistical analysis does not formally test whether genetic and intervention-induced metabolite changes are comparable in magnitude or direction beyond correlation plots. A more explicit quantitative comparison would strengthen this claim.

We acknowledge that formal testing of gene-environment equivalence is beyond the scope of this analysis. The comparison between genetically predicted BMI effects on metabolites and intervention-induced changes is presented as triangulated directional evidence rather than a claim of magnitude equivalence. The scatter plot of BMI MR estimates (IVW) versus trial effect estimates (bariatric surgery and dietary intervention) provide a visual assessment of concordance only, the legend of Figure 3 has been amended with:

A negative slope suggests directional concordance between weight-loss-induced and genetically predicted BMI effects on metabolites.

Minor 21 - The scaling of metabolite measures differs between platforms (rank-normalised SD for MS metabolites and raw SD for NMR metabolites). While this is described, the implications for comparability of effect sizes across platforms are not discussed and should be clarified.

We have added a statement to the Methods noting that rank-normalised effect sizes for MS-based metabolites and SD-scaled effects for NMR-based metabolites are not directly comparable in magnitude to the Results section:

As rank-normalised (MS) and z-score normalised (NMR) effect sizes are not directly comparable in magnitude, Bland-Altman analysis (mean difference 0.01; 95% limits of agreement −0.29 to 0.32) should be interpreted as reflecting consistency in the relative ordering and direction of effects across platforms rather than absolute magnitude equivalence (Supplemental Figures 3-5).

Minor 22 - The description of Deming regression used for cross-intervention and cross-platform comparisons is brief. Additional details on error variance specification and assumptions would improve transparency.

The primary cross-trial replication Figure 2 now compares BBS and DiRECT effect estimates against the line of identity, which is sufficient to assess directional consistency – the primary criterion for metabolite replication. For clarity, we have removed mention of Demming regression from the main manuscript.

Deming regression is still applied in supplementary correlation figures 3 & 4. The full technical details of the error variance specification are provided in the supplementary figure legends:

The regression lines were fitted using Deming regression via the mcreg() function from the R package mcr. The error ratio was set to the ratio of mean squared standard errors averaged across all metabolites (e.g. mean(SE_BBS^2) / mean(SE_DiRECT^2) for the cross-study comparison; mean(SE_MS^2) / mean(SE_NMR^2) for the cross-platform comparison). This pooled error ratio is a standard simplification for Deming regression when a single global error variance ratio is required. The assumption that random measurement error is present in both axes is justified using effect estimates derived from finite samples rather than true population values. Pearson correlation coefficients with 95% bootstrap confidence intervals are reported via the confintr package.

Minor 23 - Missing data handling is stated as "not applicable" for MR analyses, but this should be explicitly justified by clarifying that complete-case summary statistics were used throughout.

Yes, complete-case GWAS summary statistics were used in the two-sample MR analyses. We have updated MR instrument and harmonisation text in the Methods section clarify that SNPs absent from a given outcome GWAS were excluded / could not be incorporated into that analysis:

Exposure and outcome summary statistics were harmonised using the TwoSampleMR R package (action = 2), which infers positive-strand alleles for palindromic SNPs using allele frequencies, excluding those where the allele frequency is between 0.42 – 0.58 or variants that could not be harmonised.

Minor 24 - Confidence intervals are not consistently reported alongside point estimates in all MR-related tables and figures. Consistent reporting of uncertainty measures is recommended.

Reporting of estimates, confidence intervals and P-values has been standardised throughout as per the academic editor's comment above.

Minor 25 - The description of rank normalisation for MS metabolites lacks detail on whether this was performed within cohorts, across platforms, or globally, which has implications for interpretability of effect sizes.

We apologise as this was unclear. Normalisation was applied within each weight loss trial dataset separately. We have amended the Methods section accordingly:

MS datasets were transformed using a rank-based inverse normal transformation (INRT, where tied ranks were split by assigning random order) ***within trial datasets separately****. NMR datasets were transformed using Z-score normalisation* ***within trial datasets separately****, standardising each metabolite by subtracting the mean and dividing by the SD. Z-score normalisation was used as meaningful units can be preserved with NMR data whereas the MS data is semi-quantitative.*

Minor 26 - It is unclear whether the same genetic instruments were used across all heart failure outcomes or whether outcome-specific filtering was applied due to SNP availability. This should be clarified.

Genetic instruments were harmonised separately against each outcome GWAS and SNPs absent from a given outcome GWAS were excluded from that analysis. Consequently, the effective instrument size may differ across outcomes for the same metabolite. Per-metabolite instrument sizes for each outcome are shown in the annotation tracks of the per-outcome supplementary circos plots and reported in Supplementary Tables 4 & 6. The description of the instrument harmonisation process in the Methods section also details exclusion of non-matching variants, as described above.

Minor 27 - The Methods section would benefit from explicitly stating the minimum number of SNPs required to perform MR for a given metabolite and how metabolites with fewer instruments were handled.

For metabolites with a single instrument SNP, the Wald ratio was used; these were subsequently relabelled as IVW in results output for consistency in the figures, however the number of SNPs = 1 is presented alongside this.

Metabolites with no instrument SNPs available after harmonisation with a given outcome GWAS were excluded from that specific analysis and are represented as white (missing) cells in the circos heatmaps. No minimum SNP threshold was applied; single-SNP instruments are reported with appropriate caution given the inability to assess pleiotropy through multi-SNP sensitivity analyses.

Minor 28 - The approach to outlier detection (e.g., removal of influential SNPs) is not described. Methods such as MR-PRESSO or leave-one-out diagnostics are mentioned only briefly and could be better integrated into the analytical workflow.

Thank you for this comment. MR-PRESSO is run for all metabolite-outcome MR analyses using ≥3 instrument SNPs and its outlier-corrected estimates are presented as a dedicated column in the per-outcome circos plots and Supplementary Tables, allowing direct comparison with unadjusted IVW.

Minor 29 - The statistical analysis does not explicitly state whether all analyses were pre-specified or whether some were exploratory. This distinction is important given the breadth of analyses conducted.

The analysis was conducted as a largely hypothesis-generating study. The pre-specified analyses included:

1. determining replicating metabolites between weight loss trials
2. assessing concordance with life-time BMI exposure through MR, and,
3. metabolite-HF MR to assess the impact of concordant BMI-associated metabolites on HF and its subtypes as important BMI-linked disease outcomes.

Through this review process, several limitations in the power of the original HF subtype GWASs have been highlighted, as well as refinement of the message of components of the BMI metabolic profile being relevant to different extents in different disease contexts. This prompted the inclusion of three contrasting BMI-linked disease outcomes in this revision.

Minor 30 - The reporting of p-values without corresponding effect sizes in some supplementary materials reduces interpretability and should be avoided.

All supplementary tables now include effect sizes (beta or odds ratio) and 95% confidence intervals alongside p-values for all reported associations.

Minor 31 - The Methods section would benefit from a concise schematic or table summarising the full analytical pipeline, including data sources, estimands, statistical models, and sensitivity analyses

Thank you for this comment, we have amended Figure 1 to better represent the analytical pipeline which we believe significantly improves on the simplified overview in the original manuscript.

Minor 32 - The manuscript would benefit from clearer notation distinguishing observational regression coefficients, intervention effect estimates, and MR causal estimates to avoid confusion across sections.

The restructuring of the observational, BMI-MR and metabolite-outcome MR methods and results sections has hopefully clarified this and addressed this point. The removal of the observations estimates from previously published studies has also resulted in a clearer presentation of the data.

Reviewer 2

Major 1 - Clarity of the methodological framework.The manuscript lacks clarity regarding the core analytical strategy, and it takes considerable effort for the reader to understand what analyses were actually performed, what was the purpose and how the different components relate to each other.
It is often confusing, what is the main outcome variable and how is the exposure of interest defined. Also, the term "BMI-metabolites" is introduced without a sufficiently precise conceptual definition.

Thank you for this comment, we agree. After carefully considering the very helpful comments from this review, we have restructured the Methods under clearly labelled subsections corresponding to each analytical stage and added a new pipeline schematic / overview (Figure 1) to improve the communication of the work.

The term "BMI-metabolites" has been replaced throughout with "weight-loss-associated metabolites" to reflect that the primary selection criterion is consistency of effect across two weight-loss modalities; BMI-MR consistency is described as a secondary characterisation of these metabolites rather than a definitional requirement.

Major 2 - Causal interpretation and mediation claims. The authors frame the study as identifying mediators between weight loss and HF risk reduction. However, this interpretation is problematic.

- Causal mediation analysis generally relies on strong, untestable assumptions, which are not explicitly discussed.
- The analytical approach does not formally implement a mediation framework but instead combines association and MR analyses.
- Given issues such as pleiotropy and instrument non-specificity, the MR results should be interpreted cautiously and do not directly establish mediation.

Overall, the manuscript overstates the causal and mechanistic interpretation of the findings.

We agree that the language of mediation overstates what these analyses can formally establish. The manuscript has been revised throughout to frame metabolites as putative mechanistic candidates, identified through triangulation of evidence from intervention effects, genetic epidemiology and MR analyses, rather than as confirmed mediators. We acknowledge explicitly in the Discussion that the study the study does not provide definitive causal evidence for any individual metabolite in a specific disease context.

Major 3 - Insufficient description and transparency of statistical model. The statistical models are presented in a simplified form that ultimately reduces interpretability. For instance, the notation in Models 1, 2a, and 2b is unclear, as the outcome is indexed by i and t, but covariates on the right-hand side are not.

The statistical model notation has been revised and simplified to ensure consistent indexing of all variables. There are now only 2 models:

1. Addressing the intervention only arms of each trial (all of BBS and the intervention arm of DiRECT)
2. Incorporating the control arm of DiRECT

They are now appropriate indexed on both sides:

Model 1:

$$M_{i,t} \sim timepoint_t + age_i + sex_i + site_i + storage\ time_{i,t} + study\ specific\ covariates_i + (1|id_i)$$

Model 2:

$$M_{i,t} \sim timepoint_t + allocation_i + timepoint_t \cdot allocation_i + site_i + list\ size_i + age_i + sex_i + storage\ time_{i,t} + (1|id_i)$$

…and described in the text:

Model 1 was applied to each metabolite in the bariatric surgery cohort (a single-arm intervention study) and the intervention arm of the structured dietary intervention RCT. The normalised metabolite levels $M_{i,t}$ for individual i at timepoint t was regressed…

Major 4 - Identification of intervention effects vs. time trends. In Models 1 and 2b, it is unclear how the authors distinguish between time-related changes in metabolite levels (e.g., aging), and true intervention effects. This limitation should be more explicitly addressed and discussed.

We acknowledge that in the absence of a parallel control arm (BBS), Models 1 and 2 cannot formally distinguish intervention effects from natural metabolite changes over time. This is now stated explicitly as a limitation. For the DiRECT trial, Model 2 exploits the randomised control arm to estimate intervention effects as the interaction between time and randomisation group, which is the only model design that eliminates confounding by temporal trends. Results from Model 2 are therefore emphasised as the most causally interpretable intervention-effect estimates for DiRECT:

The primary statistic of interest in Model 2 was the timepoint-by-allocation interaction term and its associated P-value, identifying metabolites that changed differentially between treatment and control arms, confirming that observed changes in Model 1 were not solely attributable to the passage of time.

Major 5 - Choice of mixed-effects models with two timepoints. The use of linear mixed-effects models with only two timepoints raises questions. With baseline and follow-up only, modeling change scores would be equivalent and more transparent. The current formulation does not appear to provide additional benefit, while making interpretation more difficult. Importantly, this does not resolve the issue of separating intervention effects from temporal trends.

We retain the linear mixed-effects model formulation because it accommodates participants with missing follow-up measurements under a missing-at-random assumption, which a change-score approach would not. A complete data approach yields broadly equivalent estimates but with but with reduced precision. We have added a statement to the Methods confirming this equivalence and clarifying the rationale for preferring the LMM:

This approach was preferred over a simple change-scores, as not all participants had measurements at both timepoints. Retaining participants with incomplete pre-post data makes full use of all available observations and increases statistical power relative to a complete-cases analysis.

Major 6 - Limitations of Model 2a (RCT-based analysis). Model 2a, which estimates the effect of the dietary intervention via interaction terms, is conceptually appropriate and probably the only model that would identify the intervention effect that is unconfounded by other time trends. However, as the sample size is relatively modest and a large number of metabolites are tested, it does not seem plausible to detect robust signals can be reliably detected. The reviewer was also unable to locate the corresponding results in the supplementary materials.

We agree that Model 2 (previously Model 2a) is statistically underpowered given the DiRECT sample size and the large number of metabolites tested which impacts P-value adjustments. Results from Model 2 are now clearly presented in Supplementary Table 2. We acknowledge the power imbalance in the Limitations section:

The dietary intervention trial was substantially smaller than the bariatric surgery study with a consequent reduction in power. In our analysis this will have resulted in some adiposity-related metabolites being filtered out due to apparent weak association.

Major 7 - Ambiguity in Mendelian randomization design. The MR framework is not clearly described and raises several concerns, as it is unclear whether the primary exposures are BMI, metabolites, or both at different stages. Moreover, the use of genetic instruments for BMI to estimate effects on metabolites may be problematic, as BMI-associated variants may influence metabolites via behavioral or lifestyle pathways (e.g., diet), violating MR assumptions. These issues should be more clearly explained and critically discussed.

The comment, along with similar comments from the academic editor and other reviewers prompted a careful consideration of how the analyses are presented.

The Methods section has now been restructured to clearly distinguish two separate MR stages: 1) BMI -> metabolite MR, in which genome-wide significant BMI variants are used as instruments to estimate the effect of lifelong BMI on each metabolite, this is now presented as a characterisation of which metabolite changes are attributable to adiposity biology rather than as a primary causal analysis; and 2) metabolite -> outcome MR, in which metabolite-specific GWAS instruments are used to estimate causal effects on three contrasting disease outcomes.

The concern that BMI variants may act via behavioural pathways (diet, physical activity) is valid, however the argument presented is that three distinct sources of BMI variation converge on a set of metabolites robustly associated with adiposity. Bidirectional MR (outcome → metabolite) is included as a sensitivity analysis. Steiger filtering and MR-PRESSO are applied to mitigate instrument non-specificity in the metabolite-outcome MR.

Major 8 - Multiple testing and robustness of MR findings. The results of the MR analyses linking metabolites to HF outcomes appear weak, as most reported associations are borderline nominally significant. It is unclear how multiple testing correction was applied at this stage. Based on the presented results, only a very limited number of associations (e.g., total cholesterol in HDL particles) appear robust.

Thank you, this is an important point also raised by the academic editor (academic editor point 2).

As described above, we acknowledge that the number of metabolites meeting FDR-corrected significance for individual HF outcome is modest, and was minimal for the non-ischaemic HF sub-type presented previously. As described above, the HF subtypes have been removed.

Now, FDR correction is applied within each outcome-method stratum and three significance tiers are distinguished in all figures: FDR $p<0.05$ (full colour saturation), nominal $p<0.05$ (half saturation), and $p\geq0.05$ (pale). Primary conclusions are now restricted to associations meeting FDR-corrected significance with consistent direction across ≥2 sensitivity estimators.

Reviewer 3

Comment 1 - Use of UK Biobank in validation analyses: UK Biobank is a prominent contributor to the HERMES GWAS consortia used here for MR metabolite HF analyses. Although the directly measured metabolite study in UK Biobank is distinct from HERMES GWAS, the manuscript should acknowledge use of UKB (and presumably use of many of the same HF cases) in both the MR GWAS analysis and the validation analysis, and discuss the potential limitations in interpretation of findings thereof. This is particularly relevant given that the ARIC and UK Biobank directly measured metabolite analyses are appropriately described as exploratory post-hoc analyses.

Thank you for this comment. We confirm that the metabolomic GWAS datasets used as exposures do not include UK Biobank participants, and the observational comparisons previously using UK Biobank data have been removed from this revision. Please see the previous response (points 13 & 14, reviewer 1).

Comment 2 - HFpEF versus HFrEF subtyping and statistical power: The HERMES HF GWAS meta-analysis is dominated by broadly defined HF cases, with a relatively small proportion of participants classified into HFpEF or HFrEF subtypes (approximately 5% of total HF cases: ~5,406 HFrEF and ~3,841 HFpEF, derived from a non-ischemic subset). However, clear diagnostic distinction among HFpEF, HFmrEF, and HFrEF are central to guideline-directed therapy in clinical practice in US and EU and globally. As the authors note, HFpEF is more strongly linked to obesity, whereas evidence supporting weight-loss benefit in HFrEF remains limited. Emerging data from GLP-1 trials suggest benefits primarily in HFpEF rather than HFrEF. Given these considerations, the authors should more explicitly acknowledge the limited statistical power for subtype-specific inference. In addition, the authors may wish to comment on whether summary statistics from the Million Veteran Program HFpEF/HFrEF GWAS (doi: 10.1038/s41467-022-35323-0) were considered or could be incorporated to extend subtype-specific MR analyses.

We acknowledge the limited statistical power for the HF subtype-specific inference and have removed these as outcomes. Please see the previous comments regarding restructuring of the outcome analyses. Thank you for the suggestion regarding using the MVP data, we will pursue this in our continued work on HF phenotypes.

Comment 3 - Incident versus prevalent HF and clarity of outcome definitions: HERMES is largely a case-control GWAS meta-analysis of prevalent HF and should not be stated to be an incident HF GWAS. In contrast, ARIC and UK Biobank represent cohorts with incident HF ascertainment but are used here in exploratory post-hoc analyses. To avoid potential reader confusion, I recommend omitting the term "incident HF" from the abstract and explicitly mentioning in the abstract that the HERMES GWAS meta-analysis is the primary HF GWAS data source.

Thank you for this comment. All references to ‘incident HF’ have been removed and we have made clear in the Methods section the GWAS data sources used as outcomes, as well as updating the Supplementary Table 1 sources information:

Genome-wide association study data sources

Metabolite GWAS summary statistics were obtained from Karjalainen et al. and Chen et al, as described above.(28,29) Outcome GWAS summary statistics obtained from the HERMES consortium for all-cause

HF (cases/controls: 139,533/1,568,809);(34) Hatzikotoulas et al. for hip/knee osteoarthritis (213,839/1,080,482);(35) and Purdue et al. for kidney cancer (25,890/743,585).(36) Full details are provided in Supplemental Table 1.

Comment 4 - Contemporary clinical context: GLP1R therapies: The manuscript mentions GLP1R in just one sentence in the discussion (citing reference 50, no clinical trials are cited), despite the absolutely explosive and evolving clinical landscape in which GLP1 receptor agonist therapies are now widely used in cardiovascular therapeutics. Given the strong relevance of pharmacologic weight loss to HF prevention—particularly HFpEF, greater mention of this study in the context of GLP1 receptor therapies would strengthen both the Introduction and Discussion including greater discussion of relevance (or lack thereof?) to the metabolite findings. If the authors intend to address GLP-1-related metabolomic effects in a separate manuscript, this could be stated explicitly for transparency.

Thank you for this comment, it is indeed a gap in these analyses but one that we do not currently have the data access to address. We have highlighted the planned work in the text:

Incorporation of other weight-loss interventions, such as individuals treated with incretin-based therapies, as well as larger sample sizes, will aid in further confirming the metabolite signature of weight loss versus intervention-specific effects.

Comment 5. Additional limitations: Beyond the limited power for HF subtype analyses, additional limitations that merit possible mention are:
(i) residual horizontal pleiotropy of metabolite instruments despite sensitivity analyses;
(ii) the possibility that identified metabolites act as both mediators and biomarkers rather than direct causal agents; and
(iii) the use of rank-normalized metabolite measures, particularly for MS-based metabolites, which limits direct physiological and clinical interpretability.

Thank you for these suggestions. We have added the following to the Limitations section:

Residual horizontal pleiotropy cannot be fully excluded despite multiple supporting sensitivity analyses, particularly for metabolites within shared instrument clusters. In this regard our MR results should not be interpreted as evidence that any metabolite is directly causal for a disease outcome, rather as evidence of potential pathway involvement and a basis for further investigation as to a metabolite's specific role.

We have also clarified in the Methods section that metabolite MR estimates are on the inverse rank normalised scale:

MR estimates represent the change in log odds of the outcome per one standard deviation increase in genetically predicted lifetime metabolite exposure on the inverse rank normal transformed scale.
