# Supplementary material for: A metabolomic signature of weight-loss and its associations with BMI-linked diseases: A post-hoc analysis of two intervention trials and a Mendelian randomisation study
Source: PLoS Med. 2026 Nov 4;23(11):e1005290. doi: 10.1371/journal.pmed.1005290 (PMC13766602; doi:10.1371/journal.pmed.1005290)
Supplement: Supplementary file 3 [file pmed.1005290.s020.docx]

**STROBE-MR checklist of recommended items to address in reports of Mendelian randomization studies**^1^ ^2^

| **Item No.** | **Section** | **Checklist item** | **Section / Paragraph No. of section** | **Relevant text from manuscript** |
| --- | --- | --- | --- | --- |
| 1 | **TITLE and ABSTRACT** | Indicate Mendelian randomization (MR) as the study’s design in the title and/or the abstract if that is a main purpose of the study | Title  1 | Title: A metabolomic signature of weight-loss and its associations with BMI-linked diseases: an analysis of two intervention trials and a Mendelian randomization study |
|  | **INTRODUCTION** |  |  |  |
| 2 | **Background** | Explain the scientific background and rationale for the reported study. What is the exposure? Is a potential causal relationship between exposure and outcome plausible? Justify why MR is a helpful method to address the study question | Introduction  4 | Additionally, natural genetic variation in these intermediaries allows for the dissection of potential causal pathways through Mendelian randomization (MR) analyses. |
| 3 | **Objectives** | State specific objectives clearly, including pre-specified causal hypotheses (if any). State that MR is a method that, under specific assumptions, intends to estimate causal effects | Introduction  5  Discussion  10 | The aim of this study is to characterise the metabolic footprint of adiposity by leveraging separate sources of BMI variation to highlight consistent biology, and to determine which components of this signature are relevant to specific adiposity-linked diseases, using three mechanistically distinct outcomes – heart failure, osteoarthritis, and kidney cancer – as exemplars.  Residual horizontal pleiotropy cannot be fully excluded despite multiple supporting sensitivity analyses, particularly for metabolites within shared instrument clusters. In this regard our MR results should not be interpreted as evidence that any metabolite is directly causal for a disease outcome, rather as evidence of potential pathway involvement and a basis for further investigation as to a metabolite’s specific role. |
|  | **METHODS** |  |  |  |
| 4 | **Study design and data sources** | Present key elements of the study design early in the article. Consider including a table listing sources of data for all phases of the study. For each data source contributing to the analysis, describe the following: | Supp | S1 Table - appropriate references to the GWAS data sources provided |
|  | a) | Setting: Describe the study design and the underlying population, if possible. Describe the setting, locations, and relevant dates, including periods of recruitment, exposure, follow-up, and data collection, when available. | Supp | S1 Table - appropriate references to the GWAS data sources provided |
|  | b) | Participants: Give the eligibility criteria, and the sources and methods of selection of participants. Report the sample size, and whether any power or sample size calculations were carried out prior to the main analysis | Supp | S1 Table - appropriate references to the GWAS data sources provided |
|  | c) | Describe measurement, quality control and selection of genetic variants | Methods, BMI-metabolite Mendelian randomization  4 | Methods - Selection of instruments. |
|  | d) | For each exposure, outcome, and other relevant variables, describe methods of assessment and diagnostic criteria for diseases | Supp | S1 Table - appropriate references to the GWAS data sources provided. No new GWAS data created in this study. |
|  | e) | Provide details of ethics committee approval and participant informed consent, if relevant | Supp  Methods  3 & 4 | S1 Table - appropriate references to the GWAS data sources provided and ethics statements provided in these.  Ethics statement for the trials presented in this study provided in the Methods section, paragraphs 3 and 4. |
| 5 | **Assumptions** | Explicitly state the three core IV assumptions for the main analysis (relevance, independence and exclusion restriction) as well assumptions for any additional or sensitivity analysis | Methods, BMI-metabolite Mendelian randomization  5 | The genetic instrument used in MR should satisfy three core instrumental variable assumptions: (i) relevance - each variant is robustly associated with BMI, confirmed through genome-wide significance and large F statistic (ideally F >> 10); (ii) independence - there are no measured or unmeasured confounders of the instrument and the outcome; and (iii) exclusion restriction - the variants affect outcomes only through BMI, not via alternative biological pathways. |
| 6 | **Statistical methods: main analysis** | Describe statistical methods and statistics used |  |  |
|  | a) | Describe how quantitative variables were handled in the analyses (i.e., scale, units, model) | Methods, BMI-metabolite Mendelian randomization  6  Methods, Metabolite-outcome Mendelian randomization  1 | The primary analysis used an inverse variance weighted (IVW) estimator, representing the change in metabolite levels (in standard deviation units) per 1 SD increase in BMI.  MR estimates represent the change in log odds of the outcome per one standard deviation increase in lifetime metabolite exposure on the inverse rank normal transformed scale. |
|  | b) | Describe how genetic variants were handled in the analyses and, if applicable, how their weights were selected | Methods, BMI-metabolite Mendelian randomization  4 | Methods - Selection of instruments. |
|  | c) | Describe the MR estimator (e.g. two-stage least squares, Wald ratio) and related statistics. Detail the included covariates and, in case of two-sample MR, whether the same covariate set was used for adjustment in the two samples | Methods - Mendelian randomization analysis  2 | The primary analysis used an inverse variance weighted (IVW) estimator, representing the change in metabolite levels (in standard deviation units) per 1 SD increase in BMI. |
|  | d) | Explain how missing data were addressed | Methods, BMI-metabolite Mendelian randomization  4 | TwoSampleMR R package (action = 2), which infers positive-strand alleles for palindromic SNPs using allele frequencies, excluding those where the allele frequency is between 0.42 – 0.58 or variants that could not be harmonised. |
|  | e) | If applicable, indicate how multiple testing was addressed | Assessment of weight loss effect consistency between trials  1  Discussion  11 | Consistent effects were defined as metabolites demonstrating the same direction of association across interventions and those that reach a P-value threshold of < 0.05 after Benjamini & Hochberg false discovery rate (FDR) correction.  False discovery rate correction was applied within analytical strata rather than globally, which is less conservative, findings close to the significance threshold should be interpreted cautiously. |
| 7 | **Assessment of assumptions** | Describe any methods or prior knowledge used to assess the assumptions or justify their validity | Supp | S6 Table - MR sensitivity analysis results presented: Egger, weighted mode/median, Qstats |
| 8 | **Sensitivity analyses and additional analyses** | Describe any sensitivity analyses or additional analyses performed (e.g. comparison of effect estimates from different approaches, independent replication, bias analytic techniques, validation of instruments, simulations) | Mendelian randomization analysis  2 | To assess robustness to pleiotropy, sensitivity analyses were conducted using MR-Egger, weighted median and weighted mode estimators. Additionally, estimators were run with Steiger filtering to exclude genetic variants that explain more variance in the metabolite than in BMI, as these are more likely to act primarily on the metabolite rather than through BMI, violating the exclusion restriction assumption. |
| 9 | **Software and pre-registration** |  |  |  |
|  | a) | Name statistical software and package(s), including version and settings used | Mendelian randomization analysis  1 | TwoSampleMR R package |
|  | b) | State whether the study protocol and details were pre-registered (as well as when and where) | Study populations  2 & 3 | Trial references provided for the By-Band-Sleeve and DiRECT trials. |
|  | **RESULTS** |  |  |  |
| 10 | **Descriptive data** |  |  |  |
|  | a) | Report the numbers of individuals at each stage of included studies and reasons for exclusion. Consider use of a flow diagram | Supp | S1 Table - number of individuals included in the GWAS datasets. S1 and S2 Figures flow diagram of the weight loss trials. |
|  | b) | Report summary statistics for phenotypic exposure(s), outcome(s), and other relevant variables (e.g. means, SDs, proportions) | Supp | S1 Table - appropriate references to the GWAS data sources provided |
|  | c) | If the data sources include meta-analyses of previous studies, provide the assessments of heterogeneity across these studies | - | - |
|  | d) | For two-sample MR:  i.  Provide justification of the similarity of the genetic variant-exposure associations between the exposure and outcome samples  ii.  Provide information on the number of individuals who overlap between the exposure and outcome studies | Supp  BMI reduction drives trial metabolite variation: genetic evidence  4 | S1 Table - UK Biobank overlap between studies presented. Overlap between the GIANT+UKB BMI instrument and NMR metabolites presented and sensitivity analysis performed (almost perfect correlation:  To confirm that the residual sample overlap between the GIANT BMI GWAS and the NMR metabolite GWASs had not biased the 72 NMR metabolite estimates, we repeated the MR using instruments derived from UK Biobank alone, which share no participants with the NMR GWASs. The overlapping cohorts contribute only to the GIANT component of the BMI instrument, which represents a minority of the total sample. Estimates from the two instrument sets were near-identical (r = 0.996; S6 Figure), indicating the primary results are robust to this sample overlap. |
| 11 | **Main results** |  |  |  |
|  | a) | Report the associations between genetic variant and exposure, and between genetic variant and outcome, preferably on an interpretable scale | Supp | S6 Table |
|  | b) | Report MR estimates of the relationship between exposure and outcome, and the measures of uncertainty from the MR analysis, on an interpretable scale, such as odds ratio or relative risk per SD difference | Mendelian randomization analysis  1  Metabolite-outcome Mendelian randomization  1 | The primary analysis used an inverse variance weighted (IVW) estimator, representing the change in metabolite levels (in standard deviation units) per 1 SD increase in BMI.  MR estimates represent the change in log odds of the outcome per one standard deviation increase in lifetime metabolite exposure on the inverse rank normal transformed scale. |
|  | c) | If relevant, consider translating estimates of relative risk into absolute risk for a meaningful time period | - | See above |
|  | d) | Consider plots to visualize results (e.g. forest plot, scatterplot of associations between genetic variants and outcome versus between genetic variants and exposure) | - | Given the number of exposures and outcomes considered, all MR scatter plots (>1000) have not been included. If the editorial team would like us to include these in a separate supplemental PDF we would be happy to produce this. |
| 12 | **Assessment of assumptions** |  |  |  |
|  | a) | Report the assessment of the validity of the assumptions | Supp | S6/7 Table - MR sensitivity analysis results presented: Egger, weighted mode/median/PRESSO/Steiger filtered |
|  | b) | Report any additional statistics (e.g., assessments of heterogeneity across genetic variants, such as *I^2^*, Q statistic or E-value) | Supp | S6/7 Table - MR sensitivity analysis results presented: Qstats |
| 13 | **Sensitivity analyses and additional analyses** |  |  |  |
|  | a) | Report any sensitivity analyses to assess the robustness of the main results to violations of the assumptions | BMI reduction drives trial metabolite variation: genetic evidence  4 | Overlap of instruments with other metabolites discussed throughout in terms of clusters and presented on the MR circos plots in the main text and supplement  To confirm that the residual sample overlap between the GIANT BMI GWAS and the NMR metabolite GWASs had not biased the 72 NMR metabolite estimates, we repeated the MR using instruments derived from UK Biobank alone, which share no participants with the NMR GWASs. The overlapping cohorts contribute only to the GIANT component of the BMI instrument, which represents a minority of the total sample. Estimates from the two instrument sets were near-identical (r = 0.996; S6 Figure), indicating the primary results are robust to this sample overlap. |
|  | b) | Report results from other sensitivity analyses or additional analyses | - | - |
|  | c) | Report any assessment of direction of causal relationship (e.g., bidirectional MR) | S10-12Figs | Reverse MR results presented. |
|  | d) | When relevant, report and compare with estimates from non-MR analyses | - | - |
|  | e) | Consider additional plots to visualize results (e.g., leave-one-out analyses) | - | - |
|  | **DISCUSSION** |  |  |  |
| 14 | **Key results** | Summarize key results with reference to study objectives | Discussion  1 | First paragraph of the discussion |
| 15 | **Limitations** | Discuss limitations of the study, taking into account the validity of the IV assumptions, other sources of potential bias, and imprecision. Discuss both direction and magnitude of any potential bias and any efforts to address them | Discussion  10-11 | Limitations section. |
| 16 | **Interpretation** |  |  |  |
|  | a) | Meaning: Give a cautious overall interpretation of results in the context of their limitations and in comparison with other studies | Discussion  10-12 | Limitations section of the discussion. |
|  | b) | Mechanism: Discuss underlying biological mechanisms that could drive a potential causal relationship between the investigated exposure and the outcome, and whether the gene-environment equivalence assumption is reasonable. Use causal language carefully, clarifying that IV estimates may provide causal effects only under certain assumptions | Discussion  4 | This convergence provides a natural experiment in gene-environment equivalence, whereby genetic proxies and intervention-induced changes point toward the same underlying biology. |
|  | c) | Clinical relevance: Discuss whether the results have clinical or public policy relevance, and to what extent they inform effect sizes of possible interventions | Discussion  7 | Attempting to define the pathways relevant to specific diseases is a worthwhile endeavour that may lead to condition-specific treatments outside simply inducing weight loss, though admittedly intentional weight loss brings many additional benefits. In addition, defining a reproducible metabolic signature of weight loss could have practical implications in managing and monitoring clinical trials. If a consistent metabolic signature can be defined and shown to be associated with improved clinical outcomes, it could serve as an intermediate biomarker in early-phase drug trials, helping accelerate the validation of new weight loss therapeutics. |
| 17 | **Generalizability** | Discuss the generalizability of the study results (a) to other populations, (b) across other exposure periods/timings, and (c) across other levels of exposure | Discussion 7 | If a consistent metabolic signature can be defined and shown to be associated with improved clinical outcomes, it could serve as an intermediate biomarker in early-phase drug trials, helping accelerate the validation of new weight loss therapeutics. |
|  | **OTHER INFORMATION** |  |  |  |
| 18 | **Funding** | Describe sources of funding and the role of funders in the present study and, if applicable, sources of funding for the databases and original study or studies on which the present study is based | Funding | Funding section |
| 19 | **Data and data sharing** | Provide the data used to perform all analyses or report where and how the data can be accessed, and reference these sources in the article. Provide the statistical code needed to reproduce the results in the article, or report whether the code is publicly accessible and if so, where | Data availability | Data availability statement. |
| 20 | **Conflicts of Interest** | All authors should declare all potential conflicts of interest | Conflicts of interest | None |

This checklist is copyrighted by the Equator Network under the Creative Commons Attribution 3.0 Unported (CC BY 3.0) license.

1.	Skrivankova VW, Richmond RC, Woolf BAR, Yarmolinsky J, Davies NM, Swanson SA, et al. Strengthening the Reporting of Observational Studies in Epidemiology using Mendelian Randomization (STROBE-MR) Statement. JAMA. 2021;under review.

2.	Skrivankova VW, Richmond RC, Woolf BAR, Davies NM, Swanson SA, VanderWeele TJ, et al. Strengthening the Reporting of Observational Studies in Epidemiology using Mendelian Randomisation (STROBE-MR): Explanation and Elaboration. BMJ. 2021;375:n2233.
