# Supplementary material for: A metabolomic signature of weight-loss and its associations with BMI-linked diseases: A post-hoc analysis of two intervention trials and a Mendelian randomisation study
Source: PLoS Med. 2026 Nov 4;23(11):e1005290. doi: 10.1371/journal.pmed.1005290 (PMC13766602; doi:10.1371/journal.pmed.1005290)
Supplement: Supplementary file 5 [file pmed.1005290.s024.docx]

PLOS Medicine reviewer responses: “A weight-loss metabolomic signature shows divergent associations across BMI-linked diseases”

**New title:** A metabolomic signature of weight-loss and its associations with BMI-linked diseases: a post-hoc analysis of two intervention trials and a Mendelian randomization study

**Key**

Grey text	- reviewer comments

Blue text	- author responses

**Editor**

If you haven't already, we ask that you provide a short, non-technical Author Summary of your research to make findings accessible to a wide audience that includes both scientists and non-scientists. The Author Summary should immediately follow the Abstract in your revised manuscript. This text is subject to editorial change and should be distinct from the scientific abstract.

The author summary is provided after the abstract in the manuscript file.

Please modify your title to "A metabolomic signature of weight-loss and its associations with BMI-linked diseases: a post-hoc analysis of two intervention trials and a Mendelian randomization study".

Thank you, we have amended the title as suggested.

You don't need to provide the protocol as supplementary material. As we cannot include a published paper as supplementary material, please remove this file (PIIS2213858725000257.pdf) and instead cite the paper where necessary.

This has been removed. The original study is cited in the text.

Please remove the text "For the purpose of Open Access, the author has applied a CC BY public copyright license to any Author Accepted Manuscript version arising from this submission".  Although this is true, it does not need to be in the funding statement.

This has been removed.

Please ensure that the Zenodo DOI is included in the DAS (instead of the text “[DOI link to be confirmed after acceptance / confirmation that no further changes are required”]).

This has been updated to the Zenodo DOI: https://zenodo.org/records/23347488.

Please confirm that the data have been deposited in the controlled access repository, and amend your data availability statement accordingly. We do not accept the statement "The data used for analysis will be placed on a research data repository", as the data has to be available at the time of publication.

A data dictionary has been added to the Github and Zenodo repositories ([https://github.com/nicksunderland/weightloss_metabolomics/tree/main/output/datasets](https://github.com/nicksunderland/weightloss_metabolomics/tree/main/output/datasets%20s)) and the data availability statement has been amended to:

*Anonymised individual patient data from the By-Band-Sleeve trial are held in a controlled-access store at the University of Bristol Research Data Storage Facility (RDSF) and are available for secondary research on request to the study team (*[*by-band@bristol.ac.uk*](mailto:by-band@bristol.ac.uk%20k)*). Access is conditional on assurance from the secondary researcher that the proposed use complies with the Medical Research Council Policy on Data Sharing regarding scientific quality, ethical requirements, and value for money, and with the National Institute for Health and Care Research policy on data sharing. A minimum requirement with respect to scientific quality is a publicly available, prespecified protocol describing the purpose, methods, and analysis of the secondary research (e.g., a protocol for a Cochrane systematic review), approved by a UK Research Ethics Committee or other similar, approved ethics review body. Participant identifiers will not be passed on to any third party.*

*The terms of participant consent in DiRECT do not allow the study data to be made freely available in raw form. The DiRECT data used for this analysis are held in the University of Glasgow Trusted Research Environment, with access available to researchers subject to appropriate data sharing agreements (contact:* [*research-datamanagement@gla.ac.uk*](mailto:research-datamanagement@gla.ac.uk%20%20k)*).*

Please amend your Data Availability Statement to include that values underlying the graphs are present in the supplementary material.

This statement has been added to the Data Availability Statement.

**Reviewer 1:**

Thank you for thoroughly addressing all comments and providing detailed clarifications in the revised manuscript. I appreciate the effort you have put into enhancing the rigor and transparency of your analyses. The manuscript now reflects a robust and valuable contribution to subject, I am happy to recommend this work for publication and look forward to seeing its positive impact in the field.

We thank the reviewer for their time in reviewing this manuscript.
