# Supplementary material for: A metabolomic signature of weight-loss and its associations with BMI-linked diseases: A post-hoc analysis of two intervention trials and a Mendelian randomisation study
Source: PLoS Med. 2026 Nov 4;23(11):e1005290. doi: 10.1371/journal.pmed.1005290 (PMC13766602; doi:10.1371/journal.pmed.1005290)
Supplement: Supplementary file 4 [file pmed.1005290.s023.docx]

PLOS Medicine reviewer responses: “A weight-loss metabolomic signature shows divergent associations across BMI-linked diseases”

**New title:** A metabolomic signature of weight-loss and its associations with BMI-linked diseases: an analysis of two intervention trials and a Mendelian randomization study

**Key**

Grey text	- reviewer comments

Blue text	- author responses

**Editor**

- Please confirm that your title complies with PLOS Medicine's style. Your title must be nondeclarative and not a question. It should begin with main concept if possible. "Effect of" should be used only if causality can be inferred, i.e., for an RCT. Please place the study design ("A randomized controlled trial," "A retrospective study," "A modelling study," etc.) in the subtitle (ie, after a colon).

The paper title has been amended from:

*A weight-loss metabolomic signature shows divergent associations across BMI-linked diseases*

To:

*A weight-loss metabolomic signature and its associations with BMI-linked diseases: an analysis of two intervention trials and a Mendelian randomization study*

- Please confirm that your abstract complies with our requirements, including format (three sections: Background, Methods and Findings, and Conclusions) and providing all the information relevant to this study type <https://journals.plos.org/plosmedicine/s/submission-guidelines#loc-abstract>

We confirm that the abstract broadly complies with the provided guidance. We note that the abstract guidance requests that the study participants are described in terms of patient population and numbers of individuals included. This manuscript is focused on the metabolomics results from 3 data sources, the primary trials and GWAS summary statistics all themselves previously published, therefore we feel that detailed description of the trials in the abstract is too cumbersome and negatively impacts the communication of the study design. If the editorial team feel differently, we would be more than happy to find a compromise here.

- Please ensure that all abbreviations are defined at first use throughout the text.

All abbreviations have been defined at first use.

- Please confirm that all numbers presented in the abstract are present and identical to numbers presented in the main manuscript text.

We have checked that the figures in the abstract match the main manuscript:

- 242 metabolites were consistently altered across weight loss interventions

- Please include a statement regarding use or non-use of AI tools and technologies at the end of the Methods. Please specify the name(s) and version(s) of any tool(s) used and a description of their use, including how the AI-assisted or generated content was evaluated by authors, and a clear statement of which aspects of the study, article contents, data, or supporting files were affected or generated by AI tool usage. Please also include a statement specifying who retains responsibility for the final manuscript. See our guidelines for more information: <https://journals.plos.org/plosmedicine/s/ethical-publishing-practice#loc-artificial-intelligence-tools-and-technologies>.

The Claude code (Anthropic) coding assistant was used to help generate the code that produced the main structure of the circos plots (Figures 3 & 4). The data, labels and colours were added manually and the code iteratively (manually) refined to create the current figures. No image file was produced or edited by AI. We have added to the methods the following:

*AI tools and technologies statement*

*A large language model (Claude Code v2.1.263, Anthropic) was used to assist in generating the R code that produced the circos plots (Figures 3 & 4). R code utilising the circlize R package(41) was developed against variable names and summary-level structures; no individual-level participant data were provided to, or accessible by, the model. All code was read, executed line by line and output checked against the underlying data by the lead author. The authors take full responsibility for the content of the manuscript.*

- Funding statement: thank you for including this. Reading through it, it seems very extensive. Please consider limiting this section to only include funding for this specific study.

We have trimmed the funding as much as possible to the following:

*The funders had no role in study design, data collection and analysis, decision to publish, or preparation of the manuscript.*

*NSu is funded by the GW4-CAT (*[*https://gw4-cat.ac.uk/*](https://gw4-cat.ac.uk/)*) Wellcome PhD programme (316966/Z/24/Z). LP and NS work in a Medical Research Council (UKRI,* [*https://www.ukri.org/councils/mrc/*](https://www.ukri.org/councils/mrc/)*) funded unit (MC_UU_00032/1 and MC_UU_00032/3). MLS was supported by the Wellcome Trust through a PhD studentship (*[*https://wellcome.org/*](https://wellcome.org/)*, 218495/Z/19/Z).*

*By-Band-Sleeve was funded by National Institute of Health and Care Research (NIHR) Health Technology Assessment Programme (HTA 09/127/53). We also acknowledge funding from the MRC ConDuCT-II Hub for Trials Methodology Research and the NIHR Biomedical Research Centre (BRC) at the University of Bristol (IS-BRC-1215-20011). This trial was designed and delivered in collaboration with the Bristol Trials Centre, a UKCRC registered clinical trials unit (CTU), which is in receipt of NIHR CTU support funding. The views expressed are those of the author(s) and not necessarily those of the NIHR or the Department of Health and Social Care.*

*The DiRECT study was funded as a Strategic Research Initiative by Diabetes UK (*[*https://www.diabetes.org.uk/*](https://www.diabetes.org.uk/)*, award number 13/0004691). The formula diet was donated by Cambridge Weight Plan. Neither organisation had any input into the study design, data analysis or interpretation.*

*Metabolomics data generation in BBS and DiRECT was funded by a Wellcome Trust Investigator Award held by NJT (202802/Z/16/Z).*

*RTL is supported by the National Institute for Health Research University College London Hospitals Biomedical Research Centre (NIHR203328), Health Data Research UK (MR/S003754/1), British Heart Foundation Special Project Grant SP/F/24/150066, National Institutes of Health (5R01HL167509-02), and National Institute for Health and Care Research & the Medical Research Council Rare Disease Research UK Cardiovascular Initiative (MR/Y008235/1).*

*This research was funded in whole, or in part, by the Wellcome Trust [202802/Z/16/Z, 218495/Z/19/Z]. For the purpose of Open Access, the author has applied a CC BY public copyright licence to any Author Accepted Manuscript version arising from this submission.*

- DATA AVAILABILITY PLOS Medicine requires that the de-identified data underlying the specific results in a published article be made available, without restrictions on access, in a public repository or as Supporting Information at the time of article publication, provided it is legal and ethical to do so. Please see the policy at <http://journals.plos.org/plosmedicine/s/data-availability>
and FAQs at <http://journals.plos.org/plosmedicine/s/data-availability#loc-faqs-for-data-policy>. PLOS defines the “minimal data set” to consist of the data set used to reach the conclusions drawn in the manuscript with related metadata and methods, and any additional data required to replicate the reported study findings in their entirety. Authors do not need to submit their entire data set, or the raw data collected during an investigation. Please submit the following data:

- The values behind the means, standard deviations and other measures reported
- The values used to build graphs.

We believe we have found a solution to sharing the raw metabolite data used in this study. For the By-Band-Sleeve study we have created a minimal dataset (containing the variables used in this study) and plan to host on the data.bris Research Data Repository (<https://data.bris.ac.uk/data/>). For the DiRECT study we are in the process of creating a similar dataset to be hosted at Glasgow (<https://researchdata.gla.ac.uk/>). We need to conduct final checks surrounding deidentification but are confident that this can be achieved. Datasets would need to be requested via the trial contacts listed in the Data Availability statement:

*Anonymised individual patient data from the By-Band-Sleeve trial will be made available for secondary research upon request to the study (*[*by-band@bristol.ac.uk*](mailto:by-band@bristol.ac.uk)*), conditional on assurance from the secondary researcher that the proposed use of the data is compliant with the Medical Research Council Policy on Data Sharing regarding scientific quality, ethical requirements, and value for money, and is compliant with the National Institute for Health and Care Research policy on data sharing. A minimum requirement with respect to scientific quality will be a publicly available prespecified protocol describing the purpose, methods, and analysis of the secondary research (e.g., a protocol for a Cochrane systematic review), approved by a UK Research Ethics Committee or other similar, approved ethics review body. Participant identifiers will not be passed on to any third party.*

*The terms of participant consent in DiRECT do not allow making the study data freely available in its raw form. The data used for analysis will be placed on a research data repository (*[*research-datamanagement@gla.ac.uk*](mailto:research-datamanagement@gla.ac.uk)*;* [*https://researchdata.gla.ac.uk/*](https://researchdata.gla.ac.uk/)*) with access given to researchers subject to appropriate data sharing agreements.*

Regarding the main figures, the values are provided in the supplement:

- Figure 2 – values to recreate are presented in S2 Table
- Figure 3 – values to recreate are presented in S4 Table
- Figure 4 – values to recreate are presented in S6 Table

- The Data Availability Statement (DAS) requires revision. For each data source used in your study:

- If the data are freely or publicly available, note this and state the location of the data: within the paper, in Supporting Information files, or in a public repository (include the DOI or accession number).
- If the data are owned by a third party but freely available upon request, please note this and state the institutional owner of the data set and contact information for data requests (institutional web or email address). Note that a study author cannot be the contact for the data.
- If the data are not freely available, please describe briefly the ethical, legal, or contractual restriction that prevents you from sharing it. Please also include an appropriate institutional contact (institutional web or email address) for inquiries (again, this cannot be a study author).

We appreciate your statement that the data from both studies are not freely available, but please specify where the data can be requested, taking into account this cannot be a study author. Furthermore, please also include where the GWAS statistics used for the MR analysis can be found.

Regarding the summary statistics used, S1 Table now contains a column ‘source’ with a link to the respective download pages.

The ByBandSleeve data can be requested through the study administrative mailbox: [by-band@bristol.ac.uk](mailto:by-band@bristol.ac.uk) and access queries regarding the DiRECT data can be made via [research-datamanagement@gla.ac.uk](mailto:research-datamanagement@gla.ac.uk), as described in the above response.

- Thank you for including the code availability statement in your manuscript. Please also include this text in the metadata, as part of the data availability statement.

The code availability statement has been copied from the manuscript to the meta-data capture fields during submission.

- FIGURES: when a p value is given, please specify the statistical test used to determine it in the legend.

The tests used to determine the P-values are now provided. For example, S3 Figure legend:

*Pearson correlation coefficients were tested against zero using a two-sided t-test (n - 2 degrees of freedom), with 95% confidence intervals derived using Fisher's z-transformation (cor.test() from the R package stats).*

- FIGURES: Please consider avoiding the use of red and green in order to make your figure more accessible.

We have regenerated the figures using Okabe-Ito-like orange/blue palette.

- MENDELIAN RANDOMIZATON studies: please ensure that the study is reported according to the STROBE-MR guideline, and include the completed STROBE-MR checklist as Supporting Information.

The STROBE-MR guideline has been completed and amended as per the comments below.

- MENDELIAN RANDOMIZATON studies: please add the following statement, or similar, to the Methods: "This study is reported as per the Strengthening the Reporting of Observational Studies in Epidemiology (STROBE) guideline, specific for mendelian randomization (S1 Checklist).

We have added the statement:

*This study is reported as per the Strengthening the Reporting of Observational Studies in Epidemiology using Mendelian randomization (STROBE-MR) guideline (****S1 Checklist****).(27)*

- MENDELIAN RANDOMIZATON studies: thank you for including the STROBE-MR checklist. Please add a statement to the method citing this checklist (as above). Please also amend the checklist to use section and paragraph numbers, rather than page numbers.

We have updated the checklist with section names and paragraph numbers within each section.

**Reviewer 1:**

- The newly added clustering analysis based on shared genetic instruments improves interpretation of correlated metabolite signals. However, it does not fully address the potential for correlated metabolites within clusters to represent the same underlying biological signal. The limitations of interpreting clustered metabolites as independent findings could be acknowledged more explicitly in the Discussion.

Thank you, we have expanded the discussion on this point in the limitations section:

*Instrumentation of individual metabolites is complicated by correlation between metabolites and by pleiotropy of their instruments. Clustering by instrument similarity revealed substantial variant sharing and metabolites within a cluster should not be interpreted as independent findings. Where several cluster members show concordant MR associations with an outcome, for example LDL and VLDL traits in cluster 34 with all-cause HF, the most parsimonious interpretation is one signal detected multiple times through correlated measures of the same lipoprotein biology, rather than multiple distinct metabolites.*

- The authors state that there is no overlap between exposure and outcome GWAS datasets. It would be helpful to provide a more explicit justification or supporting references documenting the contributing cohorts to reassure readers that bias from sample overlap is unlikely.

We thank the reviewer for this comment, which prompted us to re-evaluate the contributing studies to the GIANT component of the BMI GWAS meta-analysis. In doing so we found that whilst our original statement was correct, no metabolite GWAS included UK Biobank participants, several studies contributing to the GIANT component of the BMI meta-analysis did also contribute to the NMR metabolite GWAS. We are grateful to the reviewer for prompting this check.

We have made three changes:

First, the S1 Table now lists the overlapping cohorts between the BMI and metabolite GWAS used, along with an estimate of the worst-case sample overlap.

Second, we have expanded the Methods to quantify the overlap:

*No metabolite GWAS included UK Biobank participants, so the UK Biobank component of the BMI instrument contributes no sample overlap. Several of the GIANT studies also contribute to the NMR metabolite GWAS. Assuming complete overlap within each shared cohort, at most 28,933 individuals were common to both samples, representing 4% of the BMI GWAS and 21% of the metabolite GWAS (S1 Table). Sample overlap can bias two-sample MR estimates toward the confounded observational association, although the bias is minimal when instrument strength is high. The GIANT meta-analysis was retained as the primary instrument given its greater power, however we performed a sensitivity analysis using BMI instruments derived from UK Biobank alone (n = 361,194, Neale Lab),(32) which share no participants with either metabolite GWAS.*

Third, we have added a sensitivity analysis to the results using BMI instruments derived from UK Biobank alone, which share no participants with either metabolite GWAS. As the shared cohorts contribute only to the GIANT component of the BMI instrument, a minority of the total sample, we expected minimal impact, and this was indeed the case:

*To confirm that the residual sample overlap between the GIANT BMI GWAS and the NMR metabolite GWASs had not biased the 72 NMR metabolite estimates, we repeated the MR using instruments derived from UK Biobank alone, which share no participants with the NMR GWASs. The overlapping cohorts contribute only to the GIANT component of the BMI instrument, which represents a minority of the total sample. Estimates from the two instrument sets were near-identical (r = 0.996; S6 Figure), indicating the primary results are robust to this sample overlap.*

- The rationale for applying FDR correction within analytical strata has been clarified and is reasonable. Nevertheless, the authors may wish to acknowledge that this approach is less conservative than a global correction across all analyses and could influence the interpretation of marginal findings.

We agree. Stratum-specific FDR correction is less conservative than a single global correction, and findings close to the significance threshold should therefore be interpreted with caution. We have added the following to the limitations section:

*False discovery rate correction was applied within analytical strata rather than globally, which is less conservative, findings close to the significance threshold should be interpreted cautiously.*

**Reviewer 1 comments on reviewer 2’s comments:**

- Causal and mechanistic interpretation - the language has been substantially moderated, particularly in the Discussion, where the authors explicitly state that the MR findings should not be interpreted as evidence that individual metabolites are directly causal. However, some wording still implies formal mediation or stronger causal inference than the analyses support. Most notably, the final box in Figure 1 states, “Distinct metabolite profiles mediate BMI’s effect on outcomes.” Similar language remains in places such as the Author Summary and the Discussion/conclusion, including references to metabolites “causally influencing” disease, “downstream metabolic mediators,” and pathways being isolated as downstream of adiposity and upstream of disease. I recommend replacing these remaining statements with wording such as “candidate pathways,” “putative mechanistic candidates,” or “metabolites associated with BMI-linked outcomes,” and explicitly stating that no formal mediation analysis was undertaken.

The text has been amended at the suggested remaining statements and other similar statements found during review. A summary of the changes is presented below.

| Before | After |
| --- | --- |
| Figure 1:  Distinct metabolite profiles mediate BMI’s effect on outcomes | Figure 1:  Candidate metabolic pathways linking BMI to specific outcomes |
| Differential components of this signature implicate distinct, disease-specific metabolic pathways as potential transducers of obesity-related disease risk, … | Differential components of this signature suggest distinct metabolic pathways linking excess adiposity and obesity-related disease risk, … |
| 242 circulating molecules (metabolites) consistently changed by weight loss, the majority of which showed evidence of being causally regulated by body weight. | 242 circulating molecules (metabolites) consistently changed by weight loss, the majority of which were supported by genetic evidence of being regulated by body weight. |
| Understanding which specific circulating molecules are altered by weight loss, and which of these are causally linked to disease… | Understanding which specific circulating molecules are altered by weight loss, and which of these associate with clinical outcomes… |
| We hypothesise that triangulating weight-loss metabolomics with MR-based disease analyses can identify disease-specific metabolic transducers of obesity, with implications for targeted prevention and treatment. | We hypothesise that triangulating weight-loss metabolomics with MR-based disease analyses can identify disease-specific obesity-related metabolic pathways, with implications for targeted prevention and treatment. |
| Weight loss associated metabolites were then instrumented using genetic variants and their causal effects on heart failure … | Weight loss associated metabolites were then instrumented using genetic variants and their associations with heart failure … |
| … supporting causal regulation by adiposity. | … consistent with regulation by adiposity |
| Using genetic methods to test whether these metabolites causally influence disease risk, we found … | Using genetic methods to test whether these metabolites are associated with disease risk, we found … |
| To test whether weight loss associated metabolites causally influence disease risk, … | To test whether weight loss associated metabolites influence disease risk, … |
| In addition, identifying downstream metabolic mediators may reveal novel therapeutic targets … | In addition, identifying downstream candidate metabolic pathways may reveal novel therapeutic targets … |
| …, thus isolating metabolic pathways likely to be downstream of adiposity and upstream of disease risk. | …, thus prioritising putative mechanistic candidates for further investigation. |
| Differential components of this signature were associated with BMI-linked disease outcomes, implicating distinct metabolic pathways as mechanistic candidates transducing obesity-related disease risk. | Differential components of this signature were associated with BMI-linked disease outcomes, implicating distinct metabolic pathways as putative mechanistic candidates in obesity-related disease risk. |
| …most strongly perturbed by excess adiposity do not transduce kidney cancer risk, | …most strongly perturbed by excess adiposity do not drive kidney cancer risk, |

In addition, we have added a statement regarding formal mediation analysis to the discussion:

*No formal mediation analysis was undertaken, and these analyses do not estimate the proportion of the effect of BMI on disease risk acting through any metabolite.*

- Intervention effects versus time trends - this is only partly addressed. Model 2 appropriately uses the timepoint-by-allocation interaction in DiRECT to estimate differential change between the randomized groups and therefore controls for temporal changes common to both groups. For the single-arm bariatric surgery analysis and intervention-only Model 1, however, the timepoint coefficient remains a within-group pre–post change and cannot distinguish an intervention effect from secular, ageing-related, or other time-varying changes. Although this is acknowledged in the response letter and can be inferred from the Methods, it is not stated explicitly in the Discussion limitations as claimed. I suggest adding an explicit sentence to the limitations and referring to the Model 1 coefficient as a “within-group change following intervention” rather than an identified intervention effect.

We agree that discussion of this point is useful for the reader. We have added to the limitations as suggested:

*The Model 1 timepoint coefficient represents a within-group change following intervention rather than an identified intervention effect. There is no control group in the single-arm metabolic and bariatric surgery study, or the intervention-only analysis of the dietary intervention trial, this coefficient therefore cannot distinguish change caused by the intervention from secular trends, ageing over the follow-up period, or other time-varying influences. The use of a mixed-effects model does not alter this, as it determines only which observations contribute to the estimate. Only the timepoint-by-allocation interaction in the dietary intervention trial (Model 2) is protected against this by randomisation, and this interaction term was used for metabolite selection in that trial.*

In addition, in the methods, we have amended the sentence:

*The timepoint coefficient estimates the mean change in metabolite following weight loss intervention.*

…to the following:

*The timepoint coefficient estimates the mean within-group change in metabolite following weight loss intervention.*

Whilst we have defined the coefficient as a within-group change at the two points where it is specified, the definition in the methods and its interpretation in the limitations, we have retained the shorter term ‘intervention effect’ in the results section, figures, and tables. We feel that substituting the full phrase at every mention may make it more difficult to read and digest. We hope that the proposed changes make the meaning of the coefficient unambiguous but would of course be happy to make the substitution more widely if the reviewer or editor prefers.

- Choice of mixed-effects models with two timepoints - the rationale for retaining the mixed-effects model is reasonable because it allows participants with incomplete repeated measurements to contribute information. Nevertheless, the manuscript should state that this approach relies on an appropriate missing-at-random assumption. It would also be helpful to acknowledge that, among participants with complete data, the estimate is broadly equivalent to a change-score analysis. The authors should make clear that using a mixed model does not itself resolve the temporal-trend limitation identified in comment 4.

We have amended the methods section to:

*Retaining participants with incomplete pre-post data makes full use of all available observations and increases statistical power relative to a complete-cases analysis but does rely on the assumption that missing observations are missing at random. With only two timepoints the model is broadly equivalent to a change-score analysis for participants with complete pre-post data.*

We have made temporal-trend limitation clear in the limitation section, as detailed in the above response.

- Mendelian randomization design - the structural ambiguity has been addressed: the manuscript now clearly separates BMI-to-metabolite MR from metabolite-to-outcome MR and identifies the exposure, outcome, instruments, and estimand at each stage. However, the specific concern about BMI-associated variants influencing metabolites through behavioural or lifestyle pathways that are not mediated by BMI has not been adequately discussed in the manuscript. The general description of MR assumptions and sensitivity analyses is helpful, but Steiger filtering and convergence across study designs cannot exclude this form of horizontal pleiotropy. Moreover, MR-PRESSO is described for the metabolite-to-outcome stage rather than as a solution to this concern in the BMI-to-metabolite stage. I recommend adding this as an explicit limitation and clarifying that triangulation reduces, but does not eliminate, uncertainty regarding instrument specificity.

We agree that this point should be surfaced in the discussion and have added the following:

*A specific concern at the BMI-to-metabolite stage is that BMI-associated variants may influence metabolite levels through behavioural or lifestyle pathways not mediated by adiposity, violating the exclusion restriction. Neither Steiger filtering nor convergence with the intervention data can exclude this, and triangulation across designs therefore reduces, but does not eliminate, uncertainty regarding instrument specificity.*

**Reviewer 3:**

This reviewer stated the manuscript can be accepted.

We thank reviewer 3 for their review of our revised manuscript.
