# Supplementary material for: A metabolomic signature of weight-loss and its associations with BMI-linked diseases: A post-hoc analysis of two intervention trials and a Mendelian randomisation study
Source: PLoS Med. 2026 Nov 4;23(11):e1005290. doi: 10.1371/journal.pmed.1005290 (PMC13766602; doi:10.1371/journal.pmed.1005290)
Supplement: Peer review history

# Editor report: Decision Letter 0

**DOI:** 10.1371/journal.pmed.1005290.r001

**Authors:** Suzanne De Bruijn

11 Dec 2025

Dear Dr SUNDERLAND,

Thank you for submitting your manuscript entitled "Metabolomic signature of weight loss and association with heart failure" for consideration by PLOS Medicine.

Your manuscript has now been evaluated by the PLOS Medicine editorial staff, and I am writing to let you know that we would like to send your submission out for external peer review. However, we will be looking for strong reviewer support.

Before we can send your manuscript to reviewers, we need you to complete your submission by providing the metadata that is required for full assessment. To this end, please login to Editorial Manager where you will find the paper in the 'Submissions Needing Revisions' folder on your homepage. Please click 'Revise Submission' from the Action Links and complete all additional questions in the submission questionnaire.

For clinical studies, please upload a copy of your trial study protocol as a supporting information file. The study protocol should be the version submitted for approval to the institutional review board or ethics committee, should include any amendments to the study protocol, as well as the date of their approval by the institutional review or ethics committee. Please also detail any deviations from the study protocol in the Methods section of your manuscript. The editors will consider the protocol and study conduct prior to a final decision for external review.

Please re-submit your manuscript within two working days, i.e. by Dec 15 2025 11:59PM.

Login to Editorial Manager here: https://www.editorialmanager.com/pmedicine

Once your full submission is complete, your paper will undergo a series of checks in preparation for peer review. Once your manuscript has passed all checks it will be sent out for review.

Feel free to email us at plosmedicine@plos.org if you have any queries relating to your submission.

Kind regards,

Suzanne De Bruijn, PhD

Associate Editor

PLOS Medicine

# Peer review: Decision Letter 1

**DOI:** 10.1371/journal.pmed.1005290.r002

**Authors:** Suzanne De Bruijn

30 Apr 2026

Dear Dr SUNDERLAND,

Many thanks for submitting your manuscript "Metabolomic signature of weight loss and association with heart failure" (PMEDICINE-D-25-04321R1) to PLOS Medicine. The paper has been reviewed by subject experts and a statistician; their comments are included below and can also be accessed here: [LINK]

As you will see, the reviewers think your study is of interest, but also have extensive concerns. After discussing the paper with the editorial team and an academic editor with relevant expertise, I'm pleased to invite you to revise the paper in response to the reviewers' comments. We plan to send the revised paper to some or all of the original reviewers, and we cannot provide any guarantees at this stage regarding publication.

Please also address all editorial comments, which you can find at the bottom of this email, and include these in your point-by-point response.

In addition to these revisions, you may need to complete some formatting changes, which you will receive in a follow up email. A member of our team will be in touch with a set of requests shortly. If you do not receive a separate email within a few days, please assume that checks have been completed, and no additional changes are required.

When you upload your revision, please include a point-by-point response that addresses all of the reviewer and editorial points, indicating the changes made in the manuscript and either an excerpt of the revised text or the location (eg: page and line number) where each change can be found. Please also be sure to check the general editorial comments at the end of this letter and include these in your point-by-point response. When you resubmit your paper, please include a clean version of the paper as the main article file and a version with changes tracked as a marked-up manuscript. It may also be helpful to check the guidelines for revised papers at http://journals.plos.org/plosmedicine/s/revising-your-manuscript for any that apply to your paper.

We ask that you submit your revision by May 21 2026 11:59PM. However, if this deadline is not feasible, please contact me by email, and we can discuss a suitable alternative.

Don't hesitate to contact me directly with any questions (sbruijn@plos.org).

Best regards,

Suzanne

Suzanne De Bruijn, PhD

Associate Editor

PLOS Medicine

sbruijn@plos.org

-----------------------------------------------------------

Comments from the academic editor:

- This topic is interesting because it combines the assessment of possible mechanisms of weight loss interventions using different designs. The study appears straightforward and clear (although sometimes lack details). However, it is not very clear why only heart failure was considered given BMI causes multiple cardiovascular diseases.

- I also agree that the lack of adjustment for multiple comparison when assessing the relation with HF trait is problematic. From Figure 4, many signals (circle dots) would have disappeared if we apply a more stringent criteria.

- The other issue is the metabolites themselves which are based on NMR and are highly correlated, and hence can introduce horizontal pleiotropy. This can be seen from the "protective effects" of HDL in HF trait. The use of multivariable MR could address these but will penalize the p values further.

-----------------------------------------------------------

Comments from the reviewers:

Reviewer #1: Major Comments

1) The statistical analysis section would benefit from a clearer separation between analyses of the intervention trials (dietary and surgical) and the Mendelian randomisation analyses. At present, the analytical narrative interleaves observational, interventional, and genetic analyses, which makes it difficult to follow the inferential framework and the role of each component in addressing the causal research question.

2) The manuscript implicitly treats metabolites as independent exposures, yet many metabolites—particularly within lipid and amino acid pathways—are highly correlated. The statistical analysis does not account for correlation structure among metabolites, either in instrument construction or in downstream inference. This raises concerns about redundancy of findings and inflation of apparent evidence across metabolite classes.

3) The definition of "BMI-metabolites" as those showing consistent effects across interventions and MR analyses is conceptually appealing but statistically circular. Because MR results are part of the selection criterion, this introduces a form of post-selection inference that may bias effect estimates and significance levels. This issue should be acknowledged and, if possible, mitigated (e.g., via split-sample or validation analyses).

4) The justification for the choice of MR as an inferential tool is conceptually sound; however, the Methods section does not sufficiently formalise the causal estimand. It is unclear whether the primary MR estimand is intended to represent a lifetime effect of metabolite levels on heart failure risk, or a shorter-term, modifiable exposure analogous to intervention-induced changes. This distinction is critical for interpretation and should be explicitly stated.

5) Instrument selection for metabolite MR requires further methodological clarification. While genome-wide significant variants are mentioned, the manuscript does not fully describe the criteria for LD clumping (e.g., r² threshold, window size), nor whether conditional analyses were used for metabolites with dense genetic architecture. This omission limits reproducibility and makes it difficult to assess instrument validity.

6) The MR framework assumes linearity between genetically proxied metabolite levels and heart failure risk. No assessment of potential non-linear effects is reported, despite biological plausibility for threshold or saturation effects in metabolic pathways. The limitations of assuming linearity should be discussed in the Methods.

7) The handling of weak instruments is insufficiently addressed. Although multiple variants are used for several metabolites, there is no reporting of F-statistics, variance explained (R²), or other diagnostics to demonstrate instrument strength. Given the large number of metabolites analysed, weak instrument bias is a material concern and should be explicitly evaluated and reported.

8) The manuscript acknowledges horizontal pleiotropy conceptually, but the statistical assessment of pleiotropy is not fully integrated into the main analysis. Sensitivity methods (MR-Egger, weighted median, weighted mode) are reported, but there is limited discussion of how discordant results across estimators were handled or how pleiotropy influenced inference. Pre-specifying decision rules for consistency across estimators would strengthen the analysis.

9) The assumption of independence between genetic instruments and confounders is not empirically assessed. While this is common in two-sample MR, the availability of large GWAS summary statistics would allow for at least partial evaluation (e.g., via phenome-wide association scans or comparison of instrument associations with key confounders). The absence of such checks weakens causal claims.

10) There is no explicit discussion of winner's curse or regression dilution bias in the GWAS used for instrument derivation. Given that some metabolite GWAS may have modest sample sizes relative to outcome GWAS, this could bias MR estimates towards the null or exaggerate associations.

11) The manuscript does not address potential population stratification beyond reliance on original GWAS adjustments. Given the integration of multiple data sources, residual stratification could bias MR estimates and should be discussed explicitly in the Methods or limitations.

12) Multiple testing is addressed using false discovery rate correction; however, the rationale for the chosen FDR threshold and its application across distinct analytical stages (intervention-metabolite associations, BMI-metabolite MR, metabolite-heart failure MR) is unclear. It should be clarified whether correction was applied globally or within analytical strata, and how this affects the interpretation of "consistent" metabolites.

13) The comparison between MR estimates and observational associations is potentially informative but methodologically underdeveloped. The manuscript does not clarify whether observational models were adjusted for the same covariate sets across datasets, nor how differences in scale and residual confounding were handled. Without this clarity, the comparison risks being descriptive rather than inferential.

14) The two-sample MR framework assumes no sample overlap between exposure and outcome GWAS. While the manuscript states that there is no overlap, no justification or supporting information is provided. Given the increasing reuse of large biobank datasets, this assumption should be explicitly justified or supported with references.

15) The Methods section does not describe any formal assessment of reverse causation (e.g., bidirectional MR). Given the biological plausibility that heart failure or preclinical disease processes could influence circulating metabolites, this represents an important omission.

16) The analysis combines heart failure subtypes (e.g., ischaemic vs non-ischaemic, preserved vs reduced ejection fraction) in some stages while stratifying in others. The rationale for when stratification is applied versus when pooled analyses are used is not clearly justified, which complicates interpretation of causal specificity.

17) The manuscript does not clearly state whether harmonisation procedures accounted for strand ambiguity, palindromic SNPs, or allele frequency thresholds. Given the large number of instruments and outcomes, explicit documentation of harmonisation steps is essential for reproducibility.

18) The use of inverse-variance weighted (IVW) MR as the primary estimator assumes no directional pleiotropy. However, given the extensive overlap of genetic variants across metabolite instruments (as shown in supplementary figures), this assumption is likely violated. The rationale for retaining IVW as the primary analysis, rather than a pleiotropy-robust estimator, should be more strongly justified.

19) The manuscript does not report heterogeneity statistics (e.g., Cochran's Q) consistently alongside MR estimates, despite mentioning their calculation. Without systematic reporting, it is difficult to assess the reliability of pooled MR estimates across instruments.

20) The assumption of gene-environment equivalence is central to the paper's conceptual framework, yet the statistical analysis does not formally test whether genetic and intervention-induced metabolite changes are comparable in magnitude or direction beyond correlation plots. A more explicit quantitative comparison would strengthen this claim.

Minor Comments

21) The scaling of metabolite measures differs between platforms (rank-normalised SD for MS metabolites and raw SD for NMR metabolites). While this is described, the implications for comparability of effect sizes across platforms are not discussed and should be clarified.

22) The description of Deming regression used for cross-intervention and cross-platform comparisons is brief. Additional details on error variance specification and assumptions would improve transparency.

23) Missing data handling is stated as "not applicable" for MR analyses, but this should be explicitly justified by clarifying that complete-case summary statistics were used throughout.

24) Confidence intervals are not consistently reported alongside point estimates in all MR-related tables and figures. Consistent reporting of uncertainty measures is recommended.

25) The description of rank normalisation for MS metabolites lacks detail on whether this was performed within cohorts, across platforms, or globally, which has implications for interpretability of effect sizes.

26) It is unclear whether the same genetic instruments were used across all heart failure outcomes or whether outcome-specific filtering was applied due to SNP availability. This should be clarified.

27) The Methods section would benefit from explicitly stating the minimum number of SNPs required to perform MR for a given metabolite and how metabolites with fewer instruments were handled.

28) The approach to outlier detection (e.g., removal of influential SNPs) is not described. Methods such as MR-PRESSO or leave-one-out diagnostics are mentioned only briefly and could be better integrated into the analytical workflow.

29) The statistical analysis does not explicitly state whether all analyses were pre-specified or whether some were exploratory. This distinction is important given the breadth of analyses conducted.

30) The reporting of p-values without corresponding effect sizes in some supplementary materials reduces interpretability and should be avoided.

31) The Methods section would benefit from a concise schematic or table summarising the full analytical pipeline, including data sources, estimands, statistical models, and sensitivity analyses

32) The manuscript would benefit from clearer notation distinguishing observational regression coefficients, intervention effect estimates, and MR causal estimates to avoid confusion across sections.

Reviewer #2: Summary

This manuscript aims to identify metabolic mediators linking weight loss to reduced risk of heart failure (HF) by integrating metabolomic profiling from two weight-loss interventions with Mendelian randomization (MR) analyses. The study combines data from dietary and surgical interventions with genetic instruments for BMI and metabolites to derive a set of "BMI-metabolites" and assess their potential causal role in HF. The study addresses an important and timely question and employs an ambitious multi-source analytical framework. The integration of metabolomics platforms (MS and NMR), as well as data from two randomized controlled trials, represents a clear strength. However, the manuscript currently suffers from substantial issues in clarity, methodological transparency, and causal interpretation, which limit its interpretability and impact.

Major Comments:

1. Clarity of the methodological framework

The manuscript lacks clarity regarding the core analytical strategy, and it takes considerable effort for the reader to understand what analyses were actually performed, what was the purpose and how the different components relate to each other.

It is often confusing, what is the main outcome variable and how is the exposure of interest defined. Also, the term "BMI-metabolites" is introduced without a sufficiently precise conceptual definition.

2. Causal interpretation and mediation claims

The authors frame the study as identifying mediators between weight loss and HF risk reduction. However, this interpretation is problematic.

*Causal mediation analysis generally relies on strong, untestable assumptions, which are not explicitly discussed.

*The analytical approach does not formally implement a mediation framework but instead combines association and MR analyses.

*Given issues such as pleiotropy and instrument non-specificity, the MR results should be interpreted cautiously and do not directly establish mediation.

Overall, the manuscript overstates the causal and mechanistic interpretation of the findings.

3. Insufficient description and transparency of statistical models

The statistical models are presented in a simplified form that ultimately reduces interpretability. For instance, the notation in Models 1, 2a, and 2b is unclear, as the outcome is indexed by i and t, but covariates on the right-hand side are not.

4. Identification of intervention effects vs. time trends

In Models 1 and 2b, it is unclear how the authors distinguish between time-related changes in metabolite levels (e.g., aging), and true intervention effects. This limitation should be more explicitly addressed and discussed.

5. Choice of mixed-effects models with two timepoints

The use of linear mixed-effects models with only two timepoints raises questions. With baseline and follow-up only, modeling change scores would be equivalent and more transparent. The current formulation does not appear to provide additional benefit, while making interpretation more difficult. Importantly, this does not resolve the issue of separating intervention effects from temporal trends.

6. Limitations of Model 2a (RCT-based analysis)

Model 2a, which estimates the effect of the dietary intervention via interaction terms, is conceptually appropriate and probably the only model that would identify the intervention effect that is unconfounded by other time trends. However, as the sample size is relatively modest and a large number of metabolites are tested, it does not seem plausible to detect robust signals can be reliably detected. The reviewer was also unable to locate the corresponding results in the supplementary materials.

7. Ambiguity in Mendelian randomization design

The MR framework is not clearly described and raises several concerns, as it is unclear whether the primary exposures are BMI, metabolites, or both at different stages. Moreover, the use of genetic instruments for BMI to estimate effects on metabolites may be problematic, as BMI-associated variants may influence metabolites via behavioral or lifestyle pathways (e.g., diet), violating MR assumptions. These issues should be more clearly explained and critically discussed.

8. Multiple testing and robustness of MR findings

The results of the MR analyses linking metabolites to HF outcomes appear weak, as most reported associations are borderline nominally significant. It is unclear how multiple testing correction was applied at this stage.

Based on the presented results, only a very limited number of associations (e.g., total cholesterol in HDL particles) appear robust.

Summary:

The manuscript addresses an important question and employs a potentially valuable integrative approach. However, substantial revisions are required to clarify the methodological framework, improve transparency of statistical analyses, and discussion on causal interpretations and underlying assumptions.

I therefore recommend major revision

Reviewer #3: This is a well‑conducted but methodologically complex study that integrates metabolomic profiling from interventional weight‑loss studies with Mendelian randomization (MR) analyses using publicly available GWAS data to interrogate the role of adiposity‑related metabolites in heart failure (HF). The authors present a thoughtful and potentially impactful framework that triangulates evidence from dietary and surgical weight‑loss interventions, genetic instruments for lifelong BMI, and HF GWAS outcomes. The resulting set of circulating metabolites with putative causal associations with HF represents a valuable resource that may help prioritize future mechanistic studies and therapeutic targets relevant to both weight loss and HF prevention.

The authors aim to define a robust metabolomic signature of weight loss that is consistent across different weight‑loss modalities (dietary and surgical) and to distinguish metabolite changes reflective of underlying adiposity biology while minimizing study‑specific confounding. This is achieved by integrating intervention‑derived metabolomic changes with genetic evidence capturing lifelong BMI exposure. The authors then test whether metabolites linked to BMI and weight loss are causally associated with HF and HF subtypes using MR applied to HF GWAS and prospective cohort datasets with HF outcomes.

Metabolomic profiling was performed in two independent weight‑loss intervention cohorts—a dietary weight‑loss trial and a bariatric surgery trial—using untargeted mass spectrometry and ¹H‑NMR platforms. Metabolite changes were modeled in relation to weight loss, and findings were integrated with GWAS‑based MR estimates of the lifelong effects of BMI on metabolites. Two‑sample MR analyses were then used to test BMI‑related metabolites for associations with HF outcomes, with exploratory observational validation conducted in external population cohorts (ARIC and UK Biobank).

The authors show that weight loss profoundly alters the circulating metabolome, with approximately 42-65% of measured metabolites changing following dietary or surgical intervention. A total of 240 metabolites demonstrated consistent directional change across both weight‑loss modalities, and 153 metabolites—enriched for lipids and lipoproteins, amino acids, and energy‑related metabolites—showed concordant effects across dietary weight loss, surgical weight loss, and genetically predicted lifelong BMI. Of these, 44 metabolites showed evidence of a causal association with HF in MR analyses. Notable findings include N‑acetylglycine and asparagine, which were inversely associated with BMI and HF risk (protective), and butyrylcarnitine (C4) and LDL‑related traits, which were associated with increased HF risk.

Observational evidence from Framingham and other prospective cohorts has long demonstrated that increasing BMI is associated with elevated risk of both HFrEF and HFpEF. MR Genome-wide PheWAS analyses of BMI further support a causal relationship between adiposity and many hard cardiovascular outcomes including HFpEF and HFrEF. Consistent with this, recent GLP‑1 receptor agonist randomized trials (e.g., SELECT) and meta‑analyses demonstrate that pharmacologically induced weight loss is associated with improvements in HF‑related symptoms (e.g., KCCQ scores) and trends toward reductions in cardiovascular and HF events, particularly in HFpEF populations.

Specific Comments

1. Use of UK Biobank in validation analyses: UK Biobank is a prominent contributor to the HERMES GWAS consortia used here for MR metabolite HF analyses. Although the directly measured metabolite study in UK Biobank is distinct from HERMES GWAS, the manuscript should acknowledge use of UKB (and presumably use of many of the same HF cases) in both the MR GWAS analysis and the validation analysis, and discuss the potential limitations in interpretation of findings thereof. This is particularly relevant given that the ARIC and UK Biobank directly measured metabolite analyses are appropriately described as exploratory post‑hoc analyses.

2. HFpEF versus HFrEF subtyping and statistical power: The HERMES HF GWAS meta‑analysis is dominated by broadly defined HF cases, with a relatively small proportion of participants classified into HFpEF or HFrEF subtypes (approximately 5% of total HF cases: ~5,406 HFrEF and ~3,841 HFpEF, derived from a non‑ischemic subset). However, clear diagnostic distinction among HFpEF, HFmrEF, and HFrEF are central to guideline‑directed therapy in clinical practice in US and EU and globally. As the authors note, HFpEF is more strongly linked to obesity, whereas evidence supporting weight‑loss benefit in HFrEF remains limited. Emerging data from GLP‑1 trials suggest benefits primarily in HFpEF rather than HFrEF. Given these considerations, the authors should more explicitly acknowledge the limited statistical power for subtype‑specific inference. In addition, the authors may wish to comment on whether summary statistics from the Million Veteran Program HFpEF/HFrEF GWAS (doi: 10.1038/s41467‑022‑35323‑0) were considered or could be incorporated to extend subtype‑specific MR analyses.

3. Incident versus prevalent HF and clarity of outcome definitions: HERMES is largely a case-control GWAS meta‑analysis of prevalent HF and should not be stated to be an incident HF GWAS. In contrast, ARIC and UK Biobank represent cohorts with incident HF ascertainment but are used here in exploratory post‑hoc analyses. To avoid potential reader confusion, I recommend omitting the term "incident HF" from the abstract and explicitly mentioning in the abstract that the HERMES GWAS meta-analysis is the primary HF GWAS data source.

4. Contemporary clinical context: GLP1R therapies: The manuscript mentions GLP1R in just one sentence in the discussion (citing reference 50, no clinical trials are cited), despite the absolutely explosive and evolving clinical landscape in which GLP1 receptor agonist therapies are now widely used in cardiovascular therapeutics. Given the strong relevance of pharmacologic weight loss to HF prevention—particularly HFpEF, greater mention of this study in the context of GLP1 receptor therapies would strengthen both the Introduction and Discussion including greater discussion of relevance (or lack thereof?) to the metabolite findings. If the authors intend to address GLP‑1‑related metabolomic effects in a separate manuscript, this could be stated explicitly for transparency.

5. Additional limitations: Beyond the limited power for HF subtype analyses, additional limitations that merit possible mention are (i) residual horizontal pleiotropy of metabolite instruments despite sensitivity analyses;

(ii) the possibility that identified metabolites act as both mediators and biomarkers rather than direct causal agents; and (iii) the use of rank‑normalized metabolite measures, particularly for MS‑based metabolites, which limits direct physiological and clinical interpretability.

Overall, this is a complex but carefully executed and innovative study that meaningfully advances our understanding of the metabolomic pathways linking adiposity, weight loss, and heart failure. Addressing the points above would further strengthen the manuscript's clarity, interpretability, and clinical relevance.

Any attachments provided with reviews can be seen via the following link: [LINK]

--------------------------------------------------------- ---

General editorial requests:

*Please ensure that the paper adheres to the PLOS Data Availability Policy (see http://journals.plos.org/plosmedicine/s/data-availability), which requires that all data underlying the study's findings be provided in a repository or as Supporting Information. For data residing with a third party, authors are required to provide instructions with contact information (web or email address) for obtaining the data. Please note that a study author cannot be the contact person for the data. PLOS journals do not allow statements supported by "data not shown" or "unpublished results." For such statements, authors must provide supporting data or cite public sources that include it.

* Currently your Data Availability statement does not adhere to our policy. When data is not legally allowed to be shared openly, please provide details on how researchers could ask for access. This needs to include contact details (email/URL), and the point of contact is not allowed to be the an author on the study. Please amend according to our policy.

*We expect all researchers with submissions to PLOS in which author-generated code underpins the findings in the manuscript to make all author-generated code available without restrictions upon publication of the work. In cases where code is central to the manuscript, we may require the code to be made available as a condition of publication. Authors are responsible for ensuring that the code is reusable and well documented. Please make any custom code available, either as part of your data deposition or as a supplementary file. Please add a sentence to your data availability statement regarding any code used in the study, e.g. "The code used in the analysis is available from Github [URL] and archived in Zenodo [DOI link]" Please review our guidelines at https://journals.plos.org/plosmedicine/s/materials-software-and-code-sharing and ensure that your code is shared in a way that follows best practice and facilitates reproducibility and reuse. Because Github depositions can be readily changed or deleted, we encourage you to make a permanent DOI'd copy (e.g. in Zenodo) and provide the URL.

* Thank you for providing your financial disclosure. Please also include URLs to the funders' websites.

* Ethics statement: Please include the IRB approval number for the DiRECT study.

FORMATTING - GENERAL

*Abstract: Please structure your abstract using the PLOS Medicine headings (Background, Methods and Findings, Conclusions). Please combine the Methods and Findings sections into one section.

* Abstract: Please include a statement on the main limitations of your study at the end of the 'Methods and Findings' section.

*At this stage, we ask that you include a short, non-technical Author Summary of your research to make findings accessible to a wide audience that includes both scientists and non-scientists. The Author Summary should immediately follow the Abstract in your revised manuscript. This text is subject to editorial change and should be distinct from the scientific abstract. Ideally each sub-heading should contain 2-3 single sentence, concise bullet points containing the most salient points from your study. In the final bullet point of 'What Do These Findings Mean?', please include the main limitations of the study in non-technical language. Please see our author guidelines for more information: https://journals.plos.org/plosmedicine/s/revising-your-manuscript#loc-author-summary.

*Please express the main results with 95% CIs as well as p values. When reporting p values please report as p<0.001 and where higher as the exact p value p=0.002, for example. Throughout, suggest reporting statistical information as follows to improve clarity for the reader "22% (95% CI [13%,28%]; p</=)". Please be sure to define all numerical values at first use.

*Please include line numbers in the manuscript file. Use continuous line numbers (do not restart the numbering on each page).

*Please cite the reference numbers in square brackets. Citations should precede punctuation.

FIGURES AND TABLES

*Please define all abbreviations used in each figure/table (including those in Supporting Information files).

*Please consider avoiding the use of red and green in order to make your figure more accessible to those with color blindness.

SUPPLEMENTARY MATERIAL

*Please note that supplementary material will be posted as supplied by the authors. Therefore, please amend it according to the relevant comments outlined here.

*Please cite your Supporting Information as outlined here: https://journals.plos.org/plosmedicine/s/supporting-information

REFERENCES

*PLOS uses the numbered citation (citation-sequence) method and first six authors, et al.

*Please ensure that journal name abbreviations match those found in the National Center for Biotechnology Information (NCBI) databases (http://www.ncbi.nlm.nih.gov/nlmcatalog/journals), and are appropriately formatted and capitalised.

*Where website addresses are cited, please include the complete URL and specify the date of access (e.g. [accessed: 12/06/2023]).

*Please also see https://journals.plos.org/plosmedicine/s/submission-guidelines#loc-references for further details on reference formatting.

MENDELIAN RANDOMIZATION STUDIES

*In the Introduction, please describe the exposure and the evidence for a potential causal relationship between exposure and outcome.

*In the Methods, please explicitly state the 3 core instrumental variable assumptions for the main analysis (relevance, independence, and exclusion restriction), as well assumptions for any additional or sensitivity analysis.

*In the Methods, please describe the MR estimator (e.g., 2-stage least squares, Wald ratio) and related statistics. Detail the included covariates and, in case of 2-sample MR, whether the same covariate set was used for adjustment in the 2 samples.

*If you are presenting an instrumental variable estimate, please compare this to the conventional observational estimate.

*Report the associations between genetic variant and exposure and between genetic variant and outcome, preferably on an interpretable scale.

*Report MR estimates of the relationship between exposure and outcome and the measures of uncertainty from the MR analysis, on an interpretable scale, such as odds ratio or relative risk per SD difference.

*If relevant, please consider translating estimates of relative risk into absolute risk for a meaningful time period.

*Please consider including plots to visualize results (e.g., forest plot, scatterplot of associations between genetic variants and outcome vs between genetic variants and exposure).

# Author comment: Author response to Decision Letter 2

**DOI:** 10.1371/journal.pmed.1005290.r003

15 Jun 2026

Attachment Submitted filename: 20260609_plosmed_reviewer_responses.pdf

# Peer review: Decision Letter 2

**DOI:** 10.1371/journal.pmed.1005290.r004

**Authors:** Suzanne De Bruijn

21 Aug 2026

Dear Dr SUNDERLAND,

Many thanks for submitting a revised version of your manuscript "A weight-loss metabolomic signature shows divergent associations across BMI-linked diseases" (PMEDICINE-D-25-04321R2) to PLOS Medicine. The paper has been seen again by 2 of the original reviewers; their comments are included below and can also be accessed here: [LINK]. Furthermore, we have also asked reviewer Reviewer #1 to comment on the rebuttal to the concerns of reviewer #2; these comments can also be found below.

As you will see, the reviewers thought the manuscript was much improved, but have some requests for additional textual revisions. After discussing the paper with the editorial team and an academic editor with relevant expertise, I'm pleased to invite you to revise the paper in response to the reviewers' comments. We plan to send the revised paper to some or all of the original reviewers, and we cannot provide any guarantees at this stage regarding publication.

Furthermore, we have some editorial requests, which you can find at the bottom of this email. Please include these in your point-by-point response as well.

In addition to these revisions, you may need to complete some formatting changes, which you will receive in a follow up email. A member of our team will be in touch with a set of requests shortly. If you do not receive a separate email within a few days, please assume that checks have been completed, and no additional changes are required.

When you upload your revision, please include a point-by-point response that addresses all of the reviewer and editorial points, indicating the changes made in the manuscript and either an excerpt of the revised text or the location (eg: page and line number) where each change can be found. Please also be sure to check the general editorial comments at the end of this letter and include these in your point-by-point response. When you resubmit your paper, please include a clean version of the paper as the main article file and a version with changes tracked as a marked-up manuscript. It may also be helpful to check the guidelines for revised papers at http://journals.plos.org/plosmedicine/s/revising-your-manuscript for any that apply to your paper.

We ask that you submit your revision by Sep 11 2026 11:59PM. However, if this deadline is not feasible, please contact me by email, and we can discuss a suitable alternative.

Don't hesitate to contact me directly with any questions (sbruijn@plos.org).

Best regards,

Suzanne

Suzanne De Bruijn, PhD

Associate Editor

PLOS Medicine

sbruijn@plos.org

-----------------------------------------------------------

Comments from the reviewers:

Reviewer #1: I thank the authors for their detailed responses and substantial revision of the manuscript. The revised version addresses the majority of the methodological and reporting concerns raised in the previous review. The analytical framework has been substantially clarified through restructuring of the Methods and Results sections, addition of an overview schematic, clearer separation of intervention and Mendelian randomization analyses, implementation of multiple-testing correction, and a more cautious interpretation of causal findings.

The manuscript has been considerably strengthened. However, I suggest the authors consider the following minor points:

a)The newly added clustering analysis based on shared genetic instruments improves interpretation of correlated metabolite signals. However, it does not fully address the potential for correlated metabolites within clusters to represent the same underlying biological signal. The limitations of interpreting clustered metabolites as independent findings could be acknowledged more explicitly in the Discussion.

b) The authors state that there is no overlap between exposure and outcome GWAS datasets. It would be helpful to provide a more explicit justification or supporting references documenting the contributing cohorts to reassure readers that bias from sample overlap is unlikely.

c) The rationale for applying FDR correction within analytical strata has been clarified and is reasonable. Nevertheless, the authors may wish to acknowledge that this approach is less conservative than a global correction across all analyses and could influence the interpretation of marginal findings.

Comments from Reviewer #1 on the concerns of reviewer #2:

I consider comments 1, 3, and 6 to have been fully addressed. Comments 2, 4, 5, and 7 have been addressed to a considerable extent, although I believe a few minor clarifications are still needed.

2.Causal and mechanistic interpretation

The language has been substantially moderated, particularly in the Discussion, where the authors explicitly state that the MR findings should not be interpreted as evidence that individual metabolites are directly causal. However, some wording still implies formal mediation or stronger causal inference than the analyses support. Most notably, the final box in Figure 1 states, “Distinct metabolite profiles mediate BMI’s effect on outcomes.” Similar language remains in places such as the Author Summary and the Discussion/conclusion, including references to metabolites “causally influencing” disease, “downstream metabolic mediators,” and pathways being isolated as downstream of adiposity and upstream of disease. I recommend replacing these remaining statements with wording such as “candidate pathways,” “putative mechanistic candidates,” or “metabolites associated with BMI-linked outcomes,” and explicitly stating that no formal mediation analysis was undertaken.

4.Intervention effects versus time trends

This is only partly addressed. Model 2 appropriately uses the timepoint-by-allocation interaction in DiRECT to estimate differential change between the randomized groups and therefore controls for temporal changes common to both groups. For the single-arm bariatric surgery analysis and intervention-only Model 1, however, the timepoint coefficient remains a within-group pre–post change and cannot distinguish an intervention effect from secular, ageing-related, or other time-varying changes. Although this is acknowledged in the response letter and can be inferred from the Methods, it is not stated explicitly in the Discussion limitations as claimed. I suggest adding an explicit sentence to the limitations and referring to the Model 1 coefficient as a “within-group change following intervention” rather than an identified intervention effect.

5.Choice of mixed-effects models with two timepoints

The rationale for retaining the mixed-effects model is reasonable because it allows participants with incomplete repeated measurements to contribute information. Nevertheless, the manuscript should state that this approach relies on an appropriate missing-at-random assumption. It would also be helpful to acknowledge that, among participants with complete data, the estimate is broadly equivalent to a change-score analysis. The authors should make clear that using a mixed model does not itself resolve the temporal-trend limitation identified in comment 4.

7.Mendelian randomization design

The structural ambiguity has been addressed: the manuscript now clearly separates BMI-to-metabolite MR from metabolite-to-outcome MR and identifies the exposure, outcome, instruments, and estimand at each stage. However, the specific concern about BMI-associated variants influencing metabolites through behavioural or lifestyle pathways that are not mediated by BMI has not been adequately discussed in the manuscript. The general description of MR assumptions and sensitivity analyses is helpful, but Steiger filtering and convergence across study designs cannot exclude this form of horizontal pleiotropy. Moreover, MR-PRESSO is described for the metabolite-to-outcome stage rather than as a solution to this concern in the BMI-to-metabolite stage. I recommend adding this as an explicit limitation and clarifying that triangulation reduces, but does not eliminate, uncertainty regarding instrument specificity.

Reviewer #3: this reviewer stated the manuscript can can be accepted.

Any attachments provided with reviews can be seen via the following link: [LINK]

--------------------------------------------------------- ---

General editorial requests:

* Please confirm that your title complies with PLOS Medicine's style. Your title must be nondeclarative and not a question. It should begin with main concept if possible. "Effect of" should be used only if causality can be inferred, i.e., for an RCT. Please place the study design ("A randomized controlled trial," "A retrospective study," "A modelling study," etc.) in the subtitle (ie, after a colon).

* Please confirm that your abstract complies with our requirements, including format (three sections: Background, Methods and Findings, and Conclusions) and providing all the information relevant to this study type https://journals.plos.org/plosmedicine/s/submission-guidelines#loc-abstract

* Please ensure that all abbreviations are defined at first use throughout the text.

* Please confirm that all numbers presented in the abstract are present and identical to numbers presented in the main manuscript text.

* Please include a statement regarding use or non-use of AI tools and technologies at the end of the Methods. Please specify the name(s) and version(s) of any tool(s) used and a description of their use, including how the AI-assisted or generated content was evaluated by authors, and a clear statement of which aspects of the study, article contents, data, or supporting files were affected or generated by AI tool usage. Please also include a statement specifying who retains responsibility for the final manuscript. See our guidelines for more information: https://journals.plos.org/plosmedicine/s/ethical-publishing-practice#loc-artificial-intelligence-tools-and-technologies.

* Funding statement: thank you for including this. Reading through it, it seems very extensive. Please consider limiting this section to only include funding for this specific study.

DATA AVAILABILITY

* PLOS Medicine requires that the de-identified data underlying the specific results in a published article be made available, without restrictions on access, in a public repository or as Supporting Information at the time of article publication, provided it is legal and ethical to do so. Please see the policy at http://journals.plos.org/plosmedicine/s/data-availability

and FAQs at http://journals.plos.org/plosmedicine/s/data-availability#loc-faqs-for-data-policy.

* PLOS defines the “minimal data set” to consist of the data set used to reach the conclusions drawn in the manuscript with related metadata and methods, and any additional data required to replicate the reported study findings in their entirety. Authors do not need to submit their entire data set, or the raw data collected during an investigation. Please submit the following data:

-The values behind the means, standard deviations and other measures reported;

-The values used to build graphs.

* The Data Availability Statement (DAS) requires revision. For each data source used in your study:

a) If the data are freely or publicly available, note this and state the location of the data: within the paper, in Supporting Information files, or in a public repository (include the DOI or accession number).

b) If the data are owned by a third party but freely available upon request, please note this and state the institutional owner of the data set and contact information for data requests (institutional web or email address). Note that a study author cannot be the contact for the data.

c) If the data are not freely available, please describe briefly the ethical, legal, or contractual restriction that prevents you from sharing it. Please also include an appropriate institutional contact (institutional web or email address) for inquiries (again, this cannot be a study author).

We appreciate your statement that the data from both studies are not freely available, but please specify where the data can be requested, taking into account this cannot be a study author. Furthermore, please also include where the GWAS statistics used for the MR analysis can be found.

* Thank you for including the code availability statement in your manuscript. Please also include this text in the metadata, as part of the data aviailability statement.

FIGURES

* When a p value is given, please specify the statistical test used to determine it in the legend.

* Please consider avoiding the use of red and green in order to make your figure more accessible.

MENDELIAN RANDOMIZATON studies

* Please ensure that the study is reported according to the STROBE-MR guideline, and include the completed STROBE-MR checklist as Supporting Information. Please add the following statement, or similar, to the Methods: "This study is reported as per the Strengthening the Reporting of Observational Studies in Epidemiology (STROBE) guideline, specific for mendelian randomization (S1 Checklist).

Thank you for including the STROBE-MR checklist. Please add a statement to the method citing this checklist (as above). Please also amend the checklist to use section and paragraph numbers, rather than page numbers.

# Author comment: Author response to Decision Letter 3

**DOI:** 10.1371/journal.pmed.1005290.r005

29 Sep 2026

Attachment Submitted filename: plosmed_reviewer_responses_v0.3.docx

# Peer review: Decision Letter 3

**DOI:** 10.1371/journal.pmed.1005290.r006

**Authors:** Suzanne De Bruijn

8 Oct 2026

Dear Dr. SUNDERLAND,

Thank you very much for re-submitting your manuscript "A metabolomic signature of weight-loss and its associations with BMI-linked diseases: an analysis of two intervention trials and a Mendelian randomization study" (PMEDICINE-D-25-04321R3) for review by PLOS Medicine.

I have discussed the paper with my colleagues and the academic editor and it was also seen again by one reviewer. I am pleased to say that provided the remaining editorial and production issues are dealt with we are planning to accept the paper for publication in the journal.

The remaining issues that need to be addressed are listed at the end of this email. Any accompanying reviewer attachments can be seen via the link below. Please take these into account before resubmitting your manuscript:

[LINK]

***Please note while forming your response, if your article is accepted, you may have the opportunity to make the peer review history publicly available. The record will include editor decision letters (with reviews) and your responses to reviewer comments. If eligible, we will contact you to opt in or out.***

In revising the manuscript for further consideration here, please ensure you address the specific points made by the editors. In your rebuttal letter you should indicate your response to the reviewers' and editors' comments and the changes you have made in the manuscript. Please submit a clean version of the paper as the main article file. A version with changes marked must also be uploaded as a marked up manuscript file.

Please also check the guidelines for revised papers at http://journals.plos.org/plosmedicine/s/revising-your-manuscript for any that apply to your paper. If you haven't already, we ask that you provide a short, non-technical Author Summary of your research to make findings accessible to a wide audience that includes both scientists and non-scientists. The Author Summary should immediately follow the Abstract in your revised manuscript. This text is subject to editorial change and should be distinct from the scientific abstract.

In addition to these revisions, you may need to complete some formatting changes, which you will receive in a follow up email. A member of our team will be in touch with a set of requests shortly. If you do not receive a separate email within a few days, please assume that checks have been completed, and no additional changes are required.

We expect to receive your revised manuscript within 1 week. Please email us (plosmedicine@plos.org) if you have any questions or concerns.

Please review your reference list to ensure that it is complete and correct. If you have cited papers that have been retracted, please include the rationale for doing so in the manuscript text, or remove these references and replace them with relevant current references. Any changes to the reference list should be mentioned in the rebuttal letter that accompanies your revised manuscript.

Please note, when your manuscript is accepted, an uncorrected proof of your manuscript will be published online ahead of the final version, unless you've already opted out via the online submission form. If, for any reason, you do not want an earlier version of your manuscript published online or are unsure if you have already indicated as such, please let the journal staff know immediately at plosmedicine@plos.org.

If you have any questions in the meantime, please contact me or the journal staff on plosmedicine@plos.org.

We look forward to receiving the revised manuscript by Oct 15 2026 11:59PM.

Sincerely,

Suzanne De Bruijn, PhD

Associate Editor

PLOS Medicine

plosmedicine.org

------------------------------------------------------------

Requests from Editors:

* Please modify your title to "A metabolomic signature of weight-loss and its associations with BMI-linked diseases: a post-hoc analysis of two intervention trials and a Mendelian randomization study".

* You don't need to provide the protocol as supplementary material. As we cannot include a published paper as supplementary material, please remove this file (PIIS2213858725000257.pdf) and instead cite the paper where necessary.

* Funding statement: Please remove the text "For the purpose of Open Access, the author has applied a CC BY public copyright license to any Author Accepted Manuscript version arising from this submission". Although this is true, it does not need to be in the funding statement.

* Please ensure that the Zenodo DOI is included in the DAS (instead of the text “[DOI link to be confirmed after acceptance / confirmation that no further changes are required”]).

* Please confirm that the data have been deposited in the controlled access repository, and amend your data availability statement accordingly. We do not accept the statement "The data used for analysis will be placed on a research data repository", as the data has to be available at the time of publication.

* Please amend your Data Availability Statement to include that values underlying the graphs are present in the supplementary material.

Comments from Reviewers:

Reviewer #1: Thank you for thoroughly addressing all comments and providing detailed clarifications in the revised manuscript. I appreciate the effort you have put into enhancing the rigor and transparency of your analyses. The manuscript now reflects a robust and valuable contribution to subject, I am happy to recommend this work for publication and look forward to seeing its positive impact in the field.

Any attachments provided with reviews can be seen via the following link:

[LINK]

# Author comment: Author response to Decision Letter 4

**DOI:** 10.1371/journal.pmed.1005290.r007

19 Oct 2026

Attachment Submitted filename: plosmed_reviewer_responses_v0.4.docx

# Editor report: Decision Letter 4

**DOI:** 10.1371/journal.pmed.1005290.r008

**Authors:** Suzanne De Bruijn

21 Oct 2026

Dear Dr SUNDERLAND,

On behalf of my colleagues and the Academic Editor, Shiu Lun Au Yeung, I am pleased to inform you that we have agreed to publish your manuscript "A metabolomic signature of weight-loss and its associations with BMI-linked diseases: a post-hoc analysis of two intervention trials and a Mendelian randomization study" (PMEDICINE-D-25-04321R4) in PLOS Medicine.

Before your manuscript can be formally accepted you will need to complete some formatting changes, which you will receive in a follow up email. Please be aware that it may take several days for you to receive this email; during this time no action is required by you. Once you have received these formatting requests, please note that your manuscript will not be scheduled for publication until you have made the required changes.

In the meantime, please log into Editorial Manager at http://www.editorialmanager.com/pmedicine/, click the "Update My Information" link at the top of the page, and update your user information to ensure an efficient production process.

PRESS

We frequently collaborate with press offices. If your institution or institutions have a press office, please notify them about your upcoming paper at this point, to enable them to help maximise its impact. If the press office is planning to promote your findings, we would be grateful if they could coordinate with medicinepress@plos.org. If you have not yet opted out of the early version process, we ask that you notify us immediately of any press plans so that we may do so on your behalf.

We also ask that you take this opportunity to read our Embargo Policy regarding the discussion, promotion and media coverage of work that is yet to be published by PLOS. As your manuscript is not yet published, it is bound by the conditions of our Embargo Policy. Please be aware that this policy is in place both to ensure that any press coverage of your article is fully substantiated and to provide a direct link between such coverage and the published work. For full details of our Embargo Policy, please visit http://www.plos.org/about/media-inquiries/embargo-policy/.

Thank you again for submitting to PLOS Medicine. We look forward to publishing your paper.

Sincerely,

Suzanne De Bruijn, PhD

Associate Editor

PLOS Medicine
